# Robust and sensitive amplicon-based whole-genome sequencing assay of respiratory syncytial virus subtype A and B

**DOI:** 10.1128/spectrum.03067-23

**Published:** 2024-02-27

**Authors:** Tiina Talts, Lucy G. Mosscrop, David Williams, John S. Tregoning, Whitney Paulo, Arinder Kohli, Thomas C. Williams, Katja Hoschler, Joanna Ellis, Simon de Lusignan, Maria Zambon

**Affiliations:** 1 UK Health Security Agency, London, United Kingdom; 2 Imperial College London, London, United Kingdom; 3 University of Edinburgh, Edinburgh, United Kingdom; 4 University of Oxford, Oxford, United Kingdom; Emory University School of Medicine, Georgia, USA

**Keywords:** respiratory syncytial virus, whole-genome sequencing, next-generation sequencing

## Abstract

Prevention of respiratory syncytial virus (RSV) infection is now a global health priority, with a long-acting monoclonal antibody and two RSV vaccines recently licenced for clinical use. Most licenced and candidate interventions target the RSV fusion (RSV-F) protein. New interventions may be associated with the spread of mutations, reducing susceptibility to antibody neutralization in RSV-F. There is a need for ongoing longitudinal global surveillance of circulating RSV strains. To achieve this large-scale genomic surveillance, a reliable, high-throughput RSV sequencing assay is required. Here we report an improved high-throughput RSV whole-genome sequencing (WGS) assay performed directly on clinical samples without additional enrichment, using a 4-primer-pool, short-amplicon PCR-tiling approach that is suitable for short-read sequencing platforms. Using upper respiratory tract (URT) RSV-positive clinical samples obtained from a sentinel network of primary care providers and from hospital patients (29.7% and 70.2%, respectively; *n* = 1,037), collected over the period 2019 to 2023, this assay had a threshold of approximately 4 × 10^3^ to 8 × 10^3^ copies/mL (RSV-B and RSV-A sub-types, respectively) as the lowest amount of virus needed in the sample to achieve >96% of whole-genome coverage at a high-quality level. Using a Ct value of 31 as an empirical cut-off, the overall assay success rate of obtaining >90% genome coverage at a read depth minimum of 20 was 96.83% for clinical specimens successfully sequenced from a total of 1,071. The RSV WGS approach described in this study has increased sensitivity compared to previous approaches and can be applied to clinical specimens without the requirement for enrichment. The updated approach produces sequences of high quality consistently and cost-effectively, suitable for implementation to underpin national programs for the surveillance of RSV genomic variation.

**IMPORTANCE:**

In this paper, we report an improved high-throughput respiratory syncytial virus (RSV) whole-genome sequencing (WGS) assay performed directly on clinical samples, using a 4-primer-pool, short-amplicon PCR-tiling approach that is suitable for short-read sequencing platforms. The RSV WGS approach described in this study has increased sensitivity compared to previous approaches and can be applied to clinical specimens without the requirement for enrichment. The updated approach produces sequences of high quality consistently and cost-effectively, suitable for implementation to underpin national and global programs for the surveillance of RSV genomic variation. The quality of sequence produced is essential for preparedness for new interventions in monitoring antigenic escape, where a single point mutation might lead to a reduction in antibody binding effectiveness and neutralizing activity, or indeed in the monitoring of retaining susceptibility to neutralization by existing and new interventions.

## INTRODUCTION

Interventions for the respiratory syncytial virus (RSV) are gradually being introduced to clinical practice: the prolonged half-life monoclonal antibody (Nirsevimab) was licensed in 2022/2023; vaccines for the elderly have been licensed in 2023 (1, 2) and several different vaccines are expected to come into use by 2025 (3). Virological monitoring of circulating strains will be critical in evaluating the impact of these specific interventions on viral evolution and tracking the dissemination of variants capable of escaping interventions. Such mutations have already been seen *in vitro* and clinically in patients treated prophylactically with Palivizumab and Motavizumab (4). Systematic and timely global genomic initiatives such as that of studying RSV genomic variability are required to detect the emergence of mutations that may confer drug resistance or antigenic escape arising from selective pressure from newly introduced interventions (5).

The SARS-CoV-2 pandemic has shown that large-scale viral genomic surveillance is achievable worldwide, with over 15 million complete SARS-CoV-2 whole genomes generated from over 170 countries published between 2020 and 2023 (6). Although the number of publicly accessible whole-genome sequences (WGS) for RSV is very limited in comparison to SARS-CoV-2, this is likely to expand exponentially in the coming years. Most RSV genetic studies to date have focused on analyzing variations in the highly variable RSV attachment protein, RSV G (7, 8) which is commonly used for genotyping, although vaccines and monoclonals target the F protein, and small molecule antivirals in development may target other viral targets such as L protein, indicating that systematic surveillance targeting the entire viral genome is most appropriate going forward. One of the earliest RSV WGS of primary clinical specimens was obtained using a 15-amplicon-PCR-based strategy (9). Since then multiple RSV WGS methods have been published (10–12). WGS is also likely to yield useful information to understand whether RSV circulation and seasonality are affected by large-scale immunization campaigns. The development of incrementally improved, rapid, and higher-throughput WGS methodologies, which can be used to track RSV virus evolution at a global level, is required.

In this study, we report a sensitive, improved, high-throughput WGS assay for use directly with clinical samples without any additional enrichment procedures, using a 4-primer-pool, short-amplicon PCR-tiling approach similar to those used for SARS-CoV-2 (13).

## MATERIALS AND METHODS

### Samples and data collection

#### Source population: primary care

URT specimens were obtained using community-based sampling of patients of all ages presenting with Influenza-like illness (ILI) through a sentinel network of General Practitioners (RCGP; (14)), representative of population and geography in England, as part of national respiratory virus surveillance. RSV inclusion in the testing scheme included years from 2008 to present where combined nose and throat swabs were generally collected within 7 days, with sampling criteria broadened to include children under 5 years with acute bronchitis and bronchiolitis. The sampling frame was broadened to include lower respiratory infections in 2020, and the onset date lengthened to 10 days. There was a further extension to include upper respiratory infections in 2021 (15, 16).

#### Source population: secondary care

Samples received from secondary care (hospitalized children) from 2019 onwards were from:

1. Sentinel hospitals within England established to pilot RSV sampling and surveillance.
2. Samples from a national multi-center prospective observational cohort study provided specimens of children under 2 years of age attending emergency departments (EDs) across the UK (17).

UK public health agencies have permission to process patient confidential information for national surveillance of communicable diseases under Regulation 3 of the Health Service Regulation 2002 for England, the Public Health (Scotland) Act 2008 and the NHS Scotland Act 1978 for Scotland, and the Public Health Wales National Health Service Trust (Establishment) Order 2009 for Wales.

### Virus isolation methods

Human cervix carcinoma HEp-2 (HeLa derivative) (ECACC, 86030501) cells were cultured in growth medium containing the following: Opti-MEM Reduced-Serum Medium (Gibco, Fisher Scientific) supplemented with 10% (vol/vol) fetal bovine serum (FBS; Gibco, Fisher Scientific) and with added antibiotics and antimycotic [1× Penicillin-Streptomycin-Glutamine (Gibco, Fisher Scientific) and 1× Amphotericin B (Gibco, Fisher Scientific), respectively]. Post-inoculation medium had a reduced FBS content of 2% (vol/vol). Cell culture compatible flat-bottomed tubes with screw caps with 5.5 cm^2^ culture area for growth of adherent cells (Thermo Scientific Nunc) were used for virus isolation from nasopharyngeal swab specimens collected in virus transport medium [Bovine Albumin 20% wt/vol (Sigma-Aldrich), Penicillin G Sodium Salt 10^6^ Units (Sigma-Aldrich), Streptomycin Sulfate 10^6^ Units (Sigma-Aldrich), Hanks’ Balanced Salt Solution (HBSS) (Thermo Scientific), deionized water; pH adjusted to 6.8–6.9]. HEp-2 cells were seeded with a density of 1.6 × 10^5^ cells/mL, 2 mL per tube for next-day inoculation with 85%–90% confluency. Cells were inoculated with 200 µL of clinical specimen and adsorbed with shaking every 15 min for 2 h at 33°C–34°C. The inoculum was aspirated, 2 mL of post-inoculation medium was added to each tube and tubes were incubated in 37°C ± 2°C 5% CO_2_ incubator until cytopathic effect (CPE) formation for up to 7 days. The virus was harvested by scraping the cell layer and the suspension was sonicated in an ice-water bath with intermittent bursts for 15 min. Sonicated suspension was frozen and stored at −80°C until use. The virus titer was determined as described below.

### Virus stock culture methods

African green monkey kidney VERO cells (ATCC, CCL 81) were cultured in a growth medium containing: Dulbecco’s Modified Eagle Medium (DMEM; Gibco, Fisher Scientific) supplemented with 10% (vol/vol) FBS (Gibco, Fisher Scientific) and with added antibiotics and antimycotic [1× Penicillin-Streptomycin-Glutamine (Gibco, Fisher Scientific) and 1× Amphotericin B (Gibco, Fisher Scientific), respectively]. Post-inoculation medium had a reduced FBS content of 2% (vol/vol). VERO cells were seeded with a density of 1.6 × 10^5^ cells/mL for next-day inoculation with a multiplicity of infection of 0.01 and incubated in 37°C ± 2°C 5% CO_2_ incubator until CPE formation for up to 7 days. The virus was harvested by scraping the cell layer and the suspension was sonicated in an ice-water bath with intermittent bursts for 15 min. The supernatant was frozen and stored at −80°C until use. The virus titer was determined as described below.

### Virus quantitation by plaque-forming units by immunostaining method

VERO cells (ATCC CCL 81) were seeded with a density of 10^5^ cells per well of a 24-well plate, in 1 mL of growth medium and incubated in 37°C ± 2°C 5% CO_2_ incubator until ~90% confluent (usually overnight is sufficient). The next day the growth medium was removed, and the cell layer was washed twice with serum-free medium prior to virus inoculation with 100 µL of 10-fold serial diluents of RSV, in triplicate wells. The cells were incubated in 37°C ± 2°C 5% CO_2_ incubator with shaking every 15 min for up to 2 h. After the inoculum was aspirated, 1 mL of overlay medium [1:1 2× DMEM (Thermo Fisher Scientific) and 1.2% (wt/vol) pre-autoclaved microcrystalline cellulose (Avicel, FMC) solution in ddH_2_O] was added to each well and the cells were incubated in 37°C ± 2°C 5% CO_2_ incubator for ~3 days (64–66 h). On day 3, cells were fixed by adding 1 mL of freezer cold fixing solution [Absolute Methanol with added H_2_O_2_ 0.6% (vol/vol) (Hydrogen peroxide (Sigma-Aldrich) 30% wt/wt in H_2_O)] and incubated at room temperature for 30 min. The overlay was aspirated, and cells were washed three times with distilled water. The non-specific antibody-binding sites were blocked by adding 1 mL of 5% (wt/vol) milk powder in phosphate-buffered saline (Marvel and Thermo Fisher Scientific, respectively) blotting solution and incubated by gently rocking at room temperature for 1 h. After removal of the blotting solution, it was replaced with 200 µL of 1/2,000 dilution of RSV A/B specific monoclonal primary antibody (mAB; Chemicon Int., MAB858-4) in blotting solution to each well and incubated by gently rocking at room temperature for 1 h. After the removal of the mAb solution, plates were washed three times with distilled water, followed by the addition of 200 µL of 1/2,000 dilution of secondary antibody [HRP-conjugated Goat-anti-mouse IgG (H + L) KPL, 074-1806] in blotting solution and incubated by gently rocking at room temperature for 40 min. After the removal of the solution, wells were washed three times, 200 µL of peroxidase substrate [KPL True Blue peroxidase substrate (Sera Care, 5510-0030)] with added H_2_O_2_ 0.1% (vol/vol) was added to each well and incubated until plaques could be visualized (up to 10 min). The substrate was removed and wells were washed with distilled water, plates were patted dry and left to dry completely, usually overnight, protected from light, for next-day imaging and recording using an ImmunoSpot Analyzer (Cellular Technology Ltd).

### Virus isolates used in analytical assessment

HEp2 (HeLa derivative) (ECACC, 86030501) cell culture clinical isolates were passaged in VERO (ATCC, CCL 81) cell culture for large volume stock propagation. These stock-cultured viruses were used for analytical sensitivity assessment (Table 1) after quantification of virus by plaque-forming units (pfu) using the immunostaining method. Extracted RNA was 10-fold serially diluted for batch preparation in bulk and portioned into single-use aliquots (stored at −80°C) to avoid freeze/thaw cycles between separate experiments throughout the whole sensitivity assessment process. Data of the respective dilutions and corresponding RSV RT-qPCR Ct values, dPCR absolute quantitation, and pfu values are available in (Table S1).

**TABLE 1:** Virus isolates used for analytical sensitivity assessment

Isolate/GISAID name	GISAID ID	Collection date	Isolate pfu/mL
hRSV/A/England/174820313/2017	EPI_ISL_732346	27/11/2017	6 × 10^5^
hRSV/B/England/174880237/2017	EPI_ISL_732349	30/11/2017	8 × 10^6^

### Sample processing and nucleic acid extraction

A volume of 150 µL of the clinical specimen or virus isolate material was inactivated in an equal volume of lysis buffer [NucliSENS easyMAG Lysis Buffer (1,000 mL) ref.280134; bioMérieux] that was spiked with a fragment of exogenous Soil-borne cereal mosaic virus (SBCMV) (gb:AJ298069) (18) synthetic transcript for internal process control purposes. The inactivated specimen preparations underwent nucleic acid extraction on either NucliSENS easyMAG (bioMérieux) or chemagic 360 (Perkin Elmer) extraction platform using NucliSENS easyMAG (bioMérieux) extraction reagents with a “Generic 2.0.1” protocol or Chemagic Viral DNA/RNA 300 Kit H96 (Perkin Elmer, CMG-1033-S), with default total nucleic acid elution volume of 100 µL per extracted specimen from any automated extraction platform.

### Analytical specificity panels

The viral RNA was extracted as described above and viral nucleic acid (NA) extracts were pooled in approximately equimolar quantities. The pool of viral NA of additional pathogens contained the following: Influenza: A(H1N1)pdm09, A(H3N2), B/Victoria, B/Yamagata, A(H1N2); Avian Influenza: A(H5N1), A(H7N9), A(H9N2), A(H5N6); hPIV types 1, 2, 3, and 4, hAdV C1, hAdV A31, hRhV A1, hRhV C9, hMPVA, hMPVB, hCoV-229E, hCoV-OC43, hCoV-NL63, hCoV-HKU1, MERS-CoV, SARS CoV-2, and SARS CoV-1 Hong Kong (Supplementary Supporting Information Table S2).

### RSV detection and subtyping of respiratory specimens

RSV detection and subtyping were performed using primers and probes targeting the nucleocapsid (N) gene from RSV in a multiplex format. A quadruplex RT-PCR consisted of generic RSV, human metapneumovirus (hMPV), and SBCMV amplification primers (Table 2) with RSV A and B subtype distinguishing fluorescent oligoprobes and probes for detection of hMPV and SCMV. Each reaction contained 5 µL of sample RNA, 5 µL of 4× TaqPath 1-Step Multiplex Master Mix with Mustang Purple as a passive reference (Thermo Fisher), each primer and fluorescent oligoprobe as in (Table 2), and molecular-grade double-distilled water to a volume of 20 µL. The cycling parameters consisted of an initial annealing step of 2 min at 25ºC and an RT extension cycle of 10 min at 53°C, followed by cDNA amplification initial denaturation step of 2 min at 95°C, followed by 40 cycles consisting of denaturation at 95°C for 3 s, annealing/extension at 60°C for 30 s with fluorescence data acquisition. The qualitative multiplex real-time RT-PCR assay was performed on the QuantStudio 7flex platform (Applied Biosystems).

**TABLE 2:** RSV-AB/hMPV/IC multiplex real-time RT-PCR oligonucleotide sequences and final reaction concentrations

Oligonucleotide	Sequence 5′−3′	Final concentration (nM)	Amplicon length (bp)	Manufacturer
RSV sense primer	MAG AGG KGG CAG TAG AGT TGA	600	62	Metabion
RSV antisense primer	CCT GMA CCA TAG GCA TTC AT	800		Metabion
RSV-A probe	AGG GAT TTT TGC AGG ATT G *Plus*	200		Biosearch Technologies
RSV-B probe	AGG AAT CTT TGC AGG ATT G *Plus*	250		Biosearch Technologies
hMPVA sense primer	CAG CAC CAG ACA CAC CTA TAA T	300	120	Metabion
hMPVA antisense primer	TGC ATC ACT TAG TAC ACG GTT AG	600		Metabion
hMPVA probe	AGT GGG ATT AGA GAC CAC AGT CAG AAG A	150		Thermo Fisher/ABI
hMPVB sense primer	GGG TGT CAT TGC CAG ATC AT	300		Metabion
hMPVB antisense primer	CCA GAT TCA GGA CCC ATT TCT C	300		Metabion
hMPVB probe	AGG GCA TGT ATC TGT GCA AGC TGA	150		Thermo Fisher/ABI
IPC SBCMV sense primer	CAC TCA GGA CGG TGA CGA GAT	50	80	Metabion
IPC SBCMV antisense primer	GTG ATA CTG TGA GTC TGG TGA TTT	500		Metabion
IPC SBCMV probe	TTT TGT GAC CTT GGA GGT GAG GCA GTT ATG	150		Metabion

### Quantification of viral nucleic acid by digital PCR

The digital PCR (dPCR) procedure was performed following the manufacturer’s instructions using the QIAcuity Four platform (Qiagen). All dPCRs were performed in a total reaction volume of 40 µL for 24-well (26 k partitions) Nanoplates (Qiagen, 250001), using the QIAcuity One-Step Viral RT-PCR Kit (Qiagen, 1123145). All reactions were set up as singleplex containing 0.6 µM forward primer, 0.8 µM reverse primer, and 0.25 µM of target probe with 5 µL of template. Each plate contained a no template control (NTC). Amplification conditions were as follows: 50°C for 40 min for reverse transcription, 95°C for 2 min for enzyme activation, 95°C for 5 s for denaturation, and 60°C for 30 s for annealing/extension for a total of 40 cycles). Imaging was carried out by reading in the Green/FAM channel.

Data acquisition and analysis were carried out using the QIAcuity software version 2.0.20. Thresholds were set based on the amplitude signal observed in negative control samples (19, 20). Experiments were performed on three separate occasions as duplicate replicates per experiment. The same threshold was applied to all wells per target of one PCR plate. The obtained quantity data with a confidence value range of 1.7%–6.7% and lambda range of 0.035–2.336 were included in the final quantitation calculations.

### Whole-genome sequencing primer scheme designs

RSV-A and RSV-B primer schemes were designed using the “primal scheme” (13) tool shared on GitHub (https://github.com/aresti/primalscheme). A fully editable version was used with a command line interface. Input for the tool included RSV-A and RSV-B sequences from globally submitted WGS sequences spanning 2018 to 2021 submissions to GISAID. A reference sequence pool was generated from 638 RSV-A and 802 RSV-B whole-genome sequences originating from countries, including Australia, Egypt, Russia, South Africa, the USA, and the UK (downloaded 26.05.2022).

A percent identity matrix was created using EMBL Clustal Omega web tool version 2.1 (21). The sequences were divided into two groups per subtype, based on their nucleotide sequence divergence across the whole genome. One scheme was designed per the resulting two groups of sequences per type where the maximum sequence divergence did not exceed 5%. Unipro UGENE v43.0 (22) with Levitsky consensus mode at 100% threshold was used to generate a flattened sequence mask.

The following RSV-A and RSV-B schemes were designed (Table 3):

**TABLE 3:** RSV-A/B WGS assay primer scheme^*a*^

Target	Scheme	Pool_id	Amplicon lengths	# Primers	# reactions per sample
**RSV-A**	RSV-A_1	#1	750…850 bp	26	4
		#2		26	
	RSV-A_2	#1	750…1000 bp	24	
		#2		24	
**RSV-B**	RSV-B_1	#1	630…690 bp	30	4
		#2		28	
	RSV-B_2	#1	650…700 bp	32	
		#2		30	

^*a*^ Primer sequences available on GitHub: https://github.com/tiinatalts/RSV_wgs.

### Whole-genome sequencing methods

The PCR protocol used was based on a modified version of the viral sequencing protocol which is available at

https://www.protocols.io/view/ncov-2019-sequencing-protocol-v3-locost-bp2l6n26rgqe/v3 (13). A reverse transcriptase reaction was applied to 12 µL of the extracted total nucleic acid using 3 µL of LunaScript RT SuperMix Kit (New England Biolabs, E3010L) with the following cycling conditions: 25°C for 2 min annealing step, 55°C for 10 min extension step followed by 95°C for 1 min denature step, and final hold at 4°C for store. Each cDNA sample underwent amplification in four PCRs containing 12.5 µL of Q5 Hot Start High-Fidelity 2 × MasterMix (New England Biolabs, M0494L), 6 µL of nuclease-free water, and 4 µL of each primer pool at 10 µM final concentration of RSV_A Scheme1_Pool1, Scheme1_Pool2, Scheme2_Pool1, and Scheme2_Pool2 for RSV-A and the equivalent for RSV-B sample. The following cycling conditions were used: denature at 98°C for 30 s followed by 35 cycles of 95°C for 15 s and 63°C for 5 min and final store at 10°C. As we used equal concentration, volume, and number of primers in each pool, no balancing was deemed necessary and the four PCRs were pooled in an equivolume manner. The pooled amplification reaction was subject to a clean-up procedure using AMPure XP Magnetic beads (Agencourt) and Biomek i5 automated liquid handler (Beckman Coulter) with final 70 µL elution with nuclease-free water. Library preparation was performed with Illumina Nextera XT and Illumina DNA Prep Sample Preparation Kits (Illumina, FC-131-1096 and 20018705) with IDT for Illumina Nextera DNA UD Indexes- Set A, B (96 Indexes, 96 samples: Illumina, 20027213, 20027214) using automated liquid handlers, G3 Sciclone (PerkinElmer), Biomek i7 (Beckman Coulter), Dragonfly discovery, and Mosquito HV genomics (SPT Labtech). Paired-end sequencing was performed on NextSeq500/550 and NextSeq1000 sequencers using NextSeq500/550 Mid output kit v2.5 (300/150 cycles; Illumina, 20024905/20024904) and NextSeq1000/2000 P2 Reagents (200 cycles v3; Illumina, 20046812), respectively.

### Genome assembly

The preassembly stage consisted of the demultiplexing process by the Illumina bcl2fastq 2.20 software and the removal of low-quality regions and adapters by trimmomatic (23). For the fastQ files of each sample, mapping against a reference sequence was performed using Burrows-Wheeler Aligner software (version 0.7.17-r1188) BWA-MEM algorithm ((24) preprint). pySAM was used to convert between SAM and BAM files and sort reads (version 1.15 using htslib 1.14; (25)). Duplicate reads were marked for exclusion from analysis using Picard (version picard-2.27.4–0) (26). Primer-derived sequences were excluded by soft-clipping the ends of mapped reads using iVar (version 1.3.1) (27). Consensus sequences and viral quasi-species quantification from bam files were generated with QuasiBam as described previously (in-house software; version 2.10 using bamtools-2.5.2 library) (28) with base calling with ≥15 read depth at a position and minimum proportion to affect an ambiguity was 20%.

### Procedures to determine sequence run validity

NextSeq500/550 and NextSeq1000NextSeq1000 local quality control (QC) metrics: run was considered to have passed if the run negative control (molecular biology grade water) has <10,000 reads, the run positive control (*Escherichia coli* K12) had an average meanQ of >30, a post-trim yield of ≥150 Mb, and a K-mer identification of >99%. With optimal cluster density for NextSeq500/550 of ~170–200 K/mm^2^ and estimated yield of 16.2525–39Gb for 300/150 cycles; and similarly for NextSeq1000 runs with ~80 Gb estimated yield for 300/150 cycles.

RSV WGS assay global QC metrics: an assay was considered passed if the negative control (added at the pre-sequencing amplification stage per each instance) had <1,000 high-quality (HQ) mapped filtered reads. The observed RSV WGS negative control median of the number of HQ mapped filtered reads was <20 per assay instance. The assay positive control (added at extraction stage; combined RSV-A and RSV-B each at ~30 × 10^3^ copies/mL) had the mean HQ mapped filtered reads >800,000 per each type.

### Statistics and data visualization

RStudio 2022.12.0 with R version 4.2.2 (29) was used for the data analysis. For statistical analysis, non-parametric tests were used to assess the distribution of the data: Anderson Darling test (Thode (30)), Kolmogorov-Smirnov test (31), and custom R script were used for resampling by bootstrapping when comparing differences in median values in clinical data sets stratified by age.

Probit logistic regression analysis was used to model binary outcome variables for determining the lower limit of detection (LLoD) for the RSV RT-qPCR with StatsDirect version 3.1.22.

Tidyverse ggplot2 (32) in RStudio (version see above) was used for visualization of the data.

Scripts are available on GitHub: https://github.com/tiinatalts/RSV_wgs

## RESULTS

### Detection and typing RSV-AB/hMPV/IC multiplex real-time RT-PCR

*Analytical sensitivity:* RSV-A detection and typing RT-PCR LLoD was assessed with Probit regression analysis to be 205 copies/mL (95% CI 145–405 copies/mL; 95th centile) in the range of Ct 34.71–36.41. RSV-B detection and typing RT-PCR LLoD has been assessed with Probit regression analysis to be 1173 copies/mL (95% CI 855–2,398 Copies/mL; 95th centile) in the range of Ct 35.68–37.66. Materials used for analytical sensitivity assessment: Vircell Amplirun Respiratory Syncytial Virus RNA control purified complete microbial genome (Subtype A; AY911262.1; Subtype B: AY353550.1).

*Analytical specificity:* the following respiratory viruses were tested in the assay resulting in expected undetected result: Influenza: A(H1N1)pdm09, A(H3N2), B/Victoria, B/Yamagata, A(H1N2); Avian Influenza: A(H5N1), A(H7N9), A(H9N2), A(H5N6); hPIV types 1, 2, 3, and 4, hAdV C1, hAdV A31, hRhV A1, hRhV C9, hMPVA, hMPVB, RSV-A, RSV-B, hCoV-229E, hCoV-OC43, hCoV-NL63, hCoV-HKU1, MERS-CoV, SARS CoV-2, and SARS CoV-1 Hong Kong (for further details, please see (Table S2).

*Clinical sensitivity/specificity:* based on 166 previously sequence confirmed RSV primary care positive URT clinical samples (legacy RSV WGS assay based on Kumaria, (9)) and 169 previously confirmed negative URT samples the RSV-A sensitivity was 100%, specificity 100%, accuracy 100% (95% CI 98.44%–100%); RSV-B sensitivity was 100%, specificity 100%, accuracy 100% (95% CI 98.44%–100%).

### RSV-A RSV-B WGS assays

#### Analytical sensitivity

The aim of the initial WGS analytical sensitivity experiments was to assess assay performance when sequencing directly from clinical samples that inevitably have differing viral loads. Therefore, we needed to establish a pragmatic Ct cut-off from the RT-qPCR detection/typing assay to identify which clinical samples could be run on RSV WGS with a high probability of generating >90% viral genome with acceptable depth of coverage. The criterion of greater than >90% coverage does not include a specific requirement for the coverage of G and F gene regions. To establish an RT-qPCR detection/typing Ct cut-off threshold value, we conducted an assessment of the assay in an end-to-end fashion involving all the components in the workflow to produce the final output. We used cell-cultured RSV-A and RSV-B viruses (Table 1) to ensure uniformity of input material. The results from this experiment allowed us to assess the sensitivity, reproducibility, repeatability, precision, and robustness of 11 technical replicates in differing workflow permutations (Supplementary Supporting Information Fig. S1).

The quantities of the input RSV-A and RSV-B cell cultured virus are shown in (Table S1) with corresponding RSV RT-qPCR Ct values, dPCR absolute quantitation, and pfu values. We calculated the measure of infectivity or particle to pfu ratio (P:pfu) for the material to be 347 and 366 for RSV-B and RSV-A, respectively, which is 10-fold lower than the previously reported value of 3,200 (33, 34).

**Fig 1:**
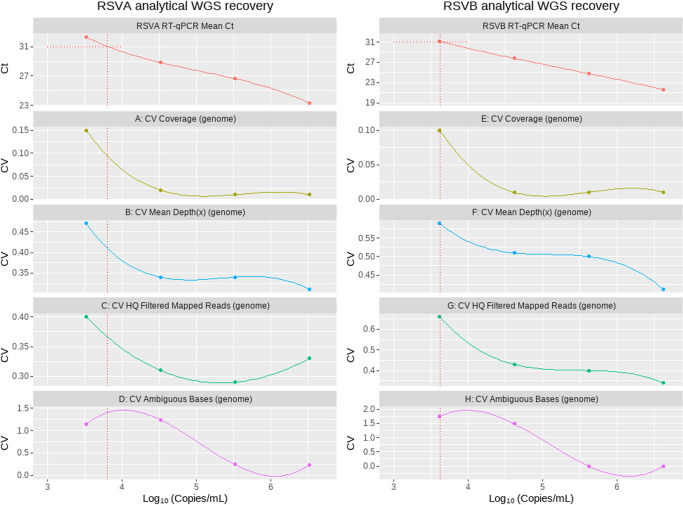
RSV-A and RSV-B lowest limit of WGS assessed by CV% across four different WGS metrics. The CV represents the ratio of the estimated standard error to the arithmetic mean. The x-axis represents log-transformed viral input concentration (copies/mL) on the linear scale. The corresponding Ct values are displayed on the top panel, followed by CV of genome coverage panels A, E; CV mean depth panels B, F; CV HQ filtered mapped reads on panels C, G; and CV of ambiguous bases on panels D, H. The dotted red line represents the Ct cut-off applied across all the panels projected onto the x-axis to the corresponding log-transformed viral concentration. The cut-off is applied at the highest acceptable level of error in the input sample observed where the CV% value change from the baseline CV% is ~10% to 20% consistently across all metrics assessed.

The resulting data were summarized as the final coefficient of variation (CV), obtained from 11 replicates from the end-to-end process (Fig. 1). The CV was presented over four different metrics: genome coverage (Fig. 1A and E), average depth of genome coverage (Fig. 1B and F), HQ mapped filtered read counts per genome (Fig. 1C and G), and ambiguous base counts per genome (Fig. 1D and H). Each CV was plotted per log-transformed input of virus concentration on a linear scale, and respective Ct values were included for comparison overview. The approximate virus input concentration was assessed per dilution using the dPCR absolute quantitation method. The obtained approximate concentration was established over three technical replicates performed on separate occasions, and the mean value derived from this data was comparable to that obtained from the RT-qPCR LLoD Probit analysis. The CV represents the ratio of the estimated standard error to the arithmetic mean. The Ct cut-off threshold was established as the level of error observed in an input sample where the CV value change from the baseline CV was ~10% to 20% consistently across metrics assessed. Using this approach, a Ct cut-off of 31 was established for both RSV-A and RSV-B detection signals of the subtyping assay at approximate copy numbers of 8 × 10^3^ copies/mL and 4 × 10^3^ copies/mL, respectively. The data show that the assay was highly sensitive and that a Ct cut-off of 31 could be used for future analysis of clinical samples.

#### Analytical specificity

Co-infection with other viruses is common, particularly in infants. To assess the analytical specificity of our assay in the context of contaminating viral RNA, the dilution series aliquots of the RSV-A and RSV-B control material were each spiked with a pool of additional respiratory pathogens, using material for the analytical sensitivity assessments above, with viruses most likely to be found in URT samples as described above. The results of RSV-A and RSV-B sequencing assays were analyzed in the same way as previous results for analytical sensitivity. The comparative CV values across the four metrics (genome coverage, average depth of genome coverage, HQ filtered mapped read counts, and content of ambiguous bases) were calculated in comparison to the mean and standard deviation of the established analytical sensitivity baseline which are presented in Fig. 1. The resulting plot (Supplementary Supporting Information Fig. S2 and S3) compares previously established baseline with the spiked material showing that CV values are mostly below the baseline CV, indicating no major effect of additional pathogens in the simulated clinical samples on the respective RSV-A and RSV-B assay performances.

Analytical specificity was assessed separately for RSV-A and RSV-B co-infection. A simulated co-infection panel was generated comprising material from the RSV-A and RSV-B RNA dilution series combined in an antiparallel direction; this allowed us to assess the possible effects at differing RSV-A and RSV-B virus input concentrations and ratios. The CV values in the co-infection studies (Supplementary Supporting Information Fig. S2 and S3) were below the baseline CV which indicates no effect of co-infection on the respective RSV-A and RSV-B assay performance, except when there is co-infection with low-level RSV-A input but high-level input of RSV-B. The CV of ambiguous bases was slightly higher than the corresponding baseline at 3.52/6.62 log copies/mL RSV-A and RSV-B. However, the corresponding Ct value for the affected dilution of RSV-A was slightly outside of the established cut-off of Ct 31 and thus unlikely to affect sequencing results in routine settings.

To assess the ability to accurately detect viral sub-populations, we generated a simulated panel that consisted of two viruses with a differential base at two chosen “Single Nucleotide Variant (SNV)” positions in the genome. The panel consisted of serially prepared samples each with different input ratios of the two selected viruses. The linearity of calling a base at an SNV position in the genome was performed using a previously described method (35). Two SNV sites per RSV type were assessed resulting in linearity plots with slope values ranging from 0.816 to 0.91 (R^2^ = 0.86 to 0.91) for RSV-A and 0.984 to 0.991 for RSV-B (R^2^ = 0.91 to 0.97). Linearity within the accepted range of 0.5–1.5 was observed in the two assessed SNV sites in the base calling frequencies (Supplementary Supporting Information Fig. S4).

WGS protocol clinical sensitivity/specificity was further supported by successful RSV sequence generation from clinical samples with co-infection. As well as the RSV alone sequences, using WGS, we obtained whole-genome or partial genome sequences from the following numbers of co-infected clinical samples: RSV and Coxsackievirus (12), RSV and Echovirus 9 (3), RSV and Enterovirus VP1 (6), RSV-B and SARS CoV-2 (2), RSV and Influenza A (8), and RSV and human adenovirus (8) [see further details of type/lineage and Accession IDs (where available) in (Table S3)]. We obtained RSV-A and RSV-B co-infection whole-genome sequences for both types for five clinical samples and sequence confirmed two triple infection samples of RSV-A, human adenovirus and Coxsackievirus B3; and RSV-A, RSV-B, and Echovirus 9 (Table S2).

#### Comparison with legacy multi-amplicon WGS protocol

We used the new RSV WGS assay to re-sequence 21 RSV-A and 16 RSV-B clinical samples that had been previously sequenced with a multi-amplicon WGS protocol based on Kumaria et al. (GISAID sequence IDs available in (Table S3). A total of three consensus sequences were produced per sample: (i) consensus obtained with the previous assay and bioinformatics pipeline, (ii) consensus obtained with the previous assay but re-analyzed with the updated bioinformatics pipeline, and (iii) consensus sequence obtained with the updated assay and updated bioinformatics pipeline. The three x 21 RSV-A consensus whole-genome sequences were trimmed at the very terminal ends of the sequences to equalize the length between all sequences. They were aligned using the EMBL Clustal Omega multiple sequence alignment tool. The percent identity matrix was used to compare the sequence similarities. Overall, the 21 previously sequenced RSV-A clinical samples had a mean percent identity of 99.9 with an SD of 0.108 (95% CI 0.948–0.999) when legacy assay and pipeline were compared to legacy assay results re-analyzed with the current pipeline; and a mean percent identity of 99.994 with an SD of 0.008 (95% CI 0.996–1.000) when legacy assay and pipeline were compared to the updated current assay analyzed with the current pipeline. Similarly, the previously sequenced 16 RSV-B clinical samples had a mean percent identity of 99.951 with an SD of 0.153 (95% CI 0.914–0.999) when legacy assay and pipeline were compared to legacy assay results re-analyzed with the current pipeline; and a mean percent identity of 99.834 with an SD of 0.243 (95% CI 0.864–0.998) when legacy assay and pipeline were compared to the updated current assay analyzed with the current pipeline (Table S4 and S5). The sequences for the validation set have previously been submitted to GISAID under the virus names in (Table S3 through S5) further including the GISAID IDs.

#### WGS recovery success rate in clinical samples

Using the established threshold, only clinical samples that were detected with Ct <31 (equivalent to 3 × 10^3^ to 7 × 10^3^ copies/mL of viral concentration) in the detection/subtyping RSV RT-qPCR assay were subject to WGS sequencing workflows.

**Fig 2:**
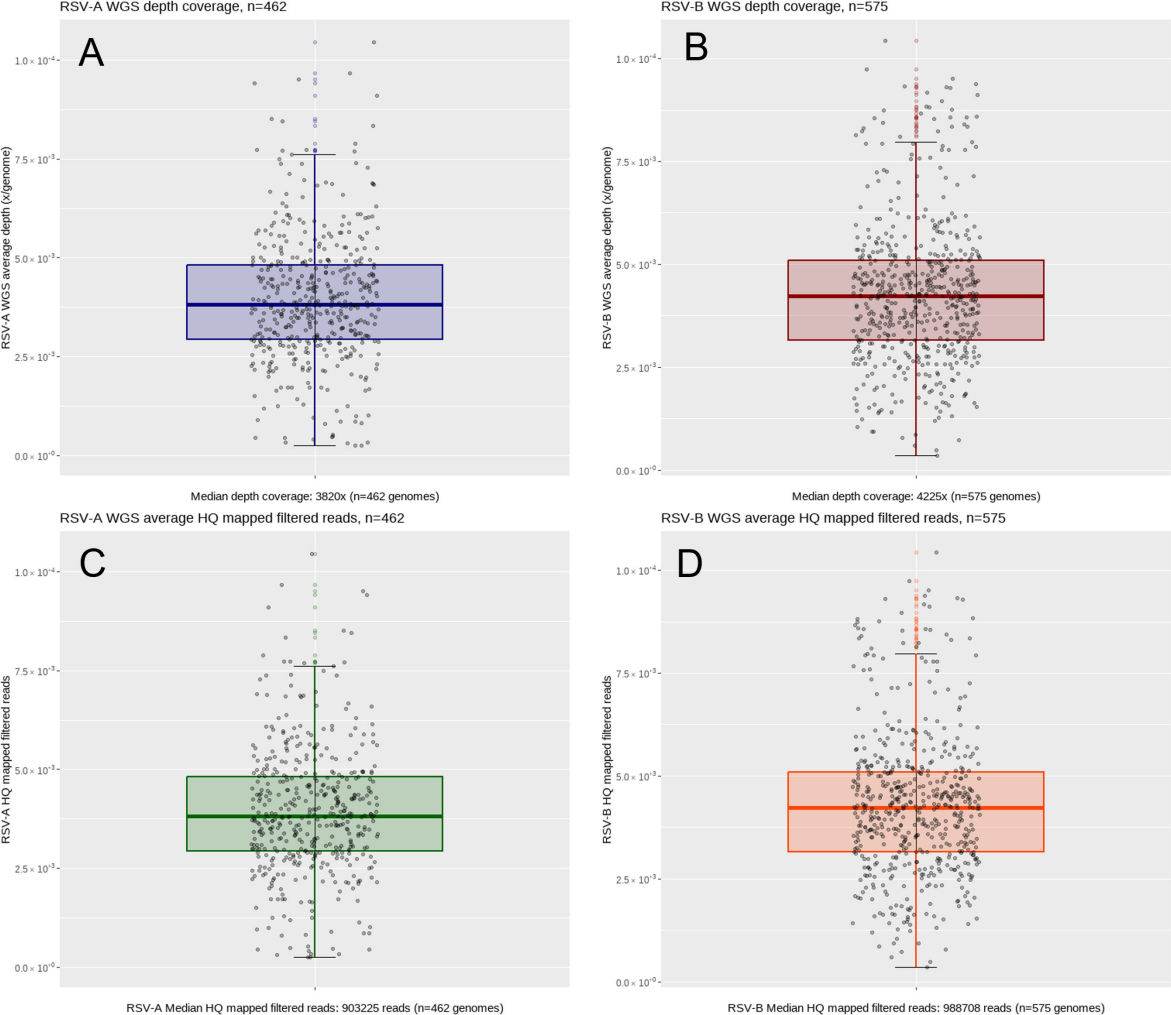
Boxplots for RSV WGS average depth of coverage and HQ mapped filtered read counts across RSV-A (panels A and C) and RSV-B (panels B and D) WGS assays. The data of the assay’s performance are analyzed over 1,037 clinical samples (RSV-A: 462; RSV-B: 575). The median depth coverage for RSV-A and RSV-B WGS assays is >4,000 reads and median HQ mapped filtered read counts were > 9×10^6^ reads.

**Fig 3:**
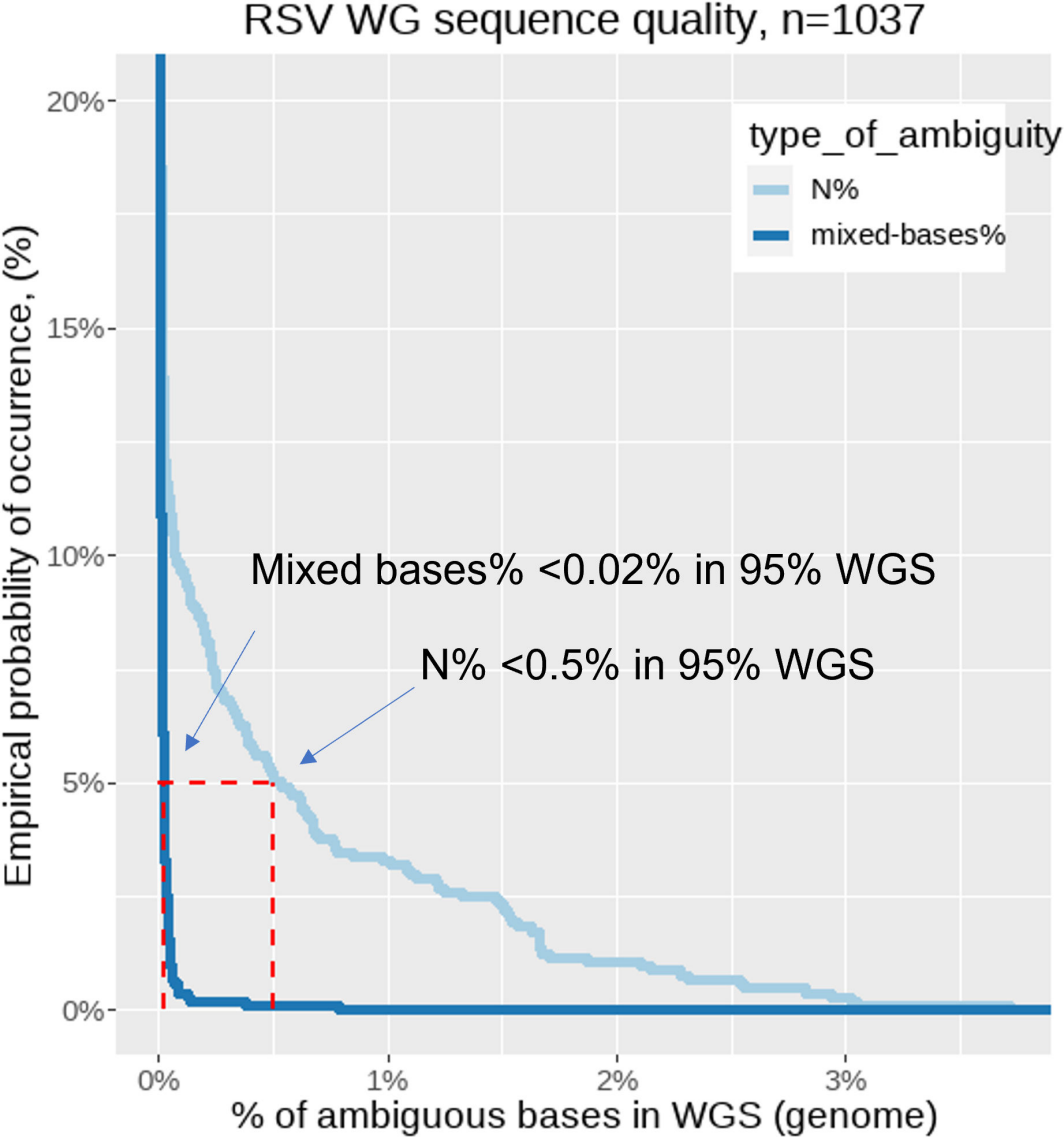
RSV WGS sequence quality when assessing the ambiguous bases. The empirical cumulative probability of occurrence y-axis is truncated for clarity of plotted data. The ambiguity stratified as N% or failed base calls and ambiguity or mixed bases. Base calling with ≥15 read depth at a position and minimum proportion to affect an ambiguity is 20%, resulting in 95% of the 1,037 genomes containing <0.02% of ambiguous bases and <0.5% of Ns or failed base calls.

A total of 1,071 RSV-positive clinical samples with a Ct <31 were included. They were collected during the time period of December 2019 to March 2023 (743 from primary care; 328 from secondary care) and subjected to WGS analysis using the 4-primer-pool, short-amplicon PCR-tiling approach. The obtained consensus whole-genome sequences with over 4% of unknown bases (“N”) were excluded from the submission to the external database (GISAID EpiRSV). In addition to the obtained coverage percentage of the consensus sequence, the following further criteria were considered: percentage of ambiguous bases in the consensus sequence, mean depth of coverage of the genome, consensus sequence length obtained, raw mapped read counts, and HQ filtered mapped read counts per whole genome. The resulting 1,037 (96.83%) successfully obtained, high-quality genome sequences were deposited to the GISAID EpiRSV database (Table S3). The 1,037 sequences have a coverage of ≥96% across the genome, with a median depth of 4,029, a median of 946,066 HQ mapped filtered reads per genome (Fig. 2). Furthermore, 95% of the 1,037 genomes contain <0.02% of ambiguous bases and <0.5% of Ns or failed base calls (Fig. 3). The resulting overall success rate is 96.83% clinical samples with a Ct <31. The newly obtained 1,008 RSV WGS sequences obtained with the assay have been deposited to the GISAID EpiRSV database (Table S3). The GISAID deposition count includes RSV-A and RSV-B co-infections of the five clinical samples mentioned above as individual submissions where whole-genome coverage was obtained for both viruses from one specimen. More details of the co-infection samples can be found in (Table S3).

**Fig 4:**
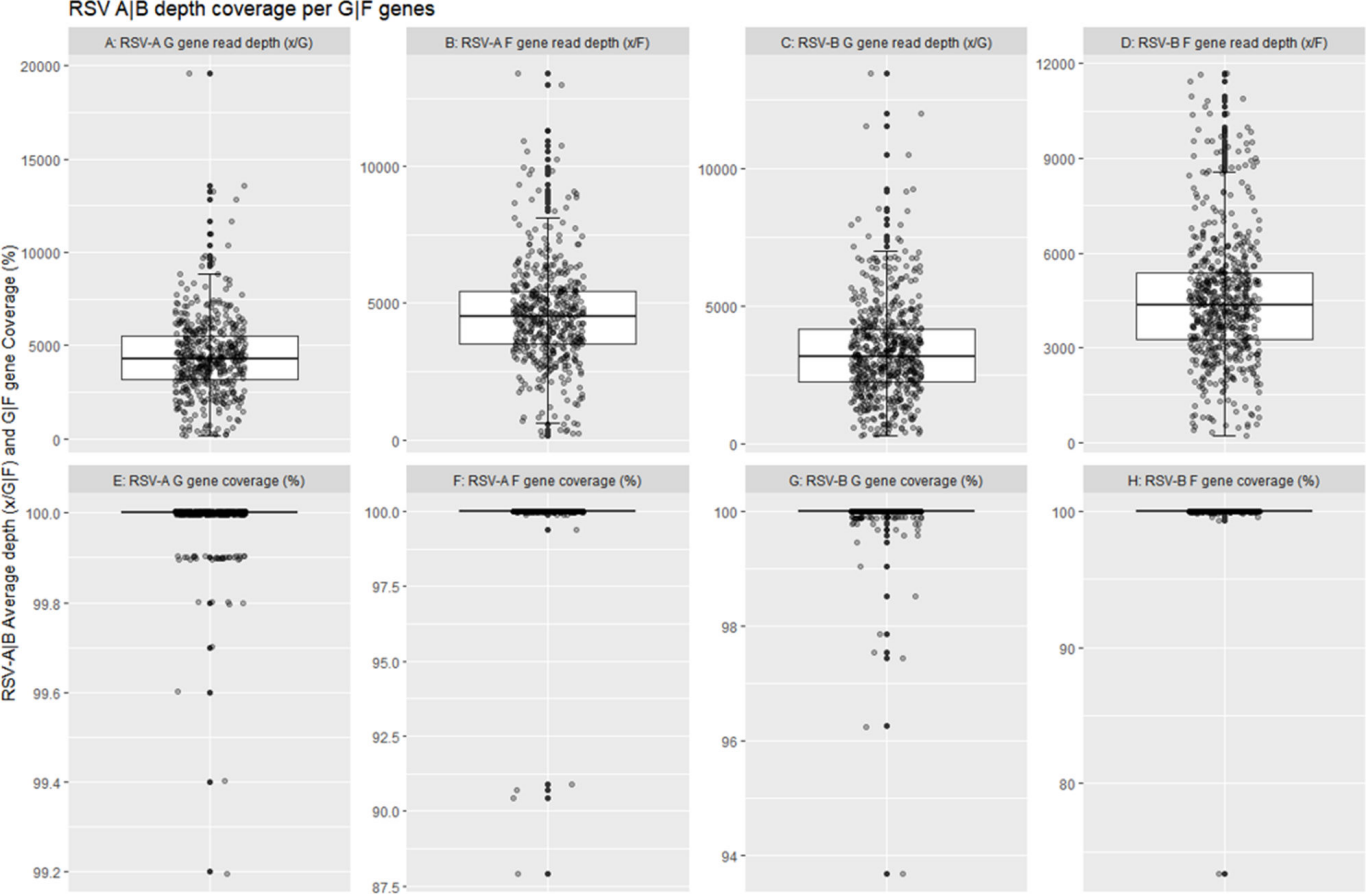
RSV-A and RSV-B F and G gene region coverage and read depth. RSV-A G gene region nucleotide sequence read depth median 4,416 reads (panel A), RSV-A G gene region coverage percentage at d20 median coverage 99.987% (panel E), *n* = 433; RSV-A F gene region nucleotide sequence read depth median 4,575 reads (panel B), RSV-A F gene region coverage percentage at d20 median coverage 99.909% (panel F), *n* = 433; RSV-B G gene region nucleotide sequence read depth median 3,354 reads (panel C), RSV-B G gene region coverage percentage at d20 median coverage 99.953% (panel G), *n* = 604; RSV-B F gene region nucleotide sequence read depth median 4,526 reads (panel D), RSV-B F gene region coverage percentage at d20 median coverage 99.946% (panel H), *n* = 604.

To assess the quality of sequences obtained from the 1,037 clinical samples for G and F genes, we analyzed the coverage and depth of the reads specifically obtained for G and F gene regions of RSV-A and RSV-B separately (Fig. 4). The nucleotide sequence median coverage obtained for RSV-A G gene region was 99.99% (Fig. 4 panel E, *n* = 433) with a median read depth of 4,416 reads (Fig. 4 panel A). Similarly, the median coverage for an RSV-A F gene region was 99.91% (Fig. 4 panel F, *n* = 433) with a median read depth of 4,575 reads (Fig. 4 panel B). RSV-B G gene region nucleotide sequence median read depth was 3,354 reads (Fig. 4 panel C, *n* = 604) with RSV-B G gene region median coverage of 99.95% (Fig. 4 panel G). RSV-B F gene region nucleotide sequence median read depth was 4,526 reads (Fig. 4 panel D, *n* = 604) with RSV-B F gene region median coverage of 99.95% (Fig. 4 panel H, *n* = 604).

#### Analysis by age

**Fig 5:**
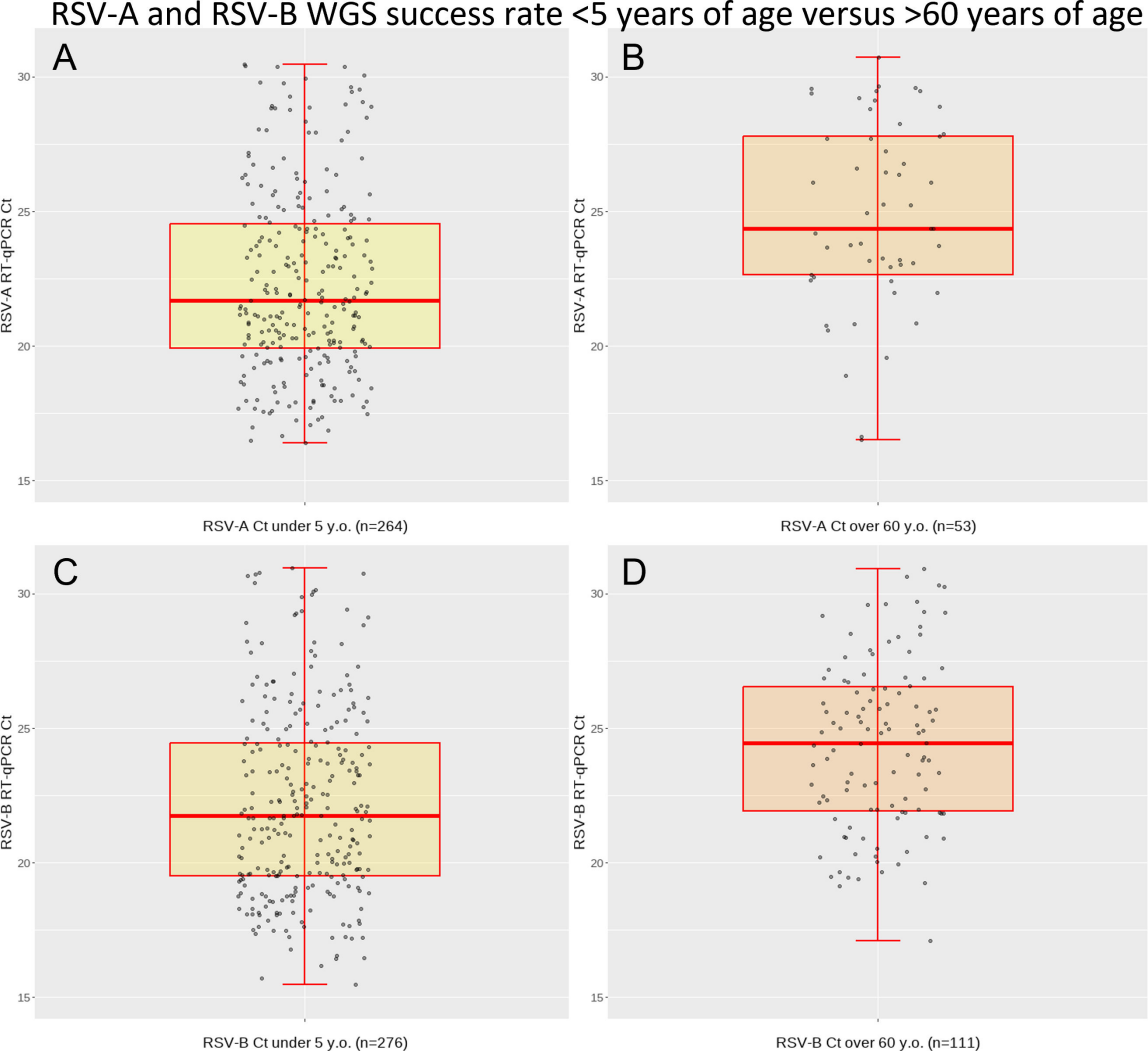
RSV WGS success rate comparison from samples of patients aged < 5 years versus >60 years. Plotted samples of successfully obtained WG sequences (Ct <31). All samples RCGP (primary care) nasopharyngeal specimens. Median Ct of <5 yo =21.76 over RSV-A and RSV-B RT-PCR assays corresponds to approximately 10^4^ copies/mL; median Ct of >60 yo =24.64 over RSV-A and RSV-B RT-PCR assays corresponds to approximately 10^3^ copies/mL. Bootstrap 95% confidence interval for the difference in median change lies between 01.950 and 03.62 (CI 95% Bootstrap *n* = 10,000). Difference between distributions by KS test: two-sided *P*(<0.0001). Copy number approximation based on detection assay LLoD Probit analysis and dPCR absolute quantitation data.

WGS recovery success rate of clinical specimens is affected by many factors including sample integrity, viral abundance in the specimen, and any inhibitors present, among many other external and internal factors. Here we looked at one possible external factor, the patient’s age (Fig. 5). All included in this comparison are primary care nasopharyngeal swab specimens with a collection date ranging from 2008 to the present (*N* = 721) irrespective of the WGS method used. As these samples were taken in a uniform manner over this period of time and stored at −80°C, samples included in the comparison yielded successful whole-genome sequences with a Ct <31 indicating good sample integrity. The samples from which a WGS was obtained were compared in digital PCR for approximate viral copy number and segregated by age (*N* = 549; <5 yo) and (*N* = 172; >65 yo) (Fig. 5).

The approximate viral copy numbers for respective median Ct values demonstrated a significant difference when comparing log-transformed approximate concentrations from children and elderly adults. The statistical difference in distributions was determined by two different methods: first, by bootstrapping with i = 5,000 and using the KS test *P*(<0.0001) as is appropriate for non-normal distributions. Both methods indicate a significant difference in the distributions of the data sets. The data indicate that samples from elderly individuals are likely to contain lower viral loads, compared with children, which may impact on sampling strategies used for genomic surveillance.

## DISCUSSION

The RSV A and B whole-genome sequencing technical approaches described in this study have been shown to have increased sensitivity when compared to previous approaches used for RSV based on Kumaria et al., proving to be appropriate for application to sequence RSV in respiratory clinical specimens. The sensitivity of the updated approach is appropriate for processes where amplification for sequencing is applied directly to nucleic acid extracted from respiratory clinical specimens, without needing any additional prior or further enrichment procedures. Together with improved throughput, this resulted in better overall cost-effectiveness and turnaround time. The success rate was 97% which is based on 1,037 whole-genome sequences obtained out of the 1,071 attempted clinical samples (including validation data) that produce whole-genome sequences with a genome coverage of ≥96%, including >99.95% coverage over G and F gene regions, and with an applied Ct cut-off of Ct31.

We have demonstrated the analytical specificity of the updated RSV WGS approach to be excellent and the sequencing quality was not affected when sequencing co-infections with other respiratory pathogens and co-infections with RSV-A and RSV-B. We have also examined the assay’s handling capabilities with the viral sub-populations in which they demonstrate a linear frequency of base calls at the SNV sites. The quality of sequence produced is essential in providing national and global genomic variation surveillance for preparedness for new interventions in monitoring antigenic escape, where a single point mutation might lead to a reduction in antibody binding effectiveness and neutralizing activity (36); or indeed in the monitoring of retaining susceptibility to neutralization by existing and new interventions (37, 38).

The updated assay design follows a previously described SARS-CoV-2 2-pool multi-primer amplicon approach (https://dx.doi.org/10.17504/protocols.io.bp2l6n26rgqe/v3). However, the RSV WGS approach differs by consisting of two complementing primer schemes that produce amplicons with slight variation in lengths—the shorter amplicons scheme adds to the sensitivity while the longer amplicons scheme accommodates diversity reducing the risk of sequence dropouts. However, the limitations of the method would be the requirement to know the subtype of the positive sample beforehand; the number of four separate amplifications per sample which might need robotics when sequencing a large number of samples. Furthermore, the current approach has been developed using short sequencing approaches (i.e., Illumina platform) and not tested on other technologies, for example, Oxford Nanopore Technologies. However, the RSV-A- and RSV-B-specific assays provide reliability and simplicity for obtaining whole-genome sequences of both RSV-A and RSV-B subtypes from co-infection samples.

In addition, we have investigated how a patient’s ages affect the RSV WGS success and have confirmed previously reported observations (39, 40, 41) showing a lower viral abundance in samples collected from elderly patients versus viral abundance levels in young children. This has implications for surveillance schemes and sampling strategies around national interventions introduced in the elderly population and emphasizes the importance of the availability of a sensitive and robust WGS assay. Despite their attractiveness, long amplicon approaches, as described by Dong and Deng (12), in our experience can lead to sample integrity issues such as sample degradation (during high-throughput handling and freeze/thaw cycles), and thus might not provide the desired robustness.

In conclusion, the current RSV WGS approach has multiple advantages over the legacy approach: sufficient genome amplification that provides increased sensitivity, cost-effectiveness, higher throughput, and turnaround time resulting in high-quality RSV-A and RSV-B whole-genome sequences. The updated approach produces sequences of high quality consistently and cost-effectively, and quality metrics match requirements for upload onto GISAID/sharing with international agencies, therefore suitable for implementation to underpin national programs and global surveillance initiatives such as that of WHO for the surveillance of RSV genomic variation (5).

## Data Availability

The complete sequences obtained from clinical specimens were deposited in the GISAID EpiRSV database (Table S3).

## SUPPLEMENTAL MATERIAL

Figure S1 spectrum.03067-23-s0001.pdf RSV WGS workflow and data analysis.

Figure S4 spectrum.03067-23-s0002.pdf SNV linearity.

Figures S2 and S3 spectrum.03067-23-s0003.pdf Analytical specificity.

Table S1 spectrum.03067-23-s0004.pdf Analytical sensitivity copy number, RT-qPCR Ct, and PFU quantities of the material used in assessment.

Table S2 spectrum.03067-23-s0005.pdf Exclusion panel.

Table S3 spectrum.03067-23-s0006.pdf GISAID ID table.

Table S4 spectrum.03067-23-s0007.pdf RSVA clinical sensitivity.

Table S5 spectrum.03067-23-s0008.pdf RSVB clinical sensitivity.

ASM does not own the copyrights to Supplemental Material that may be linked to, or accessed through, an article. The authors have granted ASM a non-exclusive, world-wide license to publish the Supplemental Material files. Please contact the corresponding author directly for reuse.
