# Supplementary material for: Robust and sensitive amplicon-based whole-genome sequencing assay of respiratory syncytial virus subtype A and B
Source: Microbiol Spectr. 2024 Feb 27;12(4):e03067-23. doi: 10.1128/spectrum.03067-23 (PMC10986592; doi:10.1128/spectrum.03067-23)
Supplement: Figure S1 — RSV WGS workflow and data analysis. [file spectrum.03067-23-s0001.pdf]

Robust and sensitive amplicon based whole genome sequencing assay of respiratory syncytial virus (RSV) subtype A and B

[authors: Tiina Talts, Lucy Mosscrop, David Williams, John S. Tregoning, Whitney Paulo, Arinder Kohli, Thomas C Williams, Katja Hoschler, Joanna Ellis, Simon de Lusignan, Maria Zambon]

[Institution: UKHSA; ICL; The University of Edinburgh; University of Oxford]

Supplementary Supporting Information – Figure S1

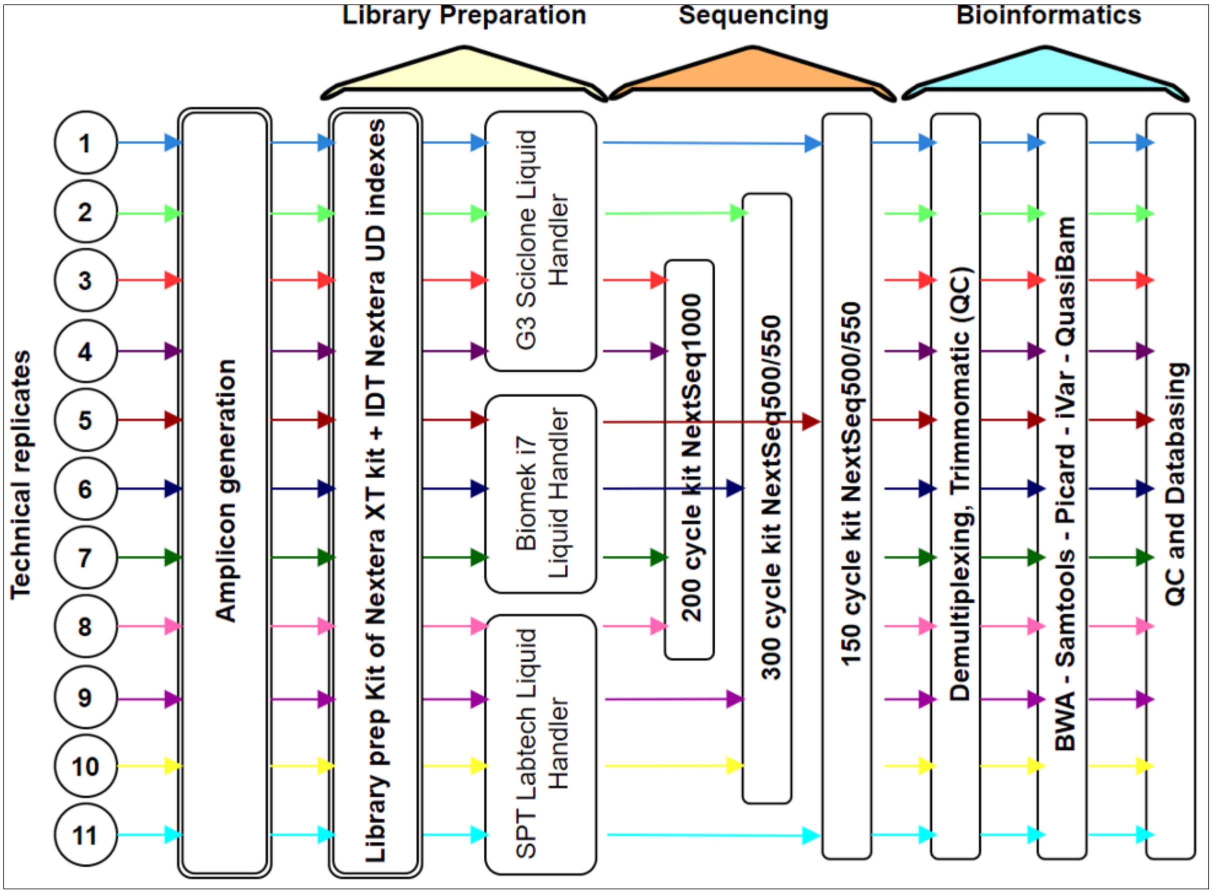

Figure S1 **RSV WGS workflow and data analysis**. Technical replicates represent the experimental design for the analytical assessment. Pre-library preparation and bioinformatics steps are identical between the replicates. Differences are depicted in library preparation and sequencing steps.
