# Supplementary material for: Robust and sensitive amplicon-based whole-genome sequencing assay of respiratory syncytial virus subtype A and B
Source: Microbiol Spectr. 2024 Feb 27;12(4):e03067-23. doi: 10.1128/spectrum.03067-23 (PMC10986592; doi:10.1128/spectrum.03067-23)
Supplement: Figure S4 — SNV linearity. [file spectrum.03067-23-s0002.pdf]

Robust and sensitive amplicon based whole genome sequencing assay of respiratory syncytial virus (RSV) subtype A and B

[authors: Tiina Talts, Lucy Mosscrop, David Williams, John S. Tregoning, Whitney Paulo, Arinder Kohli, Thomas C Williams, Katja Hoschler, Joanna Ellis, Simon de Lusignan, Maria Zambon]

Supplementary Supporting Information – Figure S4: RSV WGS assessment of linearity of sequencing minority variant base calls at subspecies level

Figure S4: RSV WGS assessment of linearity sequencing minority variant base calls at subspecies level. Two separate sites were assessed per each type: RSV-A sites 5612 A, G (panels: A, B) and 7980 A, G (panels C, D) (positions corresponding to GenBank acc. no.:NC_038235.1); and RSV-B sites: 5800 A, G (panels E, F) and 7479 T, C (panels G, H) (positions corresponding to GenBank acc. no.:NC_001781.1). Empirical estimation was conducted for base call frequencies of two virus variants (y - axis: ln variant frequency) at differing input ratios (x – axis: ln variant virus input ratio). The slope of natural log of frequency as a function of the natural log of the variant fraction is used to assess the success of the linearity. Data is based on mean values of 6 replicates performed on two separate occasions. A slope value within the range of 0.5 to 1.5 would indicate accuracy level of the entire sequencing end-to-end workflow.

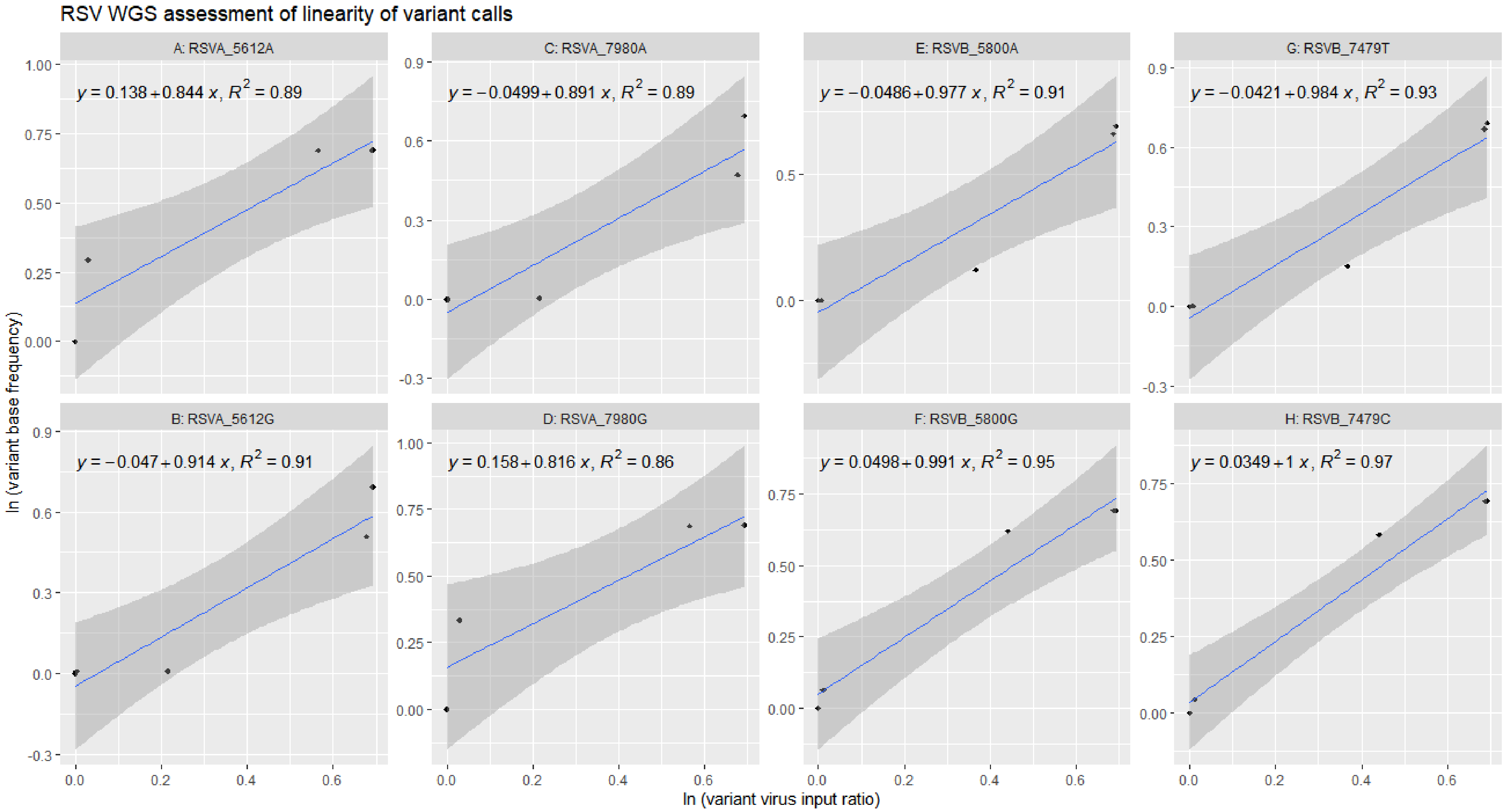

RSV WGS assessment of linearity of variant calls
A: RSVA_5612A
y = 0.138 + 0.844 x, R2 = 0.89
C: RSVA_7980A
y = −0.0499 + 0.891 x, R2 = 0.89
E: RSVB_5800A
y = −0.0486 + 0.977 x, R2 = 0.91
G: RSVB_7479T
y = −0.0421 + 0.984 x, R2 = 0.93
B: RSVA_5612G
y = −0.047 + 0.914 x, R2 = 0.91
D: RSVA_7980G
y = 0.158 + 0.816 x, R2 = 0.86
F: RSVB_5800G
y = 0.0498 + 0.991 x, R2 = 0.95
H: RSVB_7479C
y = 0.0349 + 1 x, R2 = 0.97
ln (variant base frequency)
ln (variant virus input ratio)
