# Supplementary material for: Robust and sensitive amplicon-based whole-genome sequencing assay of respiratory syncytial virus subtype A and B
Source: Microbiol Spectr. 2024 Feb 27;12(4):e03067-23. doi: 10.1128/spectrum.03067-23 (PMC10986592; doi:10.1128/spectrum.03067-23)
Supplement: Figures S2 and S3 — Analytical specificity. [file spectrum.03067-23-s0003.pdf]

**Robust and sensitive amplicon based whole genome sequencing assay of respiratory syncytial virus (RSV) subtype A and B**

[authors: Tiina Talts, Lucy Mosscrop, David Williams, John S. Tregoning, Whitney Paulo, Arinder Kohli, Thomas C Williams, Katja Hoschler, Joanna Ellis, Simon de Lusignan, Maria Zambon]

[Institution: UKHSA; ICL; The University of Edinburgh; University of Oxford]

**Supplementary Supporting Information – Figure S2 and Figure S3**

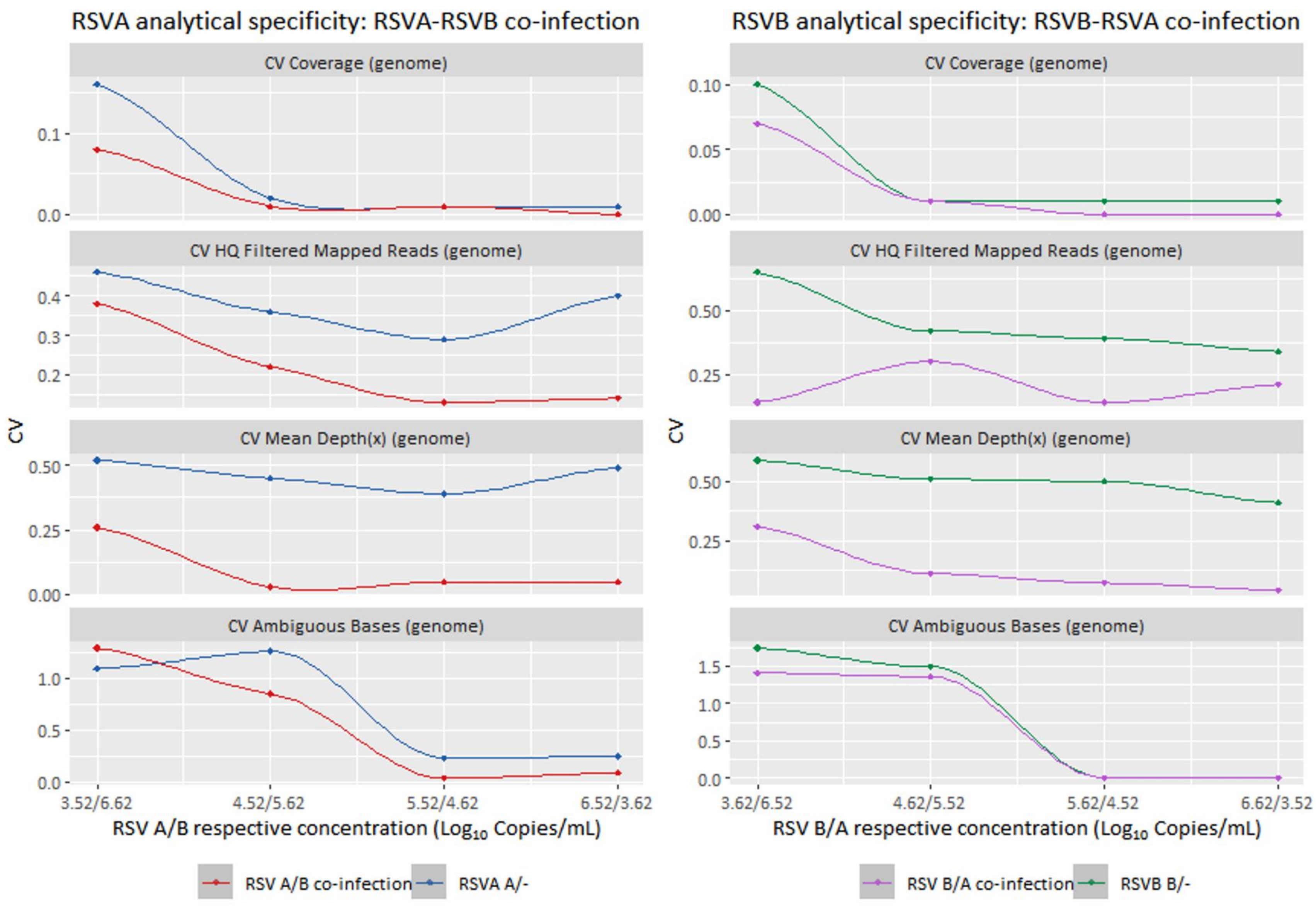


*Figure S2* RSV-A and RSV-B co-infection analytical specificity. Simulated co-infection concentrations of the input viruses on x-axis, plotted with the corresponding non-coinfection data for comparison that has the same concentration of input but without the added co-infection virus. Coefficient of variation (CV) of coverage, HQ filtered mapped read counts, genome mean depth and ambiguous bases on y-axis. Comparative CV calculated in comparison to the mean and standard deviation of the baseline (main Figure 2) (non-coinfection RSV-A and RSV-B, in blue and green, respectively). The co-infected CV values are below the baseline CV which confirms no effect of co-infection on the respective RSV-A and RSV-B assay performances. Except for the RSV-A assay where the quality can be seen to be affected at co-infection with low level RSV-A input but high level input of RSV-B: CV Ambiguous bases slightly higher than the corresponding baseline at 3.52/6.62 Log copies/mL RSV-A and RSV-B, respectively.

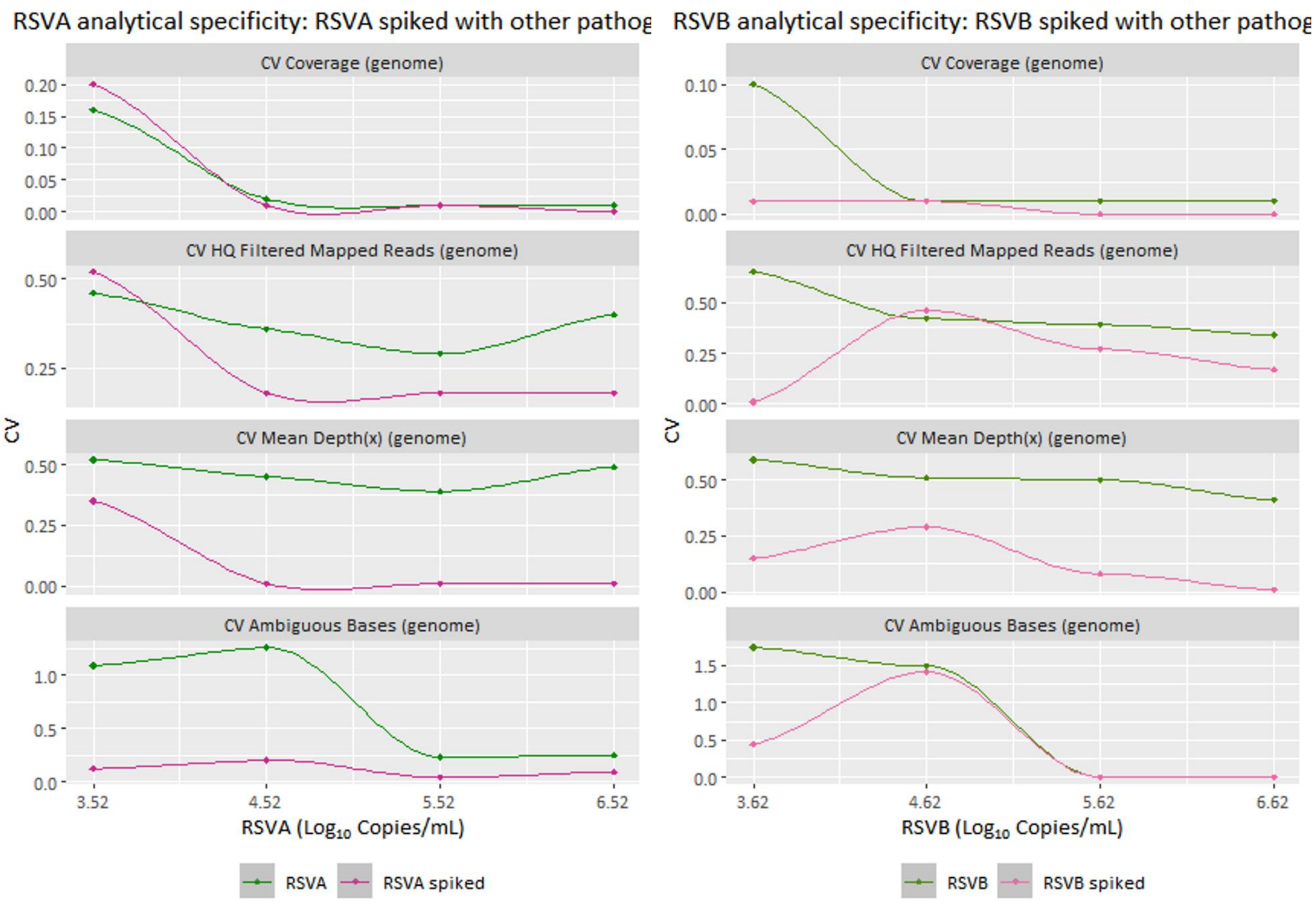


*Figure S3* RSV-A and RSV-B analytical specificity when baseline dilution series spiked with a pool of additional respiratory pathogens that are likely to be found in the URT samples. Concentrations of the spiked input virus on x-axis, plotted with the corresponding non-spiked data for comparison that has the same concentration of input but without the added spiked pool of additional respiratory pathogens. Coefficient of variation (CV) of coverage, HQ filtered mapped read counts, genome mean depth and ambiguous bases on y-axis. Comparative CV calculated in comparison to the mean and standard deviation of the baseline (main Figure 2) (non-spiked RSV-A and RSV-B, in green). The spiked dataset CV values are mostly below the baseline CV which confirms no effect of spiking on the respective RSV-A and RSV-B assay performances.
