# Supplementary material for: Robust and sensitive amplicon-based whole-genome sequencing assay of respiratory syncytial virus subtype A and B
Source: Microbiol Spectr. 2024 Feb 27;12(4):e03067-23. doi: 10.1128/spectrum.03067-23 (PMC10986592; doi:10.1128/spectrum.03067-23)
Supplement: Table S1 — Analytical sensitivity copy number, RT-qPCR Ct, and PFU quantities of the material used in assessment. [file spectrum.03067-23-s0004.pdf]

S1 Table 1: Analytical sensitivity copy number, RT-qPCR Ct and plaque forming units quantities of the material used in assessment

Target RNA and dilution (ten-fold)	Target	RSVA RT-qPCR[a] (Mean Ct)	RSVB RT-qPCR[a] (Mean Ct)	Mean Concentration of sample, (dPCR[β]; Copies/µL)	Concentration of sample, (dPCR[β]; Copies/mL)	Calculated from RSV plaque assay[γ] results performed on stock virus, (Pfu/mL)	Fold difference (Copies/Pfu i.e. particle/Pfu (P:PFU))
**RSVA (hRSV/A/England/174820313/2017; EPI_ISL_732346)[δ]**							
RSVA_10^-2	RSVA RNA	23.33	*undetected*	3292	3292000	9000	**366**
RSVA_10^-3	RSVA RNA	26.64	*undetected*	329	329200	900	
RSVA_10^-4	RSVA RNA	28.85	*undetected*	33	32920	90	
RSVA_10^-5	RSVA RNA	32.35	*undetected*	3	3292	9	
**RSVB (hRSV/B/England/174880237/2017; EPI_ISL_732349)[δ]**							
RSVB_10^-3	RSVB RNA	*undetected*	21.63	4167	4167000	12000	**347**
RSVB_10^-4	RSVB RNA	*undetected*	24.79	417	416700	1200	
RSVB_10^-5	RSVB RNA	*undetected*	27.78	42	41670	120	
RSVB_10^-6	RSVB RNA	*undetected*	31.10	4	4167	12	

[a] RSVA/B detection/typing real-time multiplex RT-qPCR

[β] Absolute quantitation by digital singleplex PCR

[γ] RSV virus titre plaque forming units by immunostain method

[δ] GISAID EpiRSV virus name and ID
