# Supplementary material for: Robust and sensitive amplicon-based whole-genome sequencing assay of respiratory syncytial virus subtype A and B
Source: Microbiol Spectr. 2024 Feb 27;12(4):e03067-23. doi: 10.1128/spectrum.03067-23 (PMC10986592; doi:10.1128/spectrum.03067-23)
Supplement: Table S2 — Exclusion panel. [file spectrum.03067-23-s0005.pdf]

Robust and sensitive amplicon based whole genome sequencing assay of respiratory syncytial virus (RSV) subtype A and B

[authors: Tiina Talts, Lucy Mosscrop, David Williams, John S. Tregoning, Whitney Paulo, Arinder Kohli, Thomas C Williams, Katja Hoschler, Joanna Ellis, Simon de Lusignan, Maria Zambon]

Supplementary Supporting Information – Table S2

Virus	Strain	Source	GenBank
Influenza A(H1N1)pdm09	A/California/7/2009 H1N1	NIBSC Potters Bar, England	NC_026433.1
Influenza A(H3N2)	A/Moscow/10/1999 H3N2	Provided by WHO CC London, through WHO global influenza network	CY121373.1
Influenza B/Victoria	B/Brisbane/60/2008	Provided by WHO CC London, through WHO global influenza network	CY073894
Influenza B/Yamagata	B/Yamagata-lineage B/Brisbane/3/2007	Provided by WHO CC London, through WHO global influenza network	EU124250
Influenza A(H1N2)	A/Swine/438207/94	APHA	KR700601.1
Avian Influenza A(H5N3)	A H5N3 A/Duck/ Singapore-Q/F119-3/1997	A H5N3 A/Duck/Singapore/97 from Director of Primary Production, Veterinary Laboratory Branch, Central Veterinary Laboratory, Singapore	GU052802.1
Avian Influenza A(H7N9)	A H7N9 NIBRG-268 A/Anhui/1/2013	A H7N9 NIBRG-268 A/Anhui/1/2013 from NIBSC Potters Bar, England	KX530497.1
Avian Influenza A(H9N2)	A H9N2 A/Quail/HK/G1/1997	A H9N2 A/Quail/HongKong/G1/97 from The University of Hong Kong, Queen Mary Hospital, Hong Kong.	KY785896
Avian Influenza A(H5N6)	A/Mute_Swan/England2017	APHA	KJ938658
hPIV type 1	C35	ATCC	JQ901971.1
hPIV type 2	Greer	ATCC	AF533012.1
hPIV type 3	C243	ATCC	NC_075446
hPIV type 4	M25	ATCC	NC_021928
hAdV C1	C1	NCPV, ECACC	AC_000017
hAdV A31	A31	NCPV, ECACC	MG872324
hRhV A1	1A	ATCC	FJ445111.1
hRhV C9	9C	ATCC	GQ223228.1
hMPVA	A	RNA, ATCC	OL794481.1
hMPVB	B	RNA, ATCC	OL794482.1
RSV-A	Long	NCPV, ECACC	AY911262.1
RSV-B	9320	ATCC	AY353550.1
hCoV-229E	229E	NCPV, ECACC	NC_002645
hCoV-OC43	OC43	NCPV, ECACC	NC_006213.1
hCoV-NL63	NL63	NCPV, ECACC	NC_005831
hCoV-HKU1	HKU1	RNA, ATCC	NC_006577
MERS-CoV	EMC SA1	HCoV EMC SA1 - from Dr. Fouchier at the Department of Viroscience, Erasmus Medical Center, Dr. Molewaterplein 50, 3015GE Rotterdam, the Netherlands	NC_019843.3
SARS CoV-2	Wuhan-Hu-1	NCPV, ECACC	NC_045512.2
SARS CoV-1 Hong Kong	HKU	NCPV, ECACC	DQ022305.2
