# Supplementary material for: Robust and sensitive amplicon-based whole-genome sequencing assay of respiratory syncytial virus subtype A and B
Source: Microbiol Spectr. 2024 Feb 27;12(4):e03067-23. doi: 10.1128/spectrum.03067-23 (PMC10986592; doi:10.1128/spectrum.03067-23)
Supplement: Table S3 — GISAID ID table. [file spectrum.03067-23-s0006.pdf]

Robust and sensitive amplicon based whole genome sequencing assay of respiratory syncytial virus (RSV) subtype A and B

[authors: Tiina Talts, Lucy Mosscrop, David Williams, John S. Tregoning, Whitney Paulo, Arinder Kohli, Thomas C Williams, Katja Hoschler, Joanna Ellis, Simon de Lusignan, Maria Zambon]

[Institution: UKHSA; ICL; The University of Edinburgh; University of Oxford]

Supplementary Supporting Information – Table S3

Table S3 The following 1008 RSV whole genome sequences obtained were deposited to GISAID EpiRSV database. Data provided for GISAID ID, GISAID virus name, virus collection date, source of sample and sequence confirmed co-infection information

GISAID_Accession_ID	GISAID_Virus_Name	Virus Collection Date	Source of Sample	Sequence confirmed co-infection comment (GISAID ID where available)
EPI_ISL_16609707	hRSV/A/England/213691286/2021	2021-09-10	Community	
EPI_ISL_16609708	hRSV/B/England/222580184/2022	2022-06-23	Primary care	
EPI_ISL_16609709	hRSV/B/England/222580185/2022	2022-06-23	Primary care	
EPI_ISL_16609710	hRSV/A/England/223560340/2022	2022-08-30	Primary care	
EPI_ISL_16609711	hRSV/B/England/224340613/2022	2022-10-21	Community	
EPI_ISL_16609712	hRSV/B/England/224460353/2022	2022-11-01	Community	hAdV-C5: sequence unpublished
EPI_ISL_16609713	hRSV/B/England/224780617/2022	2022-11-23	Primary care	IAV(H1N1): 6B.1A.5a.2a: 370N, 260E: EPI_ISL_16616058
EPI_ISL_16609714	hRSV/B/England/224800360/2022	2022-11-25	Primary care	IAV(H3N2): 3C.2a1b.2a.2: EPI_ISL_16616061
EPI_ISL_16609715	hRSV/B/England/224800365/2022	2022-11-23	Primary care	IAV(H3N2): 3C.2a1b.2a.2: EPI_ISL_16616062
EPI_ISL_16609716	hRSV/B/England/224880689/2022	2022-11-21	Community	
EPI_ISL_16609717	hRSV/B/England/224900410/2022	2022-11-29	Primary care	
EPI_ISL_16609718	hRSV/B/England/224900423/2022	2022-11-28	Primary care	Coxsackievirus B1: sequence unpublished
EPI_ISL_16609719	hRSV/B/England/224900428/2022	2022-11-29	Primary care	
EPI_ISL_16609720	hRSV/B/England/224900436/2022	2022-12-01	Primary care	
EPI_ISL_16609721	hRSV/B/England/224900454/2022	2022-11-29	Primary care	
EPI_ISL_16609722	hRSV/B/England/224900574/2022	2022-12-01	Primary care	
EPI_ISL_16609723	hRSV/B/England/224900745/2022	2022-12-02	Primary care	
EPI_ISL_16609724	hRSV/B/England/224900871/2022	2022-11-30	Community	
EPI_ISL_16609725	hRSV/B/England/224920354/2022	2022-12-01	Primary care	
EPI_ISL_16609726	hRSV/B/England/224920356/2022	2022-11-30	Primary care	
EPI_ISL_16609727	hRSV/B/England/224920370/2022	2022-11-30	Primary care	
EPI_ISL_16609728	hRSV/B/England/224920398/2022	2022-12-01	Primary care	
EPI_ISL_16609729	hRSV/B/England/224920562/2022	2022-12-02	Community	
EPI_ISL_16609730	hRSV/B/England/224940163/2022	2022-12-05	Primary care	
EPI_ISL_16609731	hRSV/A/England/224940178/2022	2022-12-02	Primary care	
EPI_ISL_16609732	hRSV/B/England/224940185/2022	2022-12-05	Primary care	
EPI_ISL_16609733	hRSV/A/England/224940212/2022	2022-12-02	Primary care	
EPI_ISL_16609734	hRSV/A/England/224940215/2022	2022-12-02	Primary care	
EPI_ISL_16609735	hRSV/B/England/224940329/2022	2022-12-04	Community	
EPI_ISL_16609736	hRSV/B/England/224960396/2022	2022-12-05	Primary care	Coxsackievirus A9: sequence unpublished
EPI_ISL_16609737	hRSV/B/England/224960404/2022	2022-12-05	Primary care	

GISAID_Accession_ID	GISAID_Virus_Name	Virus Collection Date	Source of Sample	Sequence confirmed co-infection comment (GISAID ID where available)
EPI_ISL_16609738	hRSV/B/England/224960409/2022	2022-12-07	Primary care	
EPI_ISL_16609739	hRSV/B/England/224960430/2022	2022-12-06	Primary care	
EPI_ISL_16609740	hRSV/B/England/224960703/2022	2022-12-07	Primary care	
EPI_ISL_16609741	hRSV/A/England/224960709/2022	2022-12-05	Primary care	
EPI_ISL_16609742	hRSV/A/England/224960710/2022	2022-12-05	Primary care	
EPI_ISL_16609743	hRSV/B/England/224960806/2022	2022-12-03	Community	
EPI_ISL_16609744	hRSV/B/England/225000272/2022	2022-12-12	Primary care	
EPI_ISL_16609745	hRSV/B/England/225000276/2022	2022-12-07	Primary care	
EPI_ISL_16609746	hRSV/A/England/225000277/2022	2022-12-06	Primary care	
EPI_ISL_16609747	hRSV/A/England/225000278/2022	2022-12-06	Primary care	
EPI_ISL_16609748	hRSV/B/England/225000282/2022	2022-12-06	Primary care	
EPI_ISL_16609749	hRSV/A/England/225000284/2022	2022-12-06	Primary care	
EPI_ISL_16609750	hRSV/A/England/225000316/2022	2022-12-06	Primary care	
EPI_ISL_16609751	hRSV/B/England/225000318/2022	2022-11-30	Primary care	
EPI_ISL_16609752	hRSV/A/England/225000326/2022	2022-12-06	Primary care	IAV(H1N1): 6B.1A.5a.2a: 370N, 260E: sequence unpublished
EPI_ISL_16609753	hRSV/B/England/225000341/2022	2022-12-07	Primary care	
EPI_ISL_16609754	hRSV/A/England/225000763/2022	2022-12-05	Primary care	
EPI_ISL_16609755	hRSV/A/England/225000794/2022	2022-12-05	Primary care	
EPI_ISL_16609756	hRSV/B/England/225000805/2022	2022-12-06	Primary care	
EPI_ISL_16609757	hRSV/B/England/225000839/2022	2022-12-06	Community	
EPI_ISL_16609758	hRSV/A/England/225000847/2022	2022-12-07	Community	
EPI_ISL_16609759	hRSV/B/England/225000914/2022	2022-12-08	Community	
EPI_ISL_16609760	hRSV/A/England/225020459/2022	2022-12-09	Primary care	
EPI_ISL_16609761	hRSV/B/England/225020461/2022	2022-12-06	Primary care	
EPI_ISL_16609762	hRSV/B/England/225020464/2022	2022-12-08	Primary care	
EPI_ISL_16609763	hRSV/B/England/225020471/2022	2022-12-08	Primary care	
EPI_ISL_16609764	hRSV/A/England/225020480/2022	2022-12-07	Primary care	
EPI_ISL_16609765	hRSV/B/England/225020493/2022	2022-12-06	Primary care	
EPI_ISL_16609766	hRSV/A/England/225020496/2022	2022-12-08	Primary care	
EPI_ISL_16609767	hRSV/B/England/225020827/2022	2022-12-09	Primary care	
EPI_ISL_16609768	hRSV/B/England/225020828/2022	2022-12-08	Primary care	
EPI_ISL_16609769	hRSV/B/England/225020836/2022	2022-12-08	Primary care	
EPI_ISL_16672797	hRSV/A/England/224020838/2021	2021-09-10	Primary care	
EPI_ISL_16672827	hRSV/A/England/224580782/2022	2022-11-23	Community	
EPI_ISL_16672828	hRSV/A/England/224600226/2022	2022-11-23	Primary care	
EPI_ISL_16672829	hRSV/A/England/224600236/2022	2022-11-24	Primary care	
EPI_ISL_16672851	hRSV/A/England/222800243/2022	2022-07-07	Community	
EPI_ISL_16672852	hRSV/A/England/224020838/2022	2022-09-30	Community	
EPI_ISL_16672853	hRSV/A/England/224080661/2022	2022-10-06	Community	
EPI_ISL_16672854	hRSV/B/England/224360277/2022	2022-10-24	Community	
EPI_ISL_16672855	hRSV/B/England/224360284/2022	2022-10-15	Community	
EPI_ISL_16672857	hRSV/B/England/224360289/2022	2022-10-26	Community	
EPI_ISL_16672858	hRSV/B/England/224360332/2022	2022-10-24	Community	
EPI_ISL_16672859	hRSV/B/England/224380385/2022	2022-10-24	Community	
EPI_ISL_16672860	hRSV/B/England/224380389/2022	2022-10-25	Community	

GISAID_Accession_ID	GISAID_Virus_Name	Virus Collection Date	Source of Sample	Sequence confirmed co-infection comment (GISAID ID where available)
EPI_ISL_16672861	hRSV/B/England/224380411/2022	2022-10-25	Community	
EPI_ISL_16672862	hRSV/B/England/224380431/2022	2022-10-25	Community	
EPI_ISL_16672863	hRSV/B/England/224380437/2022	2022-10-26	Community	
EPI_ISL_16672864	hRSV/B/England/224400209/2022	2022-10-27	Community	
EPI_ISL_16672865	hRSV/B/England/224400226/2022	2022-10-25	Community	SARS CoV-2: V-22JUL-01 PROBABLE Omicron BA.2.75: sequence unpublished
EPI_ISL_16672866	hRSV/B/England/224400227/2022	2022-10-27	Community	
EPI_ISL_16672867	hRSV/B/England/224400244/2022	2022-10-28	Community	
EPI_ISL_16672868	hRSV/B/England/224400363/2022	2022-10-26	Community	
EPI_ISL_16672869	hRSV/B/England/224400372/2022	2022-10-27	Community	
EPI_ISL_16672870	hRSV/B/England/224440421/2022	2022-10-31	Community	
EPI_ISL_16672871	hRSV/B/England/224480229/2022	2022-11-02	Community	
EPI_ISL_16672872	hRSV/B/England/224480247/2022	2022-11-03	Community	
EPI_ISL_16672873	hRSV/B/England/224480249/2022	2022-11-02	Community	
EPI_ISL_16672874	hRSV/B/England/224500197/2022	2022-11-04	Community	
EPI_ISL_16672875	hRSV/B/England/224500202/2022	2022-11-03	Community	
EPI_ISL_16672876	hRSV/B/England/224500224/2022	2022-11-03	Community	
EPI_ISL_16672877	hRSV/B/England/224500233/2022	2022-11-04	Community	
EPI_ISL_16672878	hRSV/B/England/224500237/2022	2022-11-04	Community	
EPI_ISL_16672879	hRSV/B/England/224500283/2022	2022-11-03	Community	
EPI_ISL_16672880	hRSV/B/England/224500788/2022	2022-11-03	Community	
EPI_ISL_16672881	hRSV/B/England/224520233/2022	2022-11-07	Community	
EPI_ISL_16672882	hRSV/A/England/224580771/2022	2022-11-10	Community	
EPI_ISL_16672883	hRSV/B/England/224580782/2022	2022-11-09	Community	
EPI_ISL_16672884	hRSV/B/England/224600226/2022	2022-11-10	Community	
EPI_ISL_16672885	hRSV/B/England/224600236/2022	2022-11-10	Community	
EPI_ISL_16672886	hRSV/B/England/224600238/2022	2022-11-10	Community	
EPI_ISL_16672887	hRSV/A/England/224600401/2022	2022-11-11	Community	
EPI_ISL_16672888	hRSV/B/England/224600404/2022	2022-11-11	Community	
EPI_ISL_16672889	hRSV/A/England/224600407/2022	2022-11-11	Community	
EPI_ISL_16672890	hRSV/B/England/224600409/2022	2022-11-11	Community	
EPI_ISL_16672891	hRSV/B/England/224600410/2022	2022-11-11	Community	
EPI_ISL_16672892	hRSV/A/England/224600412/2022	2022-11-11	Community	
EPI_ISL_16672893	hRSV/A/England/224620385/2022	2022-11-14	Community	hAdV-C2: sequence unpublished; Coxsackievirus B3: sequence unpublished
EPI_ISL_16672894	hRSV/A/England/224800374/2022	2022-11-22	Community	
EPI_ISL_16672895	hRSV/A/England/224800397/2022	2022-11-22	Community	
EPI_ISL_16672896	hRSV/A/England/224800657/2022	2022-11-23	Community	
EPI_ISL_16672897	hRSV/A/England/224820378/2022	2022-11-28	Community	
EPI_ISL_16672898	hRSV/A/England/224820398/2022	2022-11-25	Community	
EPI_ISL_16672899	hRSV/A/England/224820420/2022	2022-11-24	Community	Coxsackievirus A6: sequence unpublished
EPI_ISL_16672900	hRSV/A/England/224820423/2022	2022-11-25	Community	
EPI_ISL_16672901	hRSV/A/England/224820425/2022	2022-11-23	Community	
EPI_ISL_16672902	hRSV/A/England/224820429/2022	2022-11-23	Community	
EPI_ISL_16672903	hRSV/A/England/224860828/2022	2022-11-24	Community	
EPI_ISL_16672917	hRSV/B/England/224120266/2022	2022-10-10	Primary care	

GISAID_Accession_ID	GISAID_Virus_Name	Virus Collection Date	Source of Sample	Sequence confirmed co-infection comment (GISAID ID where available)
EPI_ISL_16672918	hRSV/B/England/224120267/2022	2022-09-29	Primary care	
EPI_ISL_16672919	hRSV/A/England/224220342/2022	2022-10-12	Primary care	
EPI_ISL_16672920	hRSV/A/England/224220860/2022	2022-10-17	Primary care	
EPI_ISL_16672921	hRSV/B/England/224240307/2022	2022-10-18	Primary care	
EPI_ISL_16672922	hRSV/B/England/224240308/2022	2022-09-30	Primary care	
EPI_ISL_16672923	hRSV/B/England/224240315/2022	2022-10-17	Primary care	
EPI_ISL_16672924	hRSV/B/England/224240328/2022	2022-10-17	Primary care	
EPI_ISL_16672925	hRSV/B/England/224240600/2022	2022-10-17	Community	
EPI_ISL_16672926	hRSV/A/England/224280259/2022	2022-10-19	Primary care	
EPI_ISL_16672927	hRSV/A/England/224280275/2022	2022-10-18	Community	
EPI_ISL_16672928	hRSV/A/England/224280276/2022	2022-10-18	Community	
EPI_ISL_16672929	hRSV/B/England/224280280/2022	2022-10-18	Community	Enterovirus VP1 Genotype-HRV-C15: sequence unpublished
EPI_ISL_16672930	hRSV/A/England/224280288/2022	2022-10-18	Community	
EPI_ISL_16672931	hRSV/A/England/224300278/2022	2022-10-19	Community	
EPI_ISL_16672932	hRSV/B/England/224300286/2022	2022-10-19	Primary care	
EPI_ISL_16672933	hRSV/B/England/224300287/2022	2022-10-19	Primary care	
EPI_ISL_16672934	hRSV/A/England/224300294/2022	2022-10-21	Primary care	
EPI_ISL_16672935	hRSV/B/England/224300296/2022	2022-10-19	Primary care	
EPI_ISL_16672936	hRSV/B/England/224300298/2022	2022-10-21	Primary care	
EPI_ISL_16672937	hRSV/B/England/224300348/2022	2022-10-19	Primary care	
EPI_ISL_16672938	hRSV/A/England/224300352/2022	2022-10-19	Primary care	
EPI_ISL_16672939	hRSV/B/England/224300362/2022	2022-10-20	Primary care	
EPI_ISL_16672940	hRSV/A/England/224300367/2022	2022-10-19	Primary care	
EPI_ISL_16672941	hRSV/B/England/224300381/2022	2022-10-20	Primary care	
EPI_ISL_16672942	hRSV/A/England/224300384/2022	2022-10-21	Primary care	
EPI_ISL_16672943	hRSV/A/England/224300398/2022	2022-10-21	Community	
EPI_ISL_16672944	hRSV/A/England/224320540/2022	2022-10-19	Community	
EPI_ISL_16672945	hRSV/B/England/224340343/2022	2022-10-24	Primary care	
EPI_ISL_16672946	hRSV/A/England/224360285/2022	2022-10-24	Primary care	
EPI_ISL_16672947	hRSV/A/England/224360325/2022	2022-10-24	Community	
EPI_ISL_16672948	hRSV/A/England/224380368/2022	2022-10-26	Primary care	
EPI_ISL_16672949	hRSV/A/England/224400232/2022	2022-10-28	Primary care	
EPI_ISL_16672950	hRSV/A/England/224400354/2022	2022-10-27	Primary care	
EPI_ISL_16672951	hRSV/A/England/224400355/2022	2022-10-27	Primary care	
EPI_ISL_16672952	hRSV/A/England/224400359/2022	2022-10-24	Primary care	
EPI_ISL_16672953	hRSV/A/England/224400436/2022	2022-10-28	Community	
EPI_ISL_16672954	hRSV/A/England/224440380/2022	2022-10-31	Community	
EPI_ISL_16672955	hRSV/A/England/224440383/2022	2022-10-31	Community	
EPI_ISL_16672956	hRSV/A/England/224440387/2022	2022-10-31	Community	
EPI_ISL_16672957	hRSV/A/England/224460226/2022	2022-11-01	Community	
EPI_ISL_16672958	hRSV/A/England/224480233/2022	2022-10-02	Primary care	hAdV-C1: sequence unpublished
EPI_ISL_16672959	hRSV/A/England/224500210/2022	2022-11-02	Primary care	
EPI_ISL_16672960	hRSV/A/England/224500230/2022	2022-11-03	Primary care	hAdV-C5: sequence unpublished
EPI_ISL_16672961	hRSV/A/England/224500288/2022	2022-11-03	Community	
EPI_ISL_16672962	hRSV/A/England/224540254/2022	2022-11-07	Community	

GISAID_Accession_ID	GISAID_Virus_Name	Virus Collection Date	Source of Sample	Sequence confirmed co-infection comment (GISAID ID where available)
EPI_ISL_16672963	hRSV/A/England/224540710/2022	2022-11-07	Community	
EPI_ISL_16672964	hRSV/A/England/224560214/2022	2022-11-08	Primary care	
EPI_ISL_16672965	hRSV/A/England/224560221/2022	2022-11-08	Primary care	
EPI_ISL_16672966	hRSV/A/England/224560782/2022	2022-11-08	Community	
EPI_ISL_16672967	hRSV/A/England/224560793/2022	2022-11-08	Primary care	
EPI_ISL_16672968	hRSV/A/England/224560797/2022	2022-11-07	Primary care	
EPI_ISL_16672969	hRSV/A/England/224560811/2022	2022-11-08	Primary care	
EPI_ISL_16672970	hRSV/A/England/224640373/2022	2022-11-14	Primary care	
EPI_ISL_16672971	hRSV/A/England/224640406/2022	2022-11-14	Primary care	
EPI_ISL_16672972	hRSV/A/England/224660376/2022	2022-11-14	Primary care	
EPI_ISL_16672973	hRSV/A/England/224680483/2022	2022-11-17	Primary care	
EPI_ISL_16672974	hRSV/A/England/224680486/2022	2022-11-17	Primary care	
EPI_ISL_16672975	hRSV/A/England/224680489/2022	2022-11-10	Primary care	
EPI_ISL_16672976	hRSV/A/England/224680712/2022	2022-11-17	Community	
EPI_ISL_16672977	hRSV/A/England/224700499/2022	2022-11-18	Primary care	
EPI_ISL_16672978	hRSV/A/England/224700500/2022	2022-11-18	Primary care	
EPI_ISL_16672979	hRSV/A/England/224700501/2022	2022-11-18	Primary care	
EPI_ISL_16672980	hRSV/A/England/224700509/2022	2022-11-17	Primary care	
EPI_ISL_16672981	hRSV/A/England/224700515/2022	2022-11-18	Primary care	
EPI_ISL_16672982	hRSV/A/England/224700525/2022	2022-11-18	Primary care	
EPI_ISL_16672983	hRSV/A/England/224700533/2022	2022-11-18	Primary care	
EPI_ISL_16672984	hRSV/A/England/224700545/2022	2022-11-18	Primary care	
EPI_ISL_16672985	hRSV/A/England/224700584/2022	2022-11-18	Community	
EPI_ISL_16672986	hRSV/A/England/224720351/2022	2022-11-16	Primary care	
EPI_ISL_16672987	hRSV/A/England/224720361/2022	2022-11-18	Primary care	
EPI_ISL_16672988	hRSV/A/England/224720370/2022	2022-11-21	Primary care	
EPI_ISL_16672989	hRSV/A/England/224720372/2022	2022-11-21	Primary care	
EPI_ISL_16672990	hRSV/A/England/224740333/2022	2022-11-21	Primary care	
EPI_ISL_16672991	hRSV/A/England/224740346/2022	2022-11-22	Primary care	
EPI_ISL_16672992	hRSV/A/England/224740886/2022	2022-11-21	Primary care	
EPI_ISL_16672993	hRSV/A/England/224740889/2022	2022-11-21	Primary care	
EPI_ISL_16672994	hRSV/A/England/224780611/2022	2022-11-23	Primary care	
EPI_ISL_16672995	hRSV/A/England/224800319/2022	2022-11-25	Primary care	
EPI_ISL_16672996	hRSV/A/England/224800342/2022	2022-11-22	Primary care	
EPI_ISL_16672997	hRSV/A/England/224800345/2022	2022-11-22	Primary care	
EPI_ISL_16672998	hRSV/A/England/224800363/2022	2022-11-22	Primary care	
EPI_ISL_16708970	hRSV/A/England/E22004570/2019	2019-12-02	Secondary Care	
EPI_ISL_16708971	hRSV/A/England/E22004571/2019	2019-12-03	Secondary Care	
EPI_ISL_16708972	hRSV/A/England/E22004573/2019	2019-12-04	Secondary Care	
EPI_ISL_16708973	hRSV/B/England/E22004576/2019	2019-12-05	Secondary Care	
EPI_ISL_16708974	hRSV/B/England/E22004578/2019	2019-12-14	Secondary Care	
EPI_ISL_16708975	hRSV/A/England/E22004579/2019	2019-12-14	Secondary Care	
EPI_ISL_16708976	hRSV/A/England/E22004581/2019	2019-12-15	Secondary Care	
EPI_ISL_16708977	hRSV/A/England/E22004584/2019	2019-12-18	Secondary Care	
EPI_ISL_16708978	hRSV/B/England/E22004585/2019	2019-12-19	Secondary Care	

GISAID_Accession_ID	GISAID_Virus_Name	Virus Collection Date	Source of Sample	Sequence confirmed co-infection comment (GISAID ID where available)
EPI_ISL_16708979	hRSV/B/England/E22004587/2019	2019-12-21	Secondary Care	
EPI_ISL_16708980	hRSV/A/England/E22004588/2019	2019-12-23	Secondary Care	
EPI_ISL_16708981	hRSV/A/England/E22004589/2019	2019-12-23	Secondary Care	
EPI_ISL_16708982	hRSV/A/England/E22004592/2019	2019-12-25	Secondary Care	
EPI_ISL_16708983	hRSV/B/England/E22004594/2019	2019-12-27	Secondary Care	
EPI_ISL_16708984	hRSV/A/England/E22004596/2019	2019-12-28	Secondary Care	
EPI_ISL_16708985	hRSV/A/England/E22004599/2019	2019-12-30	Secondary Care	
EPI_ISL_16708986	hRSV/A/England/E22004603/2019	2019-12-31	Secondary Care	
EPI_ISL_16708987	hRSV/A/England/E22004607/2020	2020-01-02	Secondary Care	
EPI_ISL_16708988	hRSV/B/England/E22004608/2020	2020-01-19	Secondary Care	
EPI_ISL_16708989	hRSV/A/England/E22004609/2021	2021-07-22	Secondary Care	
EPI_ISL_16708990	hRSV/A/England/E22004610/2021	2021-08-02	Secondary Care	
EPI_ISL_16708991	hRSV/A/England/E22004611/2021	2021-08-02	Secondary Care	
EPI_ISL_16708992	hRSV/A/England/E22004612/2021	2021-09-16	Secondary Care	
EPI_ISL_16708993	hRSV/A/England/E22004613/2021	2021-09-19	Secondary Care	
EPI_ISL_16708994	hRSV/B/England/E22004614/2021	2021-09-24	Secondary Care	
EPI_ISL_16708995	hRSV/A/England/E22004617/2021	2021-09-27	Secondary Care	
EPI_ISL_16708996	hRSV/A/England/E22004618/2021	2021-10-06	Secondary Care	
EPI_ISL_16708997	hRSV/A/England/E22004619/2021	2021-10-07	Secondary Care	
EPI_ISL_16708998	hRSV/A/England/E22004620/2021	2021-10-08	Secondary Care	
EPI_ISL_16708999	hRSV/B/England/E22004621/2021	2021-10-10	Secondary Care	
EPI_ISL_16709000	hRSV/B/England/E22004622/2021	2021-10-13	Secondary Care	
EPI_ISL_16709001	hRSV/B/England/E22004623/2021	2021-10-16	Secondary Care	
EPI_ISL_16709002	hRSV/A/England/E22004625/2021	2021-10-18	Secondary Care	
EPI_ISL_16709003	hRSV/B/England/E22004626/2021	2021-10-20	Secondary Care	
EPI_ISL_16709004	hRSV/A/England/E22004628/2021	2021-10-26	Secondary Care	
EPI_ISL_16709005	hRSV/A/England/E22004630/2021	2021-11-11	Secondary Care	
EPI_ISL_16709006	hRSV/B/England/E22004631/2021	2021-11-12	Secondary Care	
EPI_ISL_16709007	hRSV/B/England/E22004632/2021	2021-11-14	Secondary Care	
EPI_ISL_16709008	hRSV/B/England/E22004633/2021	2021-11-16	Secondary Care	
EPI_ISL_16709009	hRSV/A/England/E22004636/2021	2021-12-24	Secondary Care	
EPI_ISL_16709010	hRSV/B/England/E22004637/2021	2021-08-02	Secondary Care	
EPI_ISL_16709011	hRSV/A/England/E22004638/2021	2021-09-28	Secondary Care	
EPI_ISL_16714277	hRSV/B/England/E22002506/2022	2022-08-06	Secondary Care	
EPI_ISL_16714278	hRSV/B/England/E22004639/2021	2021-11-15	Secondary Care	
EPI_ISL_16714279	hRSV/B/England/E22004640/2021	2021-11-15	Secondary Care	
EPI_ISL_16714280	hRSV/A/England/E22004641/2021	2021-11-18	Secondary Care	
EPI_ISL_16714281	hRSV/A/England/E22004643/2021	2021-11-23	Secondary Care	
EPI_ISL_16714282	hRSV/A/England/E22004644/2021	2021-11-24	Secondary Care	
EPI_ISL_16714283	hRSV/B/England/E22004645/2021	2021-11-26	Secondary Care	
EPI_ISL_16714284	hRSV/A/England/E22004646/2021	2021-11-28	Secondary Care	
EPI_ISL_16714285	hRSV/A/England/E22004647/2021	2021-11-30	Secondary Care	
EPI_ISL_16714286	hRSV/A/England/E22004649/2021	2021-12-01	Secondary Care	
EPI_ISL_16714287	hRSV/B/England/E22004651/2021	2021-11-29	Secondary Care	
EPI_ISL_16714288	hRSV/A/England/E22004652/2021	2021-12-04	Secondary Care	

GISAID_Accession_ID	GISAID_Virus_Name	Virus Collection Date	Source of Sample	Sequence confirmed co-infection comment (GISAID ID where available)
EPI_ISL_16714289	hRSV/B/England/E22004653/2021	2021-12-04	Secondary Care	
EPI_ISL_16714290	hRSV/A/England/E22004654/2021	2021-12-06	Secondary Care	
EPI_ISL_16714291	hRSV/B/England/E22004655/2021	2021-12-09	Secondary Care	
EPI_ISL_16714292	hRSV/A/England/E22004657/2021	2021-12-11	Secondary Care	
EPI_ISL_16714293	hRSV/A/England/E22004658/2021	2021-12-13	Secondary Care	
EPI_ISL_16714294	hRSV/B/England/E22004659/2021	2021-12-14	Secondary Care	
EPI_ISL_16714295	hRSV/A/England/E22004660/2021	2021-12-15	Secondary Care	
EPI_ISL_16714296	hRSV/A/England/E22004661/2021	2021-12-15	Secondary Care	
EPI_ISL_16714297	hRSV/A/England/E22004662/2021	2021-12-17	Secondary Care	
EPI_ISL_16714298	hRSV/B/England/E22004663/2021	2021-12-18	Secondary Care	
EPI_ISL_16714299	hRSV/A/England/E22004664/2021	2021-12-19	Secondary Care	
EPI_ISL_16714300	hRSV/A/England/E22004665/2022	2022-01-04	Secondary Care	
EPI_ISL_16714301	hRSV/A/England/E22004666/2022	2022-01-06	Secondary Care	
EPI_ISL_16714302	hRSV/A/England/E22004667/2022	2022-01-06	Secondary Care	
EPI_ISL_16714303	hRSV/A/England/E22004668/2021	2021-12-27	Secondary Care	
EPI_ISL_16714304	hRSV/A/England/E22004669/2022	2022-01-09	Secondary Care	
EPI_ISL_16714305	hRSV/B/England/E22004670/2022	2022-01-10	Secondary Care	
EPI_ISL_16714306	hRSV/B/England/E22004671/2022	2022-01-12	Secondary Care	
EPI_ISL_16714307	hRSV/B/England/E22004672/2021	2021-08-16	Secondary Care	
EPI_ISL_16714308	hRSV/A/England/E22004676/2021	2021-08-11	Secondary Care	
EPI_ISL_16714309	hRSV/A/England/E22004677/2021	2021-08-11	Secondary Care	
EPI_ISL_16714310	hRSV/A/England/E22004678/2021	2021-08-11	Secondary Care	
EPI_ISL_16714311	hRSV/A/England/E22004679/2021	2021-08-16	Secondary Care	
EPI_ISL_16714312	hRSV/A/England/E22004680/2021	2021-08-14	Secondary Care	
EPI_ISL_16714313	hRSV/B/England/E22004681/2021	2021-08-16	Secondary Care	
EPI_ISL_16714314	hRSV/A/England/E22004683/2021	2021-08-18	Secondary Care	
EPI_ISL_16714315	hRSV/B/England/E22004684/2021	2021-08-17	Secondary Care	
EPI_ISL_16714316	hRSV/A/England/E22004685/2021	2021-08-17	Secondary Care	
EPI_ISL_16714317	hRSV/A/England/E22004686/2021	2021-08-17	Secondary Care	
EPI_ISL_16714318	hRSV/A/England/E22004687/2021	2021-08-18	Secondary Care	
EPI_ISL_16714319	hRSV/B/England/E22004688/2021	2021-08-22	Secondary Care	
EPI_ISL_16714320	hRSV/A/England/E22004689/2021	2021-08-23	Secondary Care	
EPI_ISL_16714321	hRSV/A/England/E22004690/2021	2021-08-24	Secondary Care	
EPI_ISL_16714322	hRSV/A/England/E22004691/2021	2021-08-24	Secondary Care	
EPI_ISL_16714323	hRSV/B/England/E22004692/2021	2021-08-24	Secondary Care	
EPI_ISL_16714324	hRSV/A/England/E22004693/2021	2021-08-24	Secondary Care	
EPI_ISL_16714325	hRSV/A/England/E22004694/2021	2021-08-24	Secondary Care	
EPI_ISL_16714326	hRSV/A/England/E22004695/2021	2021-08-25	Secondary Care	
EPI_ISL_16714327	hRSV/B/England/E22004696/2021	2021-09-05	Secondary Care	
EPI_ISL_16714328	hRSV/B/England/E22004697/2021	2021-09-06	Secondary Care	
EPI_ISL_16714329	hRSV/A/England/E22004698/2021	2021-09-08	Secondary Care	
EPI_ISL_16714330	hRSV/B/England/E22004699/2021	2021-09-08	Secondary Care	
EPI_ISL_16714331	hRSV/A/England/E22004700/2021	2021-09-07	Secondary Care	
EPI_ISL_16714332	hRSV/B/England/E22004701/2021	2021-08-30	Secondary Care	
EPI_ISL_16714333	hRSV/B/England/E22004702/2021	2021-08-21	Secondary Care	

GISAID_Accession_ID	GISAID_Virus_Name	Virus Collection Date	Source of Sample	Sequence confirmed co-infection comment (GISAID ID where available)
EPI_ISL_16714334	hRSV/B/England/E22004703/2021	2021-09-08	Secondary Care	
EPI_ISL_16714335	hRSV/B/England/E22004706/2021	2021-09-10	Secondary Care	
EPI_ISL_16714336	hRSV/B/England/E22004707/2021	2021-09-23	Secondary Care	
EPI_ISL_16714337	hRSV/A/England/E22004708/2021	2021-09-25	Secondary Care	
EPI_ISL_16714338	hRSV/B/England/E22004709/2021	2021-09-24	Secondary Care	
EPI_ISL_16714339	hRSV/B/England/E22004710/2021	2021-09-24	Secondary Care	
EPI_ISL_16714340	hRSV/B/England/E22004711/2021	2021-09-24	Secondary Care	
EPI_ISL_16714341	hRSV/A/England/E22004712/2021	2021-09-24	Secondary Care	
EPI_ISL_16714342	hRSV/B/England/E22004713/2021	2021-09-24	Secondary Care	
EPI_ISL_16714343	hRSV/A/England/E22004714/2021	2021-09-25	Secondary Care	
EPI_ISL_16714344	hRSV/A/England/E22004715/2021	2021-09-25	Secondary Care	
EPI_ISL_16714345	hRSV/B/England/E22004716/2021	2021-09-26	Secondary Care	
EPI_ISL_16714346	hRSV/A/England/E22004717/2021	2021-09-26	Secondary Care	
EPI_ISL_16714347	hRSV/A/England/E22004718/2021	2021-09-27	Secondary Care	
EPI_ISL_16714348	hRSV/B/England/E22004719/2021	2021-09-27	Secondary Care	
EPI_ISL_16714349	hRSV/B/England/E22004721/2021	2021-09-28	Secondary Care	
EPI_ISL_16714350	hRSV/A/England/E22004722/2021	2021-10-01	Secondary Care	
EPI_ISL_16714351	hRSV/B/England/E22004723/2021	2021-10-02	Secondary Care	
EPI_ISL_16714352	hRSV/B/England/E22004724/2021	2021-10-02	Secondary Care	
EPI_ISL_16714353	hRSV/B/England/E22004725/2021	2021-10-02	Secondary Care	
EPI_ISL_16714354	hRSV/A/England/E22004726/2021	2021-09-22	Secondary Care	
EPI_ISL_16714355	hRSV/A/England/E22004727/2021	2021-09-22	Secondary Care	
EPI_ISL_16714356	hRSV/B/England/E22004728/2021	2021-10-04	Secondary Care	
EPI_ISL_16714357	hRSV/B/England/E22004730/2021	2021-10-05	Secondary Care	
EPI_ISL_16714358	hRSV/B/England/E22004731/2021	2021-10-04	Secondary Care	
EPI_ISL_16714359	hRSV/B/England/E22004732/2021	2021-10-06	Secondary Care	
EPI_ISL_16714360	hRSV/A/England/E22004733/2021	2021-10-06	Secondary Care	
EPI_ISL_16714361	hRSV/A/England/E22004734/2021	2021-10-09	Secondary Care	
EPI_ISL_16714362	hRSV/A/England/E22004735/2021	2021-10-08	Secondary Care	
EPI_ISL_16714363	hRSV/A/England/E22004736/2021	2021-10-10	Secondary Care	
EPI_ISL_16714364	hRSV/B/England/E22004737/2021	2021-10-10	Secondary Care	
EPI_ISL_16714365	hRSV/A/England/E22004739/2021	2021-10-11	Secondary Care	
EPI_ISL_16714366	hRSV/A/England/E22004740/2021	2021-10-11	Secondary Care	
EPI_ISL_16714367	hRSV/A/England/E22004741/2021	2021-10-11	Secondary Care	
EPI_ISL_16714368	hRSV/A/England/E22004742/2021	2021-10-11	Secondary Care	
EPI_ISL_16714369	hRSV/B/England/E22004743/2021	2021-10-12	Secondary Care	
EPI_ISL_16714370	hRSV/B/England/E22004744/2021	2021-10-12	Secondary Care	
EPI_ISL_16714371	hRSV/B/England/E22004745/2021	2021-10-12	Secondary Care	
EPI_ISL_16714372	hRSV/A/England/E22004746/2021	2021-10-13	Secondary Care	
EPI_ISL_16714373	hRSV/A/England/E22004747/2021	2021-10-13	Secondary Care	
EPI_ISL_16714374	hRSV/A/England/E22004748/2021	2021-10-13	Secondary Care	
EPI_ISL_16714375	hRSV/B/England/E22004749/2021	2021-10-14	Secondary Care	
EPI_ISL_16714376	hRSV/A/England/E22004750/2021	2021-10-13	Secondary Care	
EPI_ISL_16714377	hRSV/B/England/E22004751/2021	2021-10-14	Secondary Care	
EPI_ISL_16714378	hRSV/B/England/E22004752/2021	2021-10-14	Secondary Care	

GISAID_Accession_ID	GISAID_Virus_Name	Virus Collection Date	Source of Sample	Sequence confirmed co-infection comment (GISAID ID where available)
EPI_ISL_16714379	hRSV/A/England/E22004753/2021	2021-10-14	Secondary Care	
EPI_ISL_16714380	hRSV/B/England/E22004754/2021	2021-10-14	Secondary Care	
EPI_ISL_16714381	hRSV/A/England/E22004756/2021	2021-10-15	Secondary Care	
EPI_ISL_16714382	hRSV/B/England/E22004758/2021	2021-10-15	Secondary Care	
EPI_ISL_16714383	hRSV/A/England/E22004759/2021	2021-10-15	Secondary Care	
EPI_ISL_16714384	hRSV/B/England/E22004760/2021	2021-10-16	Secondary Care	
EPI_ISL_16714385	hRSV/B/England/E22004761/2021	2021-10-16	Secondary Care	
EPI_ISL_16714386	hRSV/B/England/E22004763/2021	2021-10-17	Secondary Care	
EPI_ISL_16714387	hRSV/B/England/E22004764/2021	2021-10-17	Secondary Care	
EPI_ISL_16714388	hRSV/A/England/E22004765/2021	2021-10-18	Secondary Care	
EPI_ISL_16714389	hRSV/B/England/E22004766/2021	2021-10-18	Secondary Care	
EPI_ISL_16714390	hRSV/A/England/E22004767/2021	2021-10-18	Secondary Care	
EPI_ISL_16714391	hRSV/A/England/E22004768/2021	2021-10-18	Secondary Care	
EPI_ISL_16714392	hRSV/A/England/E22004769/2021	2021-10-18	Secondary Care	
EPI_ISL_16714393	hRSV/A/England/E22004770/2021	2021-10-18	Secondary Care	
EPI_ISL_16714394	hRSV/B/England/E22004771/2021	2021-10-19	Secondary Care	
EPI_ISL_16714395	hRSV/A/England/E22004773/2021	2021-10-19	Secondary Care	
EPI_ISL_16714396	hRSV/A/England/E22004774/2021	2021-10-20	Secondary Care	
EPI_ISL_16714397	hRSV/A/England/E22004775/2021	2021-10-20	Secondary Care	
EPI_ISL_16714398	hRSV/A/England/E22004776/2021	2021-10-16	Secondary Care	
EPI_ISL_16714399	hRSV/A/England/E22004777/2021	2021-10-21	Secondary Care	
EPI_ISL_16714400	hRSV/A/England/E22004778/2021	2021-10-22	Secondary Care	
EPI_ISL_16714401	hRSV/A/England/E22004779/2021	2021-10-22	Secondary Care	
EPI_ISL_16714402	hRSV/A/England/E22004780/2021	2021-10-22	Secondary Care	
EPI_ISL_16714403	hRSV/A/England/E22004781/2021	2021-10-22	Secondary Care	
EPI_ISL_16714404	hRSV/A/England/E22004782/2021	2021-10-24	Secondary Care	
EPI_ISL_16714405	hRSV/A/England/E22004783/2021	2021-10-15	Secondary Care	
EPI_ISL_16714406	hRSV/A/England/E22004784/2021	2021-10-25	Secondary Care	
EPI_ISL_16714407	hRSV/A/England/E22004785/2021	2021-10-25	Secondary Care	
EPI_ISL_16714408	hRSV/B/England/E22004786/2021	2021-10-25	Secondary Care	
EPI_ISL_16714409	hRSV/A/England/E22004787/2021	2021-10-25	Secondary Care	
EPI_ISL_16714410	hRSV/B/England/E22004788/2021	2021-10-25	Secondary Care	
EPI_ISL_16714411	hRSV/A/England/E22004789/2021	2021-10-26	Secondary Care	
EPI_ISL_16714412	hRSV/A/England/E22004790/2021	2021-10-27	Secondary Care	
EPI_ISL_16714413	hRSV/B/England/E22004791/2021	2021-10-27	Secondary Care	
EPI_ISL_16714414	hRSV/A/England/E22004792/2021	2021-10-28	Secondary Care	
EPI_ISL_16714415	hRSV/A/England/E22004794/2021	2021-10-29	Secondary Care	
EPI_ISL_16714416	hRSV/A/England/E22004795/2021	2021-10-29	Secondary Care	
EPI_ISL_16714417	hRSV/A/England/E22004797/2021	2021-11-01	Secondary Care	
EPI_ISL_16714418	hRSV/B/England/E22004798/2021	2021-11-03	Secondary Care	
EPI_ISL_16714419	hRSV/A/England/E22004799/2021	2021-11-03	Secondary Care	
EPI_ISL_16714420	hRSV/A/England/E22004800/2021	2021-11-03	Secondary Care	
EPI_ISL_16714421	hRSV/B/England/E22004801/2021	2021-11-06	Secondary Care	
EPI_ISL_16714422	hRSV/B/England/E22004802/2021	2021-11-06	Secondary Care	
EPI_ISL_16714423	hRSV/B/England/E22004803/2021	2021-11-07	Secondary Care	

GISAID_Accession_ID	GISAID_Virus_Name	Virus Collection Date	Source of Sample	Sequence confirmed co-infection comment (GISAID ID where available)
EPI_ISL_16714424	hRSV/B/England/E22004804/2021	2021-11-07	Secondary Care	
EPI_ISL_16714425	hRSV/A/England/E22004805/2021	2021-11-07	Secondary Care	
EPI_ISL_16714426	hRSV/B/England/E22004806/2021	2021-11-07	Secondary Care	
EPI_ISL_16714427	hRSV/B/England/E22004807/2021	2021-11-07	Secondary Care	
EPI_ISL_16714428	hRSV/A/England/E22004808/2021	2021-11-07	Secondary Care	
EPI_ISL_16714429	hRSV/B/England/E22004809/2021	2021-11-08	Secondary Care	
EPI_ISL_16714430	hRSV/A/England/E22004810/2021	2021-11-10	Secondary Care	
EPI_ISL_16714431	hRSV/A/England/E22004811/2021	2021-11-10	Secondary Care	
EPI_ISL_16714432	hRSV/A/England/E22004812/2021	2021-11-10	Secondary Care	
EPI_ISL_16714433	hRSV/A/England/E22004813/2021	2021-11-11	Secondary Care	
EPI_ISL_16714434	hRSV/A/England/E22004815/2021	2021-11-14	Secondary Care	
EPI_ISL_16714435	hRSV/A/England/E22004816/2021	2021-11-14	Secondary Care	
EPI_ISL_16714436	hRSV/A/England/E22004817/2022	2022-01-16	Secondary Care	
EPI_ISL_16714437	hRSV/A/England/E22004818/2022	2022-01-16	Secondary Care	
EPI_ISL_16714438	hRSV/A/England/E22004819/2022	2022-01-18	Secondary Care	
EPI_ISL_16714439	hRSV/A/England/E22004820/2022	2022-01-21	Secondary Care	
EPI_ISL_16714440	hRSV/A/England/E22004821/2022	2022-01-24	Secondary Care	
EPI_ISL_16714441	hRSV/A/England/E22004822/2022	2022-01-25	Secondary Care	
EPI_ISL_16714442	hRSV/A/England/E22004823/2022	2022-01-27	Secondary Care	
EPI_ISL_16714443	hRSV/A/England/E22004825/2022	2022-01-29	Secondary Care	
EPI_ISL_16714444	hRSV/A/England/E22004826/2022	2022-02-06	Secondary Care	
EPI_ISL_16714445	hRSV/A/England/E22004827/2022	2022-02-06	Secondary Care	
EPI_ISL_16714446	hRSV/A/England/E22004828/2022	2022-02-09	Secondary Care	
EPI_ISL_16714447	hRSV/A/England/E22004829/2022	2022-02-15	Secondary Care	
EPI_ISL_16714448	hRSV/A/England/E22004830/2022	2022-02-20	Secondary Care	
EPI_ISL_16714449	hRSV/A/England/E22004831/2022	2022-02-21	Secondary Care	
EPI_ISL_16714450	hRSV/A/England/E22004832/2022	2022-02-24	Secondary Care	
EPI_ISL_16714451	hRSV/A/England/E22004835/2022	2022-03-06	Secondary Care	
EPI_ISL_16714452	hRSV/A/England/E22004837/2022	2022-05-05	Secondary Care	
EPI_ISL_16714453	hRSV/B/England/E22004838/2022	2022-05-04	Secondary Care	
EPI_ISL_16714454	hRSV/B/England/E22004839/2022	2022-05-14	Secondary Care	
EPI_ISL_16714455	hRSV/A/England/E22004840/2022	2022-06-13	Secondary Care	
EPI_ISL_16714456	hRSV/A/England/E22004842/2022	2022-07-21	Secondary Care	
EPI_ISL_16714457	hRSV/A/England/E22004843/2022	2022-07-21	Secondary Care	
EPI_ISL_16714458	hRSV/A/England/E22004844/2022	2022-07-23	Secondary Care	
EPI_ISL_16714459	hRSV/A/England/E22004845/2022	2022-07-23	Secondary Care	
EPI_ISL_16714460	hRSV/B/England/E22004846/2022	2022-07-28	Secondary Care	
EPI_ISL_16714461	hRSV/A/England/E22004847/2022	2022-07-28	Secondary Care	
EPI_ISL_16714462	hRSV/A/England/E22004848/2022	2022-07-28	Secondary Care	
EPI_ISL_16714463	hRSV/B/England/E22004849/2022	2022-07-31	Secondary Care	
EPI_ISL_16714464	hRSV/A/England/E22004851/2022	2022-07-31	Secondary Care	
EPI_ISL_16714465	hRSV/A/England/E22004853/2022	2022-07-30	Secondary Care	
EPI_ISL_16714466	hRSV/B/England/E22004854/2022	2022-07-31	Secondary Care	
EPI_ISL_16714467	hRSV/A/England/E22004855/2022	2022-08-03	Secondary Care	
EPI_ISL_16714468	hRSV/A/England/E22004856/2022	2022-08-03	Secondary Care	

GISAID_Accession_ID	GISAID_Virus_Name	Virus Collection Date	Source of Sample	Sequence confirmed co-infection comment (GISAID ID where available)
EPI_ISL_16714469	hRSV/B/England/E22004857/2022	2022-08-06	Secondary Care	
EPI_ISL_16714470	hRSV/B/England/E22004858/2022	2022-06-07	Secondary Care	
EPI_ISL_16714471	hRSV/A/England/E22004859/2022	2022-08-27	Secondary Care	
EPI_ISL_16714472	hRSV/B/England/E22004860/2022	2022-08-29	Secondary Care	
EPI_ISL_16714473	hRSV/A/England/E22004861/2022	2022-08-31	Secondary Care	
EPI_ISL_16714474	hRSV/A/England/212321090/2021	2021-06-07	Primary care	
EPI_ISL_16714475	hRSV/A/England/212641418/2021	2021-06-28	Primary care	
EPI_ISL_16714476	hRSV/A/England/212821957/2021	2021-07-09	Primary care	
EPI_ISL_16714477	hRSV/A/England/212861233/2021	2021-07-15	Primary care	
EPI_ISL_16714478	hRSV/A/England/212861268/2021	2021-07-14	Community	
EPI_ISL_16714479	hRSV/A/England/212862350/2021	2021-07-14	Primary care	
EPI_ISL_16714480	hRSV/A/England/212881838/2021	2021-07-14	Primary care	
EPI_ISL_16714481	hRSV/A/England/212881839/2021	2021-07-14	Primary care	
EPI_ISL_16714482	hRSV/A/England/212891315/2021	2021-07-15	Primary care	
EPI_ISL_16714483	hRSV/A/England/212921511/2021	2021-07-20	Primary care	
EPI_ISL_16714484	hRSV/A/England/212921582/2021	2021-07-16	Primary care	
EPI_ISL_16714485	hRSV/A/England/212942129/2021	2021-07-19	Primary care	
EPI_ISL_16714486	hRSV/A/England/212961949/2021	2021-07-19	Primary care	
EPI_ISL_16714487	hRSV/A/England/212981674/2021	2021-07-22	Primary care	
EPI_ISL_16714488	hRSV/A/England/213001202/2021	2021-07-23	Primary care	
EPI_ISL_16714489	hRSV/A/England/213001203/2021	2021-07-23	Primary care	
EPI_ISL_16714490	hRSV/A/England/213021530/2021	2021-07-26	Primary care	
EPI_ISL_16714491	hRSV/A/England/213041267/2021	2021-07-26	Primary care	
EPI_ISL_16714492	hRSV/A/England/213041528/2021	2021-07-26	Primary care	
EPI_ISL_16714493	hRSV/A/England/213081274/2021	2021-07-28	Primary care	
EPI_ISL_16714494	hRSV/A/England/213161381/2021	2021-08-04	Primary care	
EPI_ISL_16714495	hRSV/A/England/213181237/2021	2021-08-05	Primary care	
EPI_ISL_16714496	hRSV/A/England/213181772/2021	2021-08-05	Primary care	
EPI_ISL_16714497	hRSV/A/England/213181774/2021	2021-08-05	Primary care	
EPI_ISL_16714498	hRSV/A/England/213190690/2021	2021-08-05	Primary care	
EPI_ISL_16714499	hRSV/A/England/213241029/2021	2021-08-06	Primary care	
EPI_ISL_16714500	hRSV/A/England/213241367/2021	2021-08-06	Primary care	
EPI_ISL_16714501	hRSV/A/England/213261327/2021	2021-08-10	Primary care	
EPI_ISL_16714502	hRSV/A/England/213341039/2021	2021-08-17	Primary care	
EPI_ISL_16714503	hRSV/A/England/213341594/2021	2021-08-16	Primary care	
EPI_ISL_16714504	hRSV/A/England/213380625/2021	2021-08-17	Primary care	
EPI_ISL_16714505	hRSV/A/England/213382574/2021	2021-08-19	Primary care	
EPI_ISL_16714506	hRSV/A/England/213382577/2021	2021-08-19	Community	
EPI_ISL_16714507	hRSV/A/England/213390859/2021	2021-08-19	Community	
EPI_ISL_16714508	hRSV/A/England/213390925/2021	2021-08-16	Primary care	
EPI_ISL_16714509	hRSV/A/England/213400832/2021	2021-08-20	Primary care	
EPI_ISL_16714510	hRSV/A/England/213421904/2021	2021-08-20	Primary care	
EPI_ISL_16714511	hRSV/A/England/213421917/2021	2021-08-23	Community	
EPI_ISL_16714512	hRSV/A/England/213441230/2021	2021-08-20	Primary care	
EPI_ISL_16714513	hRSV/A/England/213461198/2021	2021-08-25	Primary care	

GISAID_Accession_ID	GISAID_Virus_Name	Virus Collection Date	Source of Sample	Sequence confirmed co-infection comment (GISAID ID where available)
EPI_ISL_16714514	hRSV/A/England/213490871/2021	2021-08-27	Primary care	
EPI_ISL_16714515	hRSV/A/England/213520380/2021	2021-08-27	Community	
EPI_ISL_16714516	hRSV/A/England/213561087/2021	2021-09-02	Primary care	
EPI_ISL_16714517	hRSV/A/England/213561859/2021	2021-08-31	Primary care	
EPI_ISL_16714518	hRSV/A/England/213590417/2021	2021-09-01	Primary care	
EPI_ISL_16714519	hRSV/A/England/213601059/2021	2021-09-03	Primary care	
EPI_ISL_16714520	hRSV/A/England/213621405/2021	2021-09-03	Primary care	
EPI_ISL_16714521	hRSV/A/England/213641068/2021	2021-09-06	Primary care	
EPI_ISL_16714522	hRSV/A/England/213691283/2021	2021-09-09	Primary care	
EPI_ISL_16714523	hRSV/A/England/213740766/2021	2021-09-13	Primary care	
EPI_ISL_16714524	hRSV/A/England/213741603/2021	2021-09-13	Primary care	
EPI_ISL_16714525	hRSV/A/England/213761195/2021	2021-09-14	Primary care	
EPI_ISL_16714526	hRSV/A/England/213790799/2021	2021-09-16	Primary care	
EPI_ISL_16714527	hRSV/A/England/213790800/2021	2021-09-16	Primary care	
EPI_ISL_16714528	hRSV/A/England/213790804/2021	2021-09-16	Primary care	
EPI_ISL_16714529	hRSV/A/England/213790805/2021	2021-09-16	Primary care	
EPI_ISL_16714530	hRSV/A/England/213790807/2021	2021-09-16	Primary care	
EPI_ISL_16714531	hRSV/A/England/213790835/2021	2021-09-17	Community	
EPI_ISL_16714532	hRSV/A/England/213821561/2021	2021-09-18	Community	
EPI_ISL_16714533	hRSV/A/England/213861342/2021	2021-09-22	Primary care	
EPI_ISL_16714534	hRSV/A/England/213881402/2021	2021-09-24	Community	
EPI_ISL_16714535	hRSV/A/England/213890883/2021	2021-09-24	Community	
EPI_ISL_16714536	hRSV/A/England/213891284/2021	2021-09-25	Community	
EPI_ISL_16714537	hRSV/A/England/213941084/2021	2021-09-26	Community	
EPI_ISL_16714538	hRSV/A/England/213941085/2021	2021-09-26	Community	
EPI_ISL_16714539	hRSV/A/England/213941086/2021	2021-09-25	Community	
EPI_ISL_16714540	hRSV/A/England/213961695/2021	2021-09-29	Primary care	
EPI_ISL_16714541	hRSV/A/England/213981432/2021	2021-09-29	Primary care	
EPI_ISL_16714542	hRSV/A/England/213981474/2021	2021-09-29	Primary care	
EPI_ISL_16714543	hRSV/A/England/214022040/2021	2021-10-04	Community	
EPI_ISL_16714544	hRSV/A/England/214061276/2021	2021-10-06	Community	
EPI_ISL_16714545	hRSV/A/England/214081525/2021	2021-10-06	Primary care	
EPI_ISL_16714546	hRSV/A/England/214121787/2021	2021-10-11	Community	
EPI_ISL_16714547	hRSV/A/England/214121889/2021	2021-10-08	Primary care	
EPI_ISL_16714548	hRSV/A/England/214141326/2021	2021-10-13	Primary care	
EPI_ISL_16714549	hRSV/A/England/214141394/2021	2021-10-11	Primary care	
EPI_ISL_16714550	hRSV/A/England/214141397/2021	2021-10-11	Primary care	
EPI_ISL_16714551	hRSV/A/England/214141399/2021	2021-10-12	Primary care	
EPI_ISL_16714552	hRSV/A/England/214181409/2021	2021-10-13	Primary care	
EPI_ISL_16714553	hRSV/A/England/214190772/2021	2021-10-13	Primary care	
EPI_ISL_16714554	hRSV/A/England/214221869/2021	2021-10-18	Primary care	
EPI_ISL_16714555	hRSV/A/England/214221871/2021	2021-10-18	Primary care	
EPI_ISL_16714556	hRSV/A/England/214221873/2021	2021-10-18	Primary care	
EPI_ISL_16714557	hRSV/A/England/214301019/2021	2021-10-22	Primary care	
EPI_ISL_16714558	hRSV/A/England/214341765/2021	2021-10-25	Primary care	

GISAID_Accession_ID	GISAID_Virus_Name	Virus Collection Date	Source of Sample	Sequence confirmed co-infection comment (GISAID ID where available)
EPI_ISL_16714559	hRSV/A/England/214341771/2021	2021-10-25	Primary care	
EPI_ISL_16714560	hRSV/A/England/214341774/2021	2021-10-25	Primary care	
EPI_ISL_16714561	hRSV/A/England/214341776/2021	2021-10-25	Primary care	
EPI_ISL_16714562	hRSV/A/England/214481428/2021	2021-11-03	Primary care	
EPI_ISL_16714563	hRSV/A/England/214481430/2021	2021-11-03	Primary care	
EPI_ISL_16714564	hRSV/A/England/214490657/2021	2021-11-05	Primary care	
EPI_ISL_16714565	hRSV/A/England/214640633/2021	2021-11-15	Primary care	
EPI_ISL_16714566	hRSV/A/England/214740680/2021	2021-11-22	Primary care	
EPI_ISL_16714567	hRSV/A/England/214820889/2021	2021-11-29	Primary care	
EPI_ISL_16714568	hRSV/A/England/214860483/2021	2021-11-29	Primary care	
EPI_ISL_16714569	hRSV/A/England/214990484/2021	2021-12-10	Primary care	
EPI_ISL_16714570	hRSV/A/England/214990495/2021	2021-12-08	Primary care	
EPI_ISL_16714571	hRSV/A/England/220120599/2021	2021-12-30	Primary care	
EPI_ISL_16714572	hRSV/B/England/220181187/2022	2022-01-05	Primary care	hAdV-C2: sequence unpublished
EPI_ISL_16714573	hRSV/B/England/220221969/2022	2022-01-10	Primary care	
EPI_ISL_16714574	hRSV/B/England/220360535/2022	2022-01-19	Community	
EPI_ISL_16714575	hRSV/A/England/220800339/2022	2022-02-18	Primary care	
EPI_ISL_16714576	hRSV/B/England/220800340/2022	2022-02-17	Primary care	
EPI_ISL_16714577	hRSV/A/England/220820510/2022	2022-02-21	Primary care	
EPI_ISL_16714578	hRSV/B/England/220940657/2022	2022-02-28	Primary care	Coxsackievirus B4: sequence unpublished
EPI_ISL_16714579	hRSV/A/England/221141101/2022	2022-03-16	Primary care	
EPI_ISL_16714580	hRSV/B/England/221480714/2022	2022-04-07	Primary care	
EPI_ISL_16714581	hRSV/B/England/221541019/2022	2022-04-11	Primary care	
EPI_ISL_16714582	hRSV/A/England/221560395/2022	2022-04-13	Primary care	
EPI_ISL_16714583	hRSV/B/England/221920518/2022	2022-05-09	Primary care	
EPI_ISL_16714584	hRSV/B/England/221960005/2022	2022-05-10	Primary care	
EPI_ISL_16714585	hRSV/B/England/222100621/2022	2022-05-19	Primary care	Coxsackievirus B4: sequence unpublished
EPI_ISL_16714586	hRSV/B/England/222140485/2022	2022-05-24	Primary care	
EPI_ISL_16714587	hRSV/B/England/222140587/2022	2022-05-24	Primary care	
EPI_ISL_16714588	hRSV/A/England/222200693/2022	2022-05-24	Primary care	
EPI_ISL_16714589	hRSV/B/England/222440297/2022	2022-06-13	Primary care	
EPI_ISL_16714590	hRSV/B/England/222440300/2022	2022-06-13	Primary care	
EPI_ISL_16714591	hRSV/A/England/222460286/2022	2022-06-13	Primary care	Enterovirus VP1 Genotype-HRV-A55: sequence unpublished
EPI_ISL_16714592	hRSV/B/England/222460287/2022	2022-06-14	Primary care	
EPI_ISL_16714593	hRSV/A/England/222460290/2022	2022-06-14	Primary care	
EPI_ISL_16714594	hRSV/B/England/222540381/2022	2022-06-18	Community	
EPI_ISL_16714595	hRSV/B/England/E21000654/2021	2021-05-24	Secondary Care	
EPI_ISL_16714596	hRSV/B/England/E21000655/2021	2021-05-23	Secondary Care	
EPI_ISL_16714597	hRSV/B/England/E21000677/2021	2021-06-01	Secondary Care	
EPI_ISL_16714598	hRSV/B/England/E21000679/2021	2021-06-04	Secondary Care	
EPI_ISL_16714599	hRSV/B/England/E21000680/2021	2021-06-01	Secondary Care	
EPI_ISL_16714600	hRSV/B/England/E21000681/2021	2021-06-01	Secondary Care	
EPI_ISL_16714601	hRSV/A/England/E21000682/2021	2021-06-03	Secondary Care	
EPI_ISL_16714602	hRSV/B/England/E21000683/2021	2021-06-05	Secondary Care	
EPI_ISL_16714603	hRSV/B/England/E21000687/2021	2021-06-09	Secondary Care	

GISAID_Accession_ID	GISAID_Virus_Name	Virus Collection Date	Source of Sample	Sequence confirmed co-infection comment (GISAID ID where available)
EPI_ISL_16714604	hRSV/B/England/E21000988/2021	2021-06-18	Secondary Care	
EPI_ISL_16714605	hRSV/B/England/E21000989/2021	2021-06-18	Secondary Care	
EPI_ISL_16714606	hRSV/A/England/E21001055/2021	2021-06-24	Secondary Care	
EPI_ISL_16714607	hRSV/B/England/E21001082/2021	2021-07-02	Secondary Care	
EPI_ISL_16714608	hRSV/B/England/E21001083/2021	2021-07-06	Secondary Care	
EPI_ISL_16714609	hRSV/B/England/E21001085/2021	2021-07-07	Secondary Care	
EPI_ISL_16714610	hRSV/B/England/E21001086/2021	2021-07-06	Secondary Care	
EPI_ISL_16714611	hRSV/A/England/E21001088/2021	2021-07-07	Secondary Care	
EPI_ISL_16714612	hRSV/B/England/E21001089/2021	2021-07-06	Secondary Care	
EPI_ISL_16714613	hRSV/B/England/E21001090/2021	2021-07-07	Secondary Care	
EPI_ISL_16714614	hRSV/B/England/E21001112/2021	2021-07-13	Secondary Care	
EPI_ISL_16714615	hRSV/B/England/E21001113/2021	2021-07-13	Secondary Care	
EPI_ISL_16714616	hRSV/B/England/E21001472/2021	2021-11-12	Secondary Care	
EPI_ISL_16714617	hRSV/A/England/E21001473/2021	2021-11-11	Secondary Care	
EPI_ISL_16714618	hRSV/B/England/E21001476/2021	2021-10-04	Secondary Care	
EPI_ISL_16714619	hRSV/A/England/E21001477/2021	2021-10-04	Secondary Care	
EPI_ISL_16714620	hRSV/B/England/212590674/2021	2021-06-24	Primary care	
EPI_ISL_16714621	hRSV/B/England/212690869/2021	2021-06-30	Primary care	
EPI_ISL_16714622	hRSV/B/England/212781443/2021	2021-07-08	Primary care	
EPI_ISL_16714623	hRSV/B/England/212821959/2021	2021-07-09	Primary care	
EPI_ISL_16714624	hRSV/B/England/212862435/2021	2021-07-14	Primary care	
EPI_ISL_16714625	hRSV/B/England/212940615/2021	2021-07-15	Primary care	
EPI_ISL_16714626	hRSV/B/England/213081473/2021	2021-07-30	Community	
EPI_ISL_16714627	hRSV/B/England/213141478/2021	2021-08-02	Primary care	
EPI_ISL_16714628	hRSV/B/England/213141510/2021	2021-08-02	Primary care	
EPI_ISL_16714629	hRSV/B/England/213221933/2021	2021-08-09	Primary care	
EPI_ISL_16714630	hRSV/B/England/213341593/2021	2021-08-16	Primary care	
EPI_ISL_16714631	hRSV/B/England/213361340/2021	2021-08-18	Primary care	
EPI_ISL_16714632	hRSV/B/England/213382572/2021	2021-08-19	Primary care	
EPI_ISL_16714633	hRSV/B/England/213382573/2021	2021-08-18	Primary care	
EPI_ISL_16714634	hRSV/B/England/213441523/2021	2021-08-24	Primary care	
EPI_ISL_16714635	hRSV/B/England/213490878/2021	2021-08-27	Primary care	
EPI_ISL_16714636	hRSV/B/England/213520907/2021	2021-08-28	Community	
EPI_ISL_16714637	hRSV/B/England/213541171/2021	2021-08-29	Community	
EPI_ISL_16714638	hRSV/B/England/213590415/2021	2021-09-01	Primary care	
EPI_ISL_16714639	hRSV/B/England/213601053/2021	2021-09-03	Primary care	
EPI_ISL_16714640	hRSV/B/England/213640636/2021	2021-09-03	Primary care	
EPI_ISL_16714641	hRSV/B/England/213661859/2021	2021-09-08	Community	
EPI_ISL_16714642	hRSV/B/England/213740767/2021	2021-09-09	Primary care	
EPI_ISL_16714643	hRSV/B/England/213761211/2021	2021-09-14	Primary care	
EPI_ISL_16714644	hRSV/B/England/213781197/2021	2021-09-16	Community	
EPI_ISL_16714645	hRSV/B/England/213821243/2021	2021-09-17	Primary care	
EPI_ISL_16714646	hRSV/B/England/213881449/2021	2021-09-24	Community	
EPI_ISL_16714647	hRSV/B/England/213890895/2021	2021-09-23	Primary care	
EPI_ISL_16714648	hRSV/B/England/213890936/2021	2021-09-22	Primary care	

GISAID_Accession_ID	GISAID_Virus_Name	Virus Collection Date	Source of Sample	Sequence confirmed co-infection comment (GISAID ID where available)
EPI_ISL_16714649	hRSV/B/England/213921117/2021	2021-09-27	Primary care	
EPI_ISL_16714650	hRSV/B/England/213961642/2021	2021-09-28	Primary care	
EPI_ISL_16714651	hRSV/B/England/213981418/2021	2021-09-27	Primary care	
EPI_ISL_16714652	hRSV/B/England/213981591/2021	2021-09-28	Community	
EPI_ISL_16714653	hRSV/B/England/214000676/2021	2021-10-01	Community	
EPI_ISL_16714654	hRSV/B/England/214022045/2021	2021-10-04	Community	
EPI_ISL_16714655	hRSV/B/England/214041254/2021	2021-10-04	Primary care	
EPI_ISL_16714656	hRSV/B/England/214041256/2021	2021-10-05	Primary care	
EPI_ISL_16714657	hRSV/B/England/214041499/2021	2021-10-04	Primary care	
EPI_ISL_16714658	hRSV/B/England/214081448/2021	2021-10-08	Community	
EPI_ISL_16714659	hRSV/B/England/214121653/2021	2021-10-11	Primary care	
EPI_ISL_16714660	hRSV/B/England/214190770/2021	2021-10-14	Primary care	
EPI_ISL_16714661	hRSV/B/England/214221842/2021	2021-10-15	Primary care	
EPI_ISL_16714662	hRSV/B/England/214241604/2021	2021-10-18	Primary care	
EPI_ISL_16714663	hRSV/B/England/214261609/2021	2021-10-20	Primary care	
EPI_ISL_16714664	hRSV/B/England/214290648/2021	2021-10-21	Primary care	
EPI_ISL_16714665	hRSV/B/England/214290650/2021	2021-10-23	Primary care	
EPI_ISL_16714666	hRSV/B/England/214300634/2021	2021-10-21	Primary care	
EPI_ISL_16714667	hRSV/B/England/214301018/2021	2021-10-22	Primary care	
EPI_ISL_16714668	hRSV/B/England/214321616/2021	2021-10-22	Primary care	
EPI_ISL_16714669	hRSV/B/England/214541256/2021	2021-11-08	Primary care	
EPI_ISL_16714670	hRSV/B/England/214541259/2021	2021-11-08	Primary care	
EPI_ISL_16714671	hRSV/B/England/214580618/2021	2021-11-09	Primary care	
EPI_ISL_16714672	hRSV/B/England/214580619/2021	2021-11-11	Primary care	
EPI_ISL_16714673	hRSV/B/England/214741260/2021	2021-11-22	Primary care	
EPI_ISL_16714674	hRSV/B/England/214780600/2021	2021-11-26	Primary care	
EPI_ISL_16714675	hRSV/B/England/214790181/2021	2021-11-25	Primary care	
EPI_ISL_16714676	hRSV/B/England/214800622/2021	2021-11-26	Community	
EPI_ISL_16714677	hRSV/B/England/214860296/2021	2021-12-02	Community	
EPI_ISL_16714678	hRSV/B/England/214860480/2021	2021-11-30	Primary care	
EPI_ISL_16714679	hRSV/B/England/214881089/2021	2021-12-01	Primary care	
EPI_ISL_16714680	hRSV/B/England/214940980/2021	2021-12-03	Primary care	
EPI_ISL_16714681	hRSV/B/England/214990516/2021	2021-12-09	Primary care	
EPI_ISL_16714682	hRSV/B/England/215041316/2021	2021-12-14	Community	
EPI_ISL_16714683	hRSV/B/England/215080975/2021	2021-12-13	Primary care	
EPI_ISL_16714684	hRSV/B/England/215090369/2021	2021-12-17	Primary care	
EPI_ISL_16714685	hRSV/B/England/215100699/2021	2021-12-15	Primary care	
EPI_ISL_16714686	hRSV/B/England/215160950/2021	2021-12-21	Primary care	
EPI_ISL_16714687	hRSV/B/England/215261549/2021	2021-12-29	Primary care	
EPI_ISL_16714688	hRSV/A/England/222680368/2022	2022-06-29	Primary care	
EPI_ISL_16714689	hRSV/A/England/222700196/2022	2022-06-30	Primary care	
EPI_ISL_16714690	hRSV/A/England/222780573/2022	2022-07-07	Community	
EPI_ISL_16714691	hRSV/A/England/222860363/2022	2022-07-12	Primary care	
EPI_ISL_16714692	hRSV/A/England/222900610/2022	2022-07-15	Primary care	
EPI_ISL_16714693	hRSV/A/England/222920368/2022	2022-07-18	Primary care	

GISAID_Accession_ID	GISAID_Virus_Name	Virus Collection Date	Source of Sample	Sequence confirmed co-infection comment (GISAID ID where available)
EPI_ISL_16714694	hRSV/A/England/222980395/2022	2022-07-22	Primary care	
EPI_ISL_16714695	hRSV/A/England/223020259/2022	2022-07-23	Primary care	
EPI_ISL_16714696	hRSV/A/England/223180441/2022	2022-08-04	Community	
EPI_ISL_16714697	hRSV/A/England/223380298/2022	2022-08-18	Community	
EPI_ISL_16714698	hRSV/A/England/223400459/2022	2022-08-18	Primary care	
EPI_ISL_16714699	hRSV/A/England/223700318/2022	2022-09-08	Community	
EPI_ISL_16714700	hRSV/A/England/223700678/2022	2022-09-09	Community	
EPI_ISL_16714701	hRSV/A/England/223700680/2022	2022-09-09	Community	
EPI_ISL_16714702	hRSV/A/England/223740499/2022	2022-09-13	Primary care	
EPI_ISL_16714703	hRSV/A/England/223900355/2022	2022-09-21	Primary care	
EPI_ISL_16714704	hRSV/A/England/224140297/2022	2022-10-07	Community	
EPI_ISL_16714705	hRSV/B/England/222680362/2022	2022-06-29	Primary care	
EPI_ISL_16714706	hRSV/B/England/222680367/2022	2022-06-30	Primary care	
EPI_ISL_16714707	hRSV/B/England/222700198/2022	2022-06-30	Primary care	
EPI_ISL_16714708	hRSV/B/England/222800222/2022	2022-07-08	Primary care	
EPI_ISL_16714709	hRSV/B/England/222800225/2022	2022-07-07	Primary care	
EPI_ISL_16714710	hRSV/B/England/222900246/2022	2022-07-15	Community	
EPI_ISL_16714711	hRSV/B/England/222960410/2022	2022-07-19	Primary care	
EPI_ISL_16714712	hRSV/B/England/223000663/2022	2022-07-22	Primary care	
EPI_ISL_16714713	hRSV/B/England/223020256/2022	2022-08-25	Primary care	
EPI_ISL_16714714	hRSV/B/England/223020257/2022	2022-07-25	Primary care	
EPI_ISL_16714715	hRSV/B/England/223040354/2022	2022-07-25	Primary care	
EPI_ISL_16714716	hRSV/B/England/223040355/2022	2022-07-25	Primary care	
EPI_ISL_16714717	hRSV/B/England/223040360/2022	2022-07-25	Primary care	
EPI_ISL_16714718	hRSV/B/England/223100235/2022	2022-07-29	Primary care	
EPI_ISL_16714719	hRSV/B/England/223120225/2022	2022-08-01	Primary care	
EPI_ISL_16714720	hRSV/B/England/223160348/2022	2022-08-01	Primary care	
EPI_ISL_16714721	hRSV/B/England/223160352/2022	2022-08-03	Community	
EPI_ISL_16714722	hRSV/B/England/223220626/2022	2022-08-08	Primary care	
EPI_ISL_16714723	hRSV/B/England/223280688/2022	2022-08-10	Primary care	
EPI_ISL_16714724	hRSV/B/England/223680616/2022	2022-09-05	Primary care	
EPI_ISL_16714725	hRSV/B/England/223700311/2022	2022-09-07	Primary care	
EPI_ISL_16714726	hRSV/B/England/223720344/2022	2022-09-12	Primary care	
EPI_ISL_16714727	hRSV/B/England/223720348/2022	2022-09-09	Primary care	
EPI_ISL_16714728	hRSV/B/England/223720784/2022	2022-09-12	Primary care	
EPI_ISL_16714729	hRSV/B/England/223760511/2022	2022-09-14	Community	
EPI_ISL_16714730	hRSV/B/England/223900362/2022	2022-09-23	Primary care	
EPI_ISL_16714731	hRSV/B/England/223920631/2022	2022-09-26	Primary care	
EPI_ISL_16714732	hRSV/B/England/223960657/2022	2022-09-28	Primary care	Enterovirus VP1 Genotype - HRV-C15: sequence unpublished
EPI_ISL_16714733	hRSV/B/England/224040470/2022	2022-10-03	Primary care	
EPI_ISL_16714734	hRSV/B/England/224080246/2022	2022-10-05	Primary care	
EPI_ISL_16714735	hRSV/B/England/224080249/2022	2022-10-06	Primary care	
EPI_ISL_16714736	hRSV/B/England/224080278/2022	2022-10-05	Community	
EPI_ISL_16714737	hRSV/B/England/224100285/2022	2022-10-07	Primary care	
EPI_ISL_16714738	hRSV/B/England/224140296/2022	2022-10-07	Community	

GISAID_Accession_ID	GISAID_Virus_Name	Virus Collection Date	Source of Sample	Sequence confirmed co-infection comment (GISAID ID where available)
EPI_ISL_16714739	hRSV/B/England/224140361/2022	2022-10-10	Primary care	
EPI_ISL_16714740	hRSV/A/England/224180362/2022	2022-10-11	Primary care	
EPI_ISL_16714741	hRSV/B/England/224200121/2022	2022-10-10	Primary care	
EPI_ISL_16714742	hRSV/B/England/224200126/2022	2022-10-10	Community	
EPI_ISL_16714743	hRSV/A/England/224200524/2022	2022-10-13	Primary care	
EPI_ISL_16737023	hRSV/B/England/224400362/2022	2022-10-28	Community	Coxsackievirus B4: sequence unpublished
EPI_ISL_16737024	hRSV/B/England/224560803/2022	2022-11-08	Community	
EPI_ISL_16737025	hRSV/B/England/224740340/2022	2022-11-22	Community	
EPI_ISL_16737026	hRSV/B/England/224880477/2022	2022-11-21	Community	
EPI_ISL_16737027	hRSV/B/England/224880479/2022	2022-11-29	Community	
EPI_ISL_16737028	hRSV/B/England/224880480/2022	2022-11-29	Community	
EPI_ISL_16737029	hRSV/A/England/224900480/2022	2022-11-28	Community	hRSV-B co-infection: EPI_ISL_17297218 (Ct RSV-A 22.3; Ct RSV-B 19.6; Age 2 y.o.a.)
EPI_ISL_16737030	hRSV/A/England/E22004673/2021	2021-08-15	Secondary Care	hRSV-B co-infection: EPI_ISL_16737031 (Ct RSV-A 15.26; Ct RSV-B 20.77; Age <2 y.o.a.)
EPI_ISL_16737031	hRSV/B/England/E22004673/2021	2021-08-15	Secondary Care	hRSV-A co-infection: EPI_ISL_16737030 (Ct RSV-A 15.26; Ct RSV-B 20.77; Age <2 y.o.a.)
EPI_ISL_16737032	hRSV/B/England/E22004674/2021	2021-08-15	Secondary Care	RSV-A co-infection: RSV-A WG sequence not obtained (Ct RSV-A 20.61; Ct RSV-B 15.02; Age <2 y.o.a.)
EPI_ISL_16737033	hRSV/A/England/E22004675/2021	2021-08-11	Secondary Care	hRSV-B co-infection: EPI_ISL_16737034 (Ct RSV-A 20.53; Ct RSV-B 14.96; Age <2 y.o.a.)
EPI_ISL_16737034	hRSV/B/England/E22004675/2021	2021-08-11	Secondary Care	hRSV-A co-infection: EPI_ISL_16737033 (Ct RSV-A 20.53; Ct RSV-B 14.96; Age <2 y.o.a.)
EPI_ISL_16737035	hRSV/B/England/E22004720/2021	2021-09-23	Secondary Care	
EPI_ISL_16737036	hRSV/B/England/E22004738/2021	2021-10-10	Secondary Care	RSV-A co-infection: RSV-A WG sequence not obtained (Ct RSV-A 19.85; Ct RSV-B 15.05; Age <2 y.o.a.)
EPI_ISL_16737037	hRSV/B/England/E22004755/2021	2021-10-14	Secondary Care	RSV-A co-infection: RSV-A WG sequence not obtained (Ct RSV-A 22.60; Ct RSV-B 17.10; Age <2 y.o.a.)
EPI_ISL_16737038	hRSV/A/England/E22004796/2021	2021-10-31	Secondary Care	
EPI_ISL_16737039	hRSV/B/England/E22004836/2022	2022-04-22	Secondary Care	RSV-A co-infection: RSV-A WG sequence not obtained (Ct RSV-A 26.62; Ct RSV-B 20.96; Age <2 y.o.a.)
EPI_ISL_16746474	hRSV/B/England/224500223/2022	2022-11-03	Primary care	RSV-A co-infection: RSV-A WG sequence not obtained (Ct RSV-A 24.55; Ct RSV-B 19.77; Age 14 y.o.a.)
EPI_ISL_16746475	hRSV/A/England/224720354/2022	2022-11-17	Primary care	hRSV-B co-infection: EPI_ISL_16746476 (Ct RSV-A 23.86; Ct RSV-B 24.50; Age <2 y.o.a.)
EPI_ISL_16746476	hRSV/B/England/224720354/2022	2022-11-17	Primary care	hRSV-A co-infection: EPI_ISL_16746475 (Ct RSV-A 23.86; Ct RSV-B 24.50; Age <2 y.o.a.)
EPI_ISL_16746477	hRSV/B/England/224720374/2022	2022-11-21	Primary care	
EPI_ISL_17258467	hRSV/A/England/223940503/2022	2022-09-27	Primary care	
EPI_ISL_17258468	hRSV/B/England/224520238/2022	2022-11-07	Primary care	
EPI_ISL_17258469	hRSV/B/England/224540252/2022	2022-11-07	Community	
EPI_ISL_17258470	hRSV/B/England/224540257/2022	2022-11-07	Community	
EPI_ISL_17258471	hRSV/B/England/224540707/2022	2022-11-07	Community	
EPI_ISL_17258472	hRSV/B/England/224560205/2022	2022-11-08	Primary care	
EPI_ISL_17258473	hRSV/B/England/224560219/2022	2022-11-07	Primary care	
EPI_ISL_17258474	hRSV/B/England/224560283/2022	2022-11-09	Community	
EPI_ISL_17258475	hRSV/B/England/224580224/2022	2022-11-09	Primary care	hAdV-C2: sequence unpublished
EPI_ISL_17258476	hRSV/B/England/224580227/2022	2022-11-09	Primary care	
EPI_ISL_17258477	hRSV/B/England/224600432/2022	2022-11-12	Community	
EPI_ISL_17258478	hRSV/B/England/224620379/2022	2022-11-14	Primary care	
EPI_ISL_17258479	hRSV/B/England/224620452/2022	2022-11-12	Community	
EPI_ISL_17258480	hRSV/B/England/224640350/2022	2022-11-14	Community	
EPI_ISL_17258481	hRSV/B/England/224640776/2022	2022-11-14	Primary care	Echovirus 9: sequence unpublished

GISAID_Accession_ID	GISAID_Virus_Name	Virus Collection Date	Source of Sample	Sequence confirmed co-infection comment (GISAID ID where available)
EPI_ISL_17258482	hRSV/B/England/224640784/2022	2022-11-14	Primary care	
EPI_ISL_17258483	hRSV/B/England/224660363/2022	2022-11-16	Primary care	
EPI_ISL_17258484	hRSV/B/England/224660370/2022	2022-11-15	Primary care	
EPI_ISL_17258485	hRSV/B/England/224660374/2022	2022-11-15	Primary care	
EPI_ISL_17258486	hRSV/B/England/224680469/2022	2022-11-16	Primary care	
EPI_ISL_17258487	hRSV/B/England/224680496/2022	2022-11-10	Primary care	
EPI_ISL_17258488	hRSV/B/England/224680501/2022	2022-11-17	Primary care	
EPI_ISL_17258489	hRSV/B/England/224680523/2022	2022-11-16	Community	
EPI_ISL_17258490	hRSV/B/England/224680531/2022	2022-11-15	Community	
EPI_ISL_17258491	hRSV/B/England/224680534/2022	2022-11-17	Community	
EPI_ISL_17258492	hRSV/B/England/224700528/2022	2022-11-18	Primary care	
EPI_ISL_17258493	hRSV/B/England/224700557/2022	2022-11-15	Primary care	
EPI_ISL_17258494	hRSV/B/England/224700560/2022	2022-11-17	Primary care	
EPI_ISL_17258495	hRSV/B/England/224720345/2022	2022-11-16	Primary care	
EPI_ISL_17258496	hRSV/B/England/224720368/2022	2022-11-18	Primary care	
EPI_ISL_17258497	hRSV/B/England/224740326/2022	2022-11-18	Primary care	
EPI_ISL_17258498	hRSV/B/England/224740336/2022	2022-11-21	Primary care	
EPI_ISL_17258499	hRSV/B/England/224740720/2022	2022-11-22	Community	
EPI_ISL_17258500	hRSV/B/England/224740724/2022	2022-11-22	Community	
EPI_ISL_17258501	hRSV/B/England/224760225/2022	2022-11-21	Primary care	Coxsackievirus A5: sequence unpublished
EPI_ISL_17258502	hRSV/B/England/224760231/2022	2022-11-21	Community	
EPI_ISL_17258503	hRSV/B/England/224800322/2022	2022-11-25	Primary care	
EPI_ISL_17258504	hRSV/B/England/224800328/2022	2022-11-23	Primary care	
EPI_ISL_17258505	hRSV/B/England/224800329/2022	2022-11-25	Primary care	
EPI_ISL_17258506	hRSV/B/England/224800331/2022	2022-11-22	Primary care	
EPI_ISL_17258507	hRSV/B/England/224800341/2022	2022-11-23	Primary care	
EPI_ISL_17258508	hRSV/B/England/224800344/2022	2022-11-22	Primary care	hAdV-C1: sequence unpublished
EPI_ISL_17258509	hRSV/B/England/224800354/2022	2022-11-24	Primary care	
EPI_ISL_17258510	hRSV/B/England/224800361/2022	2022-11-23	Primary care	
EPI_ISL_17258511	hRSV/B/England/224800362/2022	2022-11-22	Primary care	
EPI_ISL_17258512	hRSV/B/England/224800405/2022	2022-11-23	Community	
EPI_ISL_17258513	hRSV/B/England/224820368/2022	2022-11-23	Primary care	
EPI_ISL_17258514	hRSV/B/England/224820385/2022	2022-11-24	Primary care	
EPI_ISL_17258515	hRSV/B/England/224820391/2022	2022-11-25	Primary care	
EPI_ISL_17258516	hRSV/B/England/224820397/2022	2022-11-25	Primary care	
EPI_ISL_17258517	hRSV/B/England/224820414/2022	2022-11-23	Primary care	
EPI_ISL_17258518	hRSV/B/England/224820421/2022	2022-11-25	Primary care	
EPI_ISL_17258519	hRSV/B/England/224820916/2022	2022-11-29	Community	
EPI_ISL_17258520	hRSV/B/England/224860817/2022	2022-11-28	Primary care	
EPI_ISL_17258521	hRSV/B/England/224860825/2022	2022-11-24	Primary care	
EPI_ISL_17258522	hRSV/B/England/224860839/2022	2022-11-28	Primary care	
EPI_ISL_17258523	hRSV/B/England/224880437/2022	2022-11-21	Primary care	
EPI_ISL_17258524	hRSV/B/England/224880446/2022	2022-11-29	Primary care	
EPI_ISL_17258525	hRSV/B/England/224880466/2022	2022-11-22	Primary care	
EPI_ISL_17258526	hRSV/B/England/224880469/2022	2022-11-24	Primary care	

GISAID_Accession_ID	GISAID_Virus_Name	Virus Collection Date	Source of Sample	Sequence confirmed co-infection comment (GISAID ID where available)
EPI_ISL_17258527	hRSV/A/England/224880504/2022	2022-11-28	Community	
EPI_ISL_17258528	hRSV/A/England/224880505/2022	2022-11-28	Community	Enterovirus VP1 Genotype HRV-C40: sequence unpublished
EPI_ISL_17258529	hRSV/A/England/224880508/2022	2022-11-24	Community	
EPI_ISL_17258530	hRSV/A/England/224900422/2022	2022-11-29	Primary care	
EPI_ISL_17258531	hRSV/A/England/224900446/2022	2022-12-01	Primary care	
EPI_ISL_17258532	hRSV/A/England/224900469/2022	2022-11-29	Primary care	
EPI_ISL_17258533	hRSV/A/England/224900476/2022	2022-11-30	Primary care	
EPI_ISL_17258534	hRSV/A/England/224900479/2022	2022-11-29	Primary care	
EPI_ISL_17258535	hRSV/A/England/224900491/2022	2022-11-29	Primary care	
EPI_ISL_17258536	hRSV/A/England/224920377/2022	2022-12-02	Primary care	
EPI_ISL_17258537	hRSV/A/England/224920388/2022	2022-12-01	Primary care	
EPI_ISL_17258538	hRSV/A/England/224920395/2022	2022-12-01	Primary care	
EPI_ISL_17276529	hRSV/A/England/4940160/2022	2022-12-05	Primary care	
EPI_ISL_17276530	hRSV/A/England/4980787/2022	2022-12-06	Primary care	
EPI_ISL_17276531	hRSV/A/England/5000879/2022	2022-12-06	Secondary Care	
EPI_ISL_17276532	hRSV/B/England/5001045/2022	2022-12-07	Community	
EPI_ISL_17276533	hRSV/B/England/5001059/2022	2022-12-07	Community	
EPI_ISL_17276534	hRSV/B/England/5020399/2022	2022-12-07	Primary care	
EPI_ISL_17276535	hRSV/B/England/5020404/2022	2022-12-05	Primary care	
EPI_ISL_17276536	hRSV/B/England/5020434/2022	2022-12-08	Primary care	
EPI_ISL_17276537	hRSV/B/England/5020854/2022	2022-12-06	Primary care	
EPI_ISL_17276538	hRSV/A/England/5020859/2022	2022-12-06	Primary care	hRSV-B co-infection: EPI_ISL_17276539; Echovirus 9: sequence unpublished (Ct RSV-A 24.36; Ct RSV-B 27.82; Age 3 y.o.a.)
EPI_ISL_17276539	hRSV/B/England/5020859/2022	2022-12-06	Primary care	hRSV-A co-infection: EPI_ISL_17276538; Echovirus 9: sequence unpublished (Ct RSV-A 24.36; Ct RSV-B 27.82; Age 3 y.o.a.)
EPI_ISL_17276540	hRSV/B/England/5020861/2022	2022-12-08	Primary care	
EPI_ISL_17276541	hRSV/A/England/5020862/2022	2022-12-06	Primary care	
EPI_ISL_17276542	hRSV/A/England/5060138/2022	2022-12-07	Community	
EPI_ISL_17276543	hRSV/B/England/5060675/2022	2022-12-12	Primary care	
EPI_ISL_17276544	hRSV/B/England/5060711/2022	2022-12-12	Primary care	
EPI_ISL_17276545	hRSV/B/England/5060718/2022	2022-12-08	Primary care	
EPI_ISL_17276546	hRSV/A/England/5060720/2022	2022-12-06	Primary care	Coxsackievirus A4: sequence unpublished
EPI_ISL_17276547	hRSV/B/England/5060726/2022	2022-12-07	Primary care	
EPI_ISL_17276548	hRSV/A/England/5060746/2022	2022-12-12	Primary care	Enterovirus VP1 Genotype - HRV-C24: sequence unpublished
EPI_ISL_17276549	hRSV/B/England/5060749/2022	2022-12-12	Primary care	
EPI_ISL_17276550	hRSV/B/England/5080311/2022	2022-12-13	Primary care	
EPI_ISL_17276551	hRSV/B/England/5080347/2022	2022-12-12	Primary care	
EPI_ISL_17276552	hRSV/B/England/5100292/2022	2022-12-14	Primary care	
EPI_ISL_17276553	hRSV/A/England/5100392/2022	2022-12-13	Primary care	Coxsackievirus A9: sequence unpublished
EPI_ISL_17276554	hRSV/B/England/5100772/2022	2022-12-13	Primary care	
EPI_ISL_17276555	hRSV/A/England/5100796/2022	2022-12-16	Primary care	
EPI_ISL_17276556	hRSV/A/England/5100799/2022	2022-12-15	Primary care	
EPI_ISL_17276557	hRSV/B/England/5100817/2022	2022-12-06	Primary care	
EPI_ISL_17276558	hRSV/A/England/5100822/2022	2022-12-14	Primary care	
EPI_ISL_17276559	hRSV/B/England/5100854/2022	2022-12-15	Primary care	
EPI_ISL_17276560	hRSV/B/England/5100858/2022	2022-12-07	Primary care	

GISAID_Accession_ID	GISAID_Virus_Name	Virus Collection Date	Source of Sample	Sequence confirmed co-infection comment (GISAID ID where available)
EPI_ISL_17276561	hRSV/B/England/5100921/2022	2022-12-06	Primary care	
EPI_ISL_17276562	hRSV/B/England/5120153/2022	2022-12-09	Community	
EPI_ISL_17276563	hRSV/B/England/5120968/2022	2022-12-13	Primary care	
EPI_ISL_17276564	hRSV/A/England/5120972/2022	2022-12-15	Primary care	
EPI_ISL_17276565	hRSV/B/England/5120979/2022	2022-12-14	Primary care	Coxsackievirus A10: sequence unpublished
EPI_ISL_17276566	hRSV/A/England/5120993/2022	2022-12-01	Primary care	Coxsackievirus A4: sequence unpublished
EPI_ISL_17276567	hRSV/A/England/5121091/2022	2022-12-19	Primary care	Coxsackievirus A6: sequence unpublished
EPI_ISL_17276568	hRSV/A/England/5121092/2022	2022-12-16	Primary care	
EPI_ISL_17276569	hRSV/B/England/5121113/2022	2022-12-16	Primary care	
EPI_ISL_17276570	hRSV/A/England/5121133/2022	2022-12-13	Primary care	
EPI_ISL_17276571	hRSV/B/England/5121138/2022	2022-12-16	Primary care	
EPI_ISL_17276572	hRSV/B/England/5121241/2022	2022-12-13	Community	
EPI_ISL_17276573	hRSV/B/England/5121247/2022	2022-12-19	Community	
EPI_ISL_17276574	hRSV/B/England/5140712/2022	2022-12-19	Primary care	
EPI_ISL_17276575	hRSV/B/England/5140730/2022	2022-12-14	Primary care	
EPI_ISL_17276576	hRSV/B/England/5140746/2022	2022-12-19	Primary care	
EPI_ISL_17276577	hRSV/B/England/5140769/2022	2022-12-16	Primary care	
EPI_ISL_17276578	hRSV/B/England/5140770/2022	2022-12-13	Primary care	
EPI_ISL_17276579	hRSV/B/England/5140787/2022	2022-12-12	Primary care	Enterovirus VP1 Genotype - HRV-C24: sequence unpublished
EPI_ISL_17276580	hRSV/B/England/5141119/2022	2022-12-15	Primary care	
EPI_ISL_17276581	hRSV/B/England/5141156/2022	2022-12-19	Primary care	
EPI_ISL_17276582	hRSV/B/England/5141173/2022	2022-12-19	Primary care	
EPI_ISL_17276583	hRSV/B/England/5160081/2022	2022-12-19	Primary care	
EPI_ISL_17276584	hRSV/B/England/5160096/2022	2022-12-20	Primary care	
EPI_ISL_17276585	hRSV/B/England/5160413/2022	2022-12-15	Primary care	
EPI_ISL_17276586	hRSV/B/England/5160444/2022	2022-12-15	Primary care	
EPI_ISL_17276587	hRSV/B/England/5180024/2022	2022-12-20	Primary care	
EPI_ISL_17276588	hRSV/A/England/5180032/2022	2022-12-19	Primary care	
EPI_ISL_17276589	hRSV/B/England/5180045/2022	2022-12-21	Primary care	
EPI_ISL_17276590	hRSV/B/England/5180074/2022	2022-12-19	Primary care	
EPI_ISL_17276591	hRSV/B/England/5180078/2022	2022-12-19	Primary care	
EPI_ISL_17276592	hRSV/B/England/5180856/2022	2022-12-13	Community	
EPI_ISL_17276593	hRSV/B/England/5240023/2022	2022-12-16	Primary care	
EPI_ISL_17276594	hRSV/B/England/5240064/2022	2022-12-21	Primary care	
EPI_ISL_17276595	hRSV/B/England/5240069/2022	2022-12-22	Primary care	
EPI_ISL_17276596	hRSV/B/England/5240231/2022	2022-12-20	Primary care	
EPI_ISL_17276597	hRSV/B/England/5240233/2022	2022-12-20	Primary care	
EPI_ISL_17276598	hRSV/B/England/5240245/2022	2022-12-15	Primary care	
EPI_ISL_17276599	hRSV/B/England/5240355/2022	2022-12-20	Primary care	
EPI_ISL_17276600	hRSV/A/England/5260216/2022	2022-12-28	Primary care	
EPI_ISL_17276601	hRSV/A/England/5280305/2022	2022-12-28	Primary care	
EPI_ISL_17276602	hRSV/A/England/0140288/2022	2022-12-30	Primary care	
EPI_ISL_17276603	hRSV/A/England/0240406/2023	2023-01-09	Primary care	
EPI_ISL_17276604	hRSV/A/England/0240411/2023	2023-01-09	Primary care	
EPI_ISL_17276605	hRSV/A/England/0240436/2023	2023-01-10	Primary care	

GISAID_Accession_ID	GISAID_Virus_Name	Virus Collection Date	Source of Sample	Sequence confirmed co-infection comment (GISAID ID where available)
EPI_ISL_17276606	hRSV/A/England/0340203/2023	2023-01-16	Primary care	
EPI_ISL_17276607	hRSV/A/England/0400314/2023	2023-01-20	Primary care	
EPI_ISL_17276608	hRSV/A/England/0500427/2023	2023-01-26	Primary care	
EPI_ISL_17276609	hRSV/A/England/0520232/2023	2023-01-30	Primary care	
EPI_ISL_17276610	hRSV/A/England/0600808/2023	2023-01-15	Secondary Care	
EPI_ISL_17276611	hRSV/A/England/0600810/2023	2023-01-16	Secondary Care	
EPI_ISL_17276612	hRSV/A/England/0600812/2023	2023-01-10	Secondary Care	
EPI_ISL_17276613	hRSV/A/England/0600817/2023	2023-01-16	Secondary Care	
EPI_ISL_17276840	hRSV/B/England/5240547/2022	2022-12-21	Primary care	
EPI_ISL_17276841	hRSV/B/England/5240572/2022	2022-12-22	Community	
EPI_ISL_17276842	hRSV/B/England/5240603/2022	2022-12-13	Community	
EPI_ISL_17276843	hRSV/B/England/5260214/2022	2022-12-23	Primary care	
EPI_ISL_17276844	hRSV/B/England/5260234/2022	2022-12-21	Primary care	
EPI_ISL_17276845	hRSV/B/England/5260958/2022	2022-12-28	Primary care	
EPI_ISL_17276846	hRSV/B/England/5260963/2022	2022-12-16	Primary care	
EPI_ISL_17276847	hRSV/B/England/5261240/2022	2022-12-21	Primary care	
EPI_ISL_17276848	hRSV/B/England/5280279/2022	2022-12-30	Primary care	
EPI_ISL_17276849	hRSV/B/England/0120116/2022	2022-12-30	Primary care	
EPI_ISL_17276850	hRSV/B/England/0120426/2022	2022-12-29	Primary care	
EPI_ISL_17276851	hRSV/B/England/0120437/2022	2022-12-28	Primary care	
EPI_ISL_17276852	hRSV/B/England/0120438/2022	2022-12-29	Primary care	
EPI_ISL_17276853	hRSV/B/England/0120455/2022	2022-12-30	Primary care	
EPI_ISL_17276854	hRSV/B/England/0120751/2022	2022-12-23	Primary care	
EPI_ISL_17276855	hRSV/B/England/0120770/2022	2022-12-28	Primary care	
EPI_ISL_17276856	hRSV/B/England/0140265/2022	2022-12-30	Primary care	
EPI_ISL_17276857	hRSV/B/England/0140303/2022	2022-12-30	Primary care	
EPI_ISL_17276858	hRSV/B/England/0160774/2023	2023-01-03	Primary care	
EPI_ISL_17276859	hRSV/B/England/0180336/2023	2023-01-03	Primary care	
EPI_ISL_17276860	hRSV/B/England/0180348/2023	2023-01-03	Primary care	
EPI_ISL_17276861	hRSV/B/England/0180367/2023	2023-01-03	Primary care	
EPI_ISL_17276862	hRSV/B/England/0180398/2023	2023-01-05	Primary care	
EPI_ISL_17276863	hRSV/B/England/0180407/2023	2023-01-04	Primary care	
EPI_ISL_17276864	hRSV/B/England/0200537/2023	2023-01-04	Primary care	
EPI_ISL_17276865	hRSV/B/England/0200554/2023	2023-01-06	Primary care	
EPI_ISL_17276866	hRSV/B/England/0200561/2023	2023-01-05	Primary care	
EPI_ISL_17276867	hRSV/B/England/0200583/2023	2023-01-06	Primary care	
EPI_ISL_17276868	hRSV/B/England/0200589/2023	2023-01-05	Primary care	
EPI_ISL_17276869	hRSV/B/England/0200604/2023	2023-01-06	Primary care	
EPI_ISL_17276870	hRSV/B/England/0200620/2023	2023-01-05	Primary care	
EPI_ISL_17276871	hRSV/B/England/0200714/2023	2023-01-09	Community	
EPI_ISL_17276872	hRSV/B/England/0200764/2023	2023-01-07	Community	
EPI_ISL_17276873	hRSV/B/England/0220188/2023	2023-01-09	Primary care	
EPI_ISL_17276874	hRSV/B/England/0220196/2023	2023-01-09	Primary care	
EPI_ISL_17276875	hRSV/B/England/0240660/2023	2023-01-10	Community	
EPI_ISL_17276876	hRSV/B/England/0260154/2023	2023-01-09	Primary care	

GISAID_Accession_ID	GISAID_Virus_Name	Virus Collection Date	Source of Sample	Sequence confirmed co-infection comment (GISAID ID where available)
EPI_ISL_17276877	hRSV/B/England/0260175/2023	2023-01-10	Primary care	
EPI_ISL_17276878	hRSV/B/England/0280623/2023	2023-01-12	Primary care	
EPI_ISL_17276879	hRSV/B/England/0320180/2023	2023-01-13	Primary care	
EPI_ISL_17276880	hRSV/B/England/0360192/2023	2023-01-17	Primary care	
EPI_ISL_17276881	hRSV/B/England/0360227/2023	2023-01-17	Primary care	
EPI_ISL_17276882	hRSV/B/England/0360231/2023	2023-01-17	Primary care	
EPI_ISL_17276883	hRSV/B/England/0380302/2023	2023-01-19	Primary care	
EPI_ISL_17276884	hRSV/B/England/0380317/2023	2023-01-06	Primary care	
EPI_ISL_17276885	hRSV/B/England/0440364/2023	2023-01-23	Primary care	
EPI_ISL_17276886	hRSV/B/England/0460435/2023	2023-01-25	Community	
EPI_ISL_17276887	hRSV/B/England/0500413/2023	2023-01-26	Primary care	
EPI_ISL_17276888	hRSV/B/England/0520255/2023	2023-01-30	Primary care	
EPI_ISL_17276889	hRSV/B/England/0520310/2023	2023-01-28	Community	
EPI_ISL_17276890	hRSV/B/England/0560297/2023	2023-01-30	Primary care	
EPI_ISL_17276891	hRSV/B/England/0560304/2023	2023-02-01	Primary care	
EPI_ISL_17276892	hRSV/B/England/0580301/2023	2023-02-02	Primary care	
EPI_ISL_17276893	hRSV/B/England/0600201/2023	2023-02-02	Community	
EPI_ISL_17276894	hRSV/B/England/0600809/2023	2023-01-05	Secondary Care	
EPI_ISL_17276895	hRSV/B/England/0600813/2023	2023-01-22	Secondary Care	
EPI_ISL_17276896	hRSV/B/England/0600814/2023	2023-01-04	Secondary Care	
EPI_ISL_17276897	hRSV/B/England/0600815/2023	2023-01-04	Secondary Care	
EPI_ISL_17276898	hRSV/B/England/0600816/2023	2023-01-24	Secondary Care	
EPI_ISL_17276899	hRSV/B/England/0600818/2023	2023-01-16	Secondary Care	
EPI_ISL_17276900	hRSV/B/England/0600819/2022	2022-12-29	Secondary Care	
EPI_ISL_17276901	hRSV/B/England/0600820/2023	2023-01-09	Secondary Care	
EPI_ISL_17276902	hRSV/B/England/0600821/2022	2022-12-22	Secondary Care	
EPI_ISL_17276903	hRSV/B/England/0640786/2023	2023-02-07	Primary care	
EPI_ISL_17276904	hRSV/B/England/0660235/2023	2023-02-08	Primary care	
EPI_ISL_17276905	hRSV/B/England/0680167/2023	2023-02-08	Primary care	
EPI_ISL_17276906	hRSV/B/England/0700223/2023	2023-02-10	Primary care	
EPI_ISL_17276907	hRSV/B/England/0700231/2023	2023-02-08	Primary care	
EPI_ISL_17276908	hRSV/B/England/0700281/2023	2023-02-10	Primary care	
EPI_ISL_17276909	hRSV/B/England/0760185/2023	2023-02-15	Primary care	
EPI_ISL_17276910	hRSV/B/England/0780209/2023	2023-02-16	Primary care	
EPI_ISL_17276911	hRSV/B/England/0780227/2023	2023-02-15	Primary care	
EPI_ISL_17276912	hRSV/B/England/0800153/2023	2023-02-17	Primary care	
EPI_ISL_17276913	hRSV/B/England/0820176/2023	2023-02-18	Primary care	
EPI_ISL_17276914	hRSV/B/England/0840190/2023	2023-02-21	Primary care	
EPI_ISL_17276915	hRSV/B/England/0860254/2023	2023-02-20	Primary care	
EPI_ISL_17287519	hRSV/B/England/3941323/2021	2021-09-28	Primary care	
EPI_ISL_17287520	hRSV/B/England/0190800/2022	2022-01-07	Primary care	
EPI_ISL_17287521	hRSV/B/England/0521134/2022	2022-01-31	Primary care	
EPI_ISL_17287522	hRSV/B/England/3900359/2022	2022-09-23	Primary care	
EPI_ISL_17287523	hRSV/B/England/3920655/2022	2022-09-25	Community	
EPI_ISL_17287524	hRSV/A/England/4960442/2022	2022-12-06	Primary care	hRSV-B co-infection: EPI_ISL_17287525 (Ct RSV-A 19.31; Ct RSV-B 21.91; Age 11 y.o.a.)

GISAID_Accession_ID	GISAID_Virus_Name	Virus Collection Date	Source of Sample	Sequence confirmed co-infection comment (GISAID ID where available)
EPI_ISL_17287525	hRSV/B/England/4960442/2022	2022-12-06	Primary care	hRSV-A co-infection: EPI_ISL_17287524 (Ct RSV-A 19.31; Ct RSV-B 21.91; Age 11 y.o.a.)
EPI_ISL_17287526	hRSV/B/England/E21001475/2021	2021-10-16	Secondary Care	
EPI_ISL_17297218	hRSV/B/England/224900480/2022	2022-11-28	Primary care	hRSV-A co-infection: EPI_ISL_16737029 (Ct RSV-A 22.3; Ct RSV-B 19.6; Age 2 y.o.a.)
EPI_ISL_17297219	hRSV/B/England/0780276/2022	2022-02-16	Primary care	
EPI_ISL_17297220	hRSV/B/England/1260990/2022	2022-03-23	Primary care	hAdV-C1: sequence unpublished
EPI_ISL_17297221	hRSV/B/England/2040508/2022	2022-05-17	Primary care	
EPI_ISL_17297222	hRSV/A/England/2340409/2022	2022-06-06	Primary care	
EPI_ISL_17297223	hRSV/B/England/2560665/2022	2022-06-21	Primary care	
EPI_ISL_17297224	hRSV/B/England/2580176/2022	2022-06-22	Primary care	
EPI_ISL_17297225	hRSV/B/England/3040362/2022	2022-07-21	Primary care	
EPI_ISL_17297226	hRSV/B/England/4400248/2022	2022-10-28	Community	
EPI_ISL_17297227	hRSV/A/England/4460212/2022	2022-11-01	Primary care	
EPI_ISL_17297228	hRSV/B/England/4600228/2022	2022-11-11	Primary care	
EPI_ISL_17297229	hRSV/B/England/4900457/2022	2022-11-25	Primary care	
EPI_ISL_17297230	hRSV/B/England/4920348/2022	2022-11-29	Primary care	
EPI_ISL_17297231	hRSV/A/England/4940170/2022	2022-12-02	Primary care	
EPI_ISL_17297232	hRSV/B/England/4940192/2022	2022-12-05	Primary care	
EPI_ISL_17297233	hRSV/A/England/4960718/2022	2022-12-06	Primary care	
EPI_ISL_17297234	hRSV/A/England/5000802/2022	2022-12-07	Primary care	
EPI_ISL_17297235	hRSV/B/England/5020721/2022	2022-12-06	Secondary Care	
EPI_ISL_17297236	hRSV/B/England/5020893/2022	2022-12-09	Community	
EPI_ISL_17297237	hRSV/B/England/5080330/2022	2022-12-05	Primary care	IAV(H3N2): 3C.2a1b.2a.2: EPI_ISL_16745145
EPI_ISL_17297238	hRSV/B/England/5100324/2022	2022-12-14	Primary care	IAV(H3N2): 3C.2a1b.2a.2: EPI_ISL_16749477
EPI_ISL_17297239	hRSV/B/England/5121079/2022	2022-12-14	Primary care	IAV(H3N2): 3C.2a1b.2a.2: EPI_ISL_16745173
EPI_ISL_17297240	hRSV/B/England/5140707/2022	2022-12-14	Primary care	IAV(H3N2): 3C.2a1b.2a.2: 121E: EPI_ISL_17224995
EPI_ISL_17297241	hRSV/B/England/0120105/2023	2023-01-03	Primary care	SARS CoV-2: V-22OCT-01 PROBABLE Omicron BQ.1: EPI_ISL_16939117
EPI_ISL_17297242	hRSV/A/England/0120451/2022	2022-12-28	Primary care	
EPI_ISL_17297243	hRSV/B/England/0260156/2023	2023-01-11	Primary care	
EPI_ISL_17297244	hRSV/B/England/0280472/2023	2023-01-10	Primary care	
EPI_ISL_17297245	hRSV/B/England/0320201/2023	2023-01-16	Primary care	
EPI_ISL_17297246	hRSV/B/England/0340352/2023	2023-01-13	Community	
EPI_ISL_17297247	hRSV/A/England/0860266/2023	2023-02-22	Primary care	
EPI_ISL_17297248	hRSV/B/England/0860274/2023	2023-02-21	Primary care	
EPI_ISL_17297249	hRSV/B/England/0860276/2023	2023-02-21	Primary care	
EPI_ISL_17297250	hRSV/B/England/0880145/2023	2023-02-23	Primary care	
EPI_ISL_17297251	hRSV/B/England/0960142/2023	2023-02-27	Primary care	
EPI_ISL_17297252	hRSV/B/England/0960224/2023	2023-03-01	Community	
EPI_ISL_17297253	hRSV/A/England/0960586/2023	2023-02-27	Primary care	
EPI_ISL_1520413	hRSV/A/England/RE19003122/2019	2019-11-12	Secondary Care	
EPI_ISL_1520414	hRSV/A/England/RE19003123/2019	2019-11-17	Secondary Care	
EPI_ISL_1520415	hRSV/A/England/RE19003124/2019	2019-11-30	Secondary Care	
EPI_ISL_1520416	hRSV/A/England/RE19003125/2019	2019-11-13	Secondary Care	
EPI_ISL_1520418	hRSV/A/England/RE19003130/2019	2019-12-04	Secondary Care	
EPI_ISL_1520419	hRSV/A/England/RE19003131/2019	2019-10-21	Secondary Care	

GISAID_Accession_ID	GISAID_Virus_Name	Virus Collection Date	Source of Sample	Sequence confirmed co-infection comment (GISAID ID where available)
EPI_ISL_1520420	hRSV/A/England/RE19003132/2019	2019-11-23	Secondary Care	
EPI_ISL_1520421	hRSV/A/England/RE19003133/2019	2019-11-25	Secondary Care	
EPI_ISL_1520422	hRSV/A/England/RE19003137/2019	2019-12-04	Secondary Care	
EPI_ISL_1520423	hRSV/A/England/RE19003138/2019	2019-12-02	Secondary Care	
EPI_ISL_1520424	hRSV/A/England/RE19003141/2019	2019-12-05	Secondary Care	
EPI_ISL_1520426	hRSV/A/England/RE19003149/2019	2019-11-22	Secondary Care	
EPI_ISL_1647394	hRSV/A/England/RE19003126/2019	2019-11-15	Secondary Care	
EPI_ISL_1647395	hRSV/A/England/RE19003127/2019	2019-11-27	Secondary Care	
EPI_ISL_1647396	hRSV/A/England/RE19003136/2019	2019-11-16	Secondary Care	
EPI_ISL_1647397	hRSV/A/England/RE19003139/2019	2019-12-01	Secondary Care	
EPI_ISL_1647398	hRSV/A/England/RE19003140/2019	2019-11-04	Secondary Care	
EPI_ISL_1647400	hRSV/A/England/RE19003147/2019	2019-12-02	Secondary Care	
EPI_ISL_1647401	hRSV/A/England/RE19003148/2019	2019-11-20	Secondary Care	
EPI_ISL_1647402	hRSV/A/England/RE19003150/2019	2019-11-15	Secondary Care	
EPI_ISL_1647492	hRSV/B/England/194360327/2019	2019-10-22	Primary care	
EPI_ISL_1647493	hRSV/B/England/194360328/2019	2019-10-22	Primary care	
EPI_ISL_1647499	hRSV/B/England/194480340/2019	2019-10-30	Primary care	
EPI_ISL_1647500	hRSV/B/England/194480341/2019	2019-10-30	Primary care	
EPI_ISL_1647504	hRSV/B/England/194560624/2019	2019-11-06	Primary care	
EPI_ISL_1647505	hRSV/B/England/194560636/2019	2019-11-04	Primary care	
EPI_ISL_1647506	hRSV/B/England/194560645/2019	2019-11-05	Primary care	
EPI_ISL_1647531	hRSV/B/England/194860182/2019	2019-11-26	Primary care	
EPI_ISL_1647535	hRSV/B/England/194860199/2019	2019-11-25	Primary care	
EPI_ISL_1647536	hRSV/B/England/194860469/2019	2019-11-26	Primary care	
EPI_ISL_1647545	hRSV/B/England/194960704/2019	2019-12-02	Primary care	
EPI_ISL_1834163	hRSV/A/England/RE19003128/2019	2019-11-26	Secondary Care	
EPI_ISL_6494789	hRSV/B/England/145060047/2014	2014-12-08	Primary care	
EPI_ISL_6494812	hRSV/B/England/164240326/2016	2016-10-19	Primary care	
EPI_ISL_6494814	hRSV/B/England/164300018/2016	2016-10-20	Primary care	
EPI_ISL_6494817	hRSV/B/England/164440772/2016	2016-11-02	Primary care	
EPI_ISL_6494826	hRSV/B/England/165100030/2016	2016-12-13	Primary care	
