# Supplementary material for: Robust and sensitive amplicon-based whole-genome sequencing assay of respiratory syncytial virus subtype A and B
Source: Microbiol Spectr. 2024 Feb 27;12(4):e03067-23. doi: 10.1128/spectrum.03067-23 (PMC10986592; doi:10.1128/spectrum.03067-23)
Supplement: Table S4 — RSVA clinical sensitivity. [file spectrum.03067-23-s0007.pdf]

Robust and sensitive amplicon based whole genome sequencing assay of respiratory syncytial virus (RSV) subtype A and B

Supplementary material Table S4

Columns: Comparator assay and comparator pipeline

	Table_S4: Clinical Sensitivity/ Specificity of 21 previously sequenced RSV-A clinical samples	hRSV/A/England/RE19003122/2019	hRSV/A/England/RE19003123/2019	hRSV/A/England/RE19003124/2019	hRSV/A/England/RE19003125/2019	hRSV/A/England/RE19003126/2019	hRSV/A/England/RE19003127/2019	hRSV/A/England/RE19003128/2019	hRSV/A/England/RE19003130/2019	hRSV/A/England/RE19003131/2019	hRSV/A/England/RE19003132/2019	hRSV/A/England/RE19003133/2019	hRSV/A/England/RE19003136/2019	hRSV/A/England/RE19003137/2019	hRSV/A/England/RE19003138/2019	hRSV/A/England/RE19003139/2019	hRSV/A/England/RE19003140/2019	hRSV/A/England/RE19003141/2019	hRSV/A/England/RE19003147/2019	hRSV/A/England/RE19003148/2019	hRSV/A/England/RE19003149/2019	hRSV/A/England/RE19003150/2019
Comparator assay and comparator pipeline	hRSV/A/England/RE19003122/2019	100																				
	hRSV/A/England/RE19003123/2019	99.34	100																			
	hRSV/A/England/RE19003124/2019	99.97	99.35	100																		
	hRSV/A/England/RE19003125/2019	100	99.34	99.97	100																	
	hRSV/A/England/RE19003126/2019	99.97	99.34	99.97	99.97	100																
	hRSV/A/England/RE19003127/2019	99.97	99.35	99.98	99.97	99.96	100															
	hRSV/A/England/RE19003128/2019	99.95	99.33	99.97	99.95	99.95	99.96	100														
	hRSV/A/England/RE19003130/2019	99.98	99.36	99.99	99.98	99.97	99.99	99.97	100													
	hRSV/A/England/RE19003131/2019	99.97	99.35	99.99	99.97	99.97	99.98	99.97	99.99	100												
	hRSV/A/England/RE19003132/2019	99.94	99.32	99.95	99.94	99.93	99.95	99.93	99.96	99.95	100											
	hRSV/A/England/RE19003133/2019	99.97	99.35	99.99	99.97	99.97	99.98	99.97	99.99	99.99	99.95	100										
	hRSV/A/England/RE19003136/2019	99.99	99.35	99.99	99.99	99.98	99.98	99.97	99.99	99.99	99.95	99.99	100									
	hRSV/A/England/RE19003137/2019	99.96	99.33	99.96	99.96	99.97	99.95	99.94	99.97	99.96	99.93	99.96	99.97	100								
	hRSV/A/England/RE19003138/2019	99.97	99.35	99.98	99.97	99.96	99.97	99.96	99.99	99.98	99.95	99.98	99.98	99.95	100							
	hRSV/A/England/RE19003139/2019	99.97	99.35	99.98	99.97	99.96	99.97	99.96	99.99	99.98	99.95	99.98	99.98	99.95	99.97	100						
	hRSV/A/England/RE19003140/2019	99.98	99.36	99.99	99.98	99.97	99.99	99.97	100	99.99	99.96	99.99	99.99	99.97	99.99	99.99	100					
	hRSV/A/England/RE19003141/2019	99.97	99.35	99.98	99.97	99.96	99.97	99.96	99.99	99.98	99.95	99.98	99.98	99.95	99.97	99.97	99.99	100				
	hRSV/A/England/RE19003147/2019	99.31	99.92	99.33	99.31	99.31	99.32	99.31	99.33	99.33	99.29	99.33	99.33	99.31	99.32	99.32	99.33	99.32	100			
	hRSV/A/England/RE19003148/2019	99.97	99.35	99.98	99.97	99.97	99.97	99.96	99.99	99.98	99.95	99.98	99.98	99.97	99.97	99.97	99.99	99.97	99.33	100		
	hRSV/A/England/RE19003149/2019	99.97	99.34	99.97	99.97	99.97	99.96	99.95	99.97	99.97	99.93	99.97	99.98	99.97	99.96	99.96	99.97	99.96	99.31	99.97	100	
	hRSV/A/England/RE19003150/2019	99.97	99.35	99.98	99.97	99.96	99.97	99.96	99.99	99.98	99.95	99.98	99.98	99.95	99.97	99.97	99.99	99.97	99.32	99.97	99.96	100
Comparator assay and current pipeline	hRSV/A/England/RE19003122/2019	99.99	99.33	99.97	99.99	99.96	99.96	99.95	99.97	99.97	99.93	99.97	99.98	99.95	99.96	99.96	99.97	99.96	99.31	99.96	99.96	99.96
	hRSV/A/England/RE19003123/2019	99.34	100	99.35	99.34	99.34	99.35	99.33	99.36	99.35	99.32	99.35	99.35	99.33	99.35	99.35	99.36	99.35	99.92	99.35	99.34	99.35
	hRSV/A/England/RE19003124/2019	99.97	99.35	100	99.97	99.97	99.98	99.97	99.99	99.99	99.95	99.99	99.99	99.96	99.98	99.98	99.99	99.98	99.33	99.98	99.97	99.98
	hRSV/A/England/RE19003125/2019	99.99	99.33	99.97	99.99	99.96	99.96	99.95	99.97	99.97	99.93	99.97	99.98	99.95	99.96	99.96	99.97	99.96	99.31	99.96	99.96	99.96
	hRSV/A/England/RE19003126/2019	99.97	99.34	99.97	99.97	99.99	99.97	99.95	99.98	99.97	99.94	99.97	99.99	99.96	99.97	99.97	99.98	99.97	99.31	99.97	99.97	99.97
	hRSV/A/England/RE19003127/2019	99.97	99.35	99.98	99.97	99.96	100	99.96	99.99	99.98	99.95	99.98	99.98	99.95	99.97	99.97	99.99	99.97	99.32	99.97	99.96	99.97
	hRSV/A/England/RE19003128/2019	99.88	99.26	99.89	99.88	99.87	99.89	99.93	99.90	99.89	99.86	99.89	99.89	99.87	99.89	99.89	99.90	99.89	99.29	99.89	99.87	99.89
	hRSV/A/England/RE19003130/2019	99.98	99.36	99.99	99.98	99.97	99.99	99.97	100	99.99	99.96	99.99	99.99	99.97	99.99	99.99	100	99.99	99.33	99.99	99.97	99.99
	hRSV/A/England/RE19003131/2019	99.97	99.35	99.99	99.97	99.97	99.98	99.97	99.99	100	99.95	99.99	99.99	99.96	99.98	99.98	99.99	99.98	99.33	99.98	99.97	99.98
	hRSV/A/England/RE19003132/2019	99.94	99.32	99.95	99.94	99.93	99.95	99.93	99.96	99.95	100	99.95	99.95	99.93	99.95	99.95	99.96	99.95	99.29	99.95	99.93	99.95
	hRSV/A/England/RE19003133/2019	99.97	99.35	99.99	99.97	99.97	99.98	99.97	99.99	99.99	99.95	100	99.99	99.96	99.98	99.98	99.99	99.98	99.33	99.98	99.97	99.98
	hRSV/A/England/RE19003136/2019	99.99	99.35	99.99	99.99	99.98	99.98	99.97	99.99	99.99	99.95	99.99	100	99.97	99.98	99.98	99.99	99.98	99.33	99.98	99.98	99.98
	hRSV/A/England/RE19003137/2019	99.97	99.33	99.97	99.97	99.96	99.96	99.95	99.97	99.97	99.93	99.97	99.98	99.99	99.96	99.96	99.97	99.96	99.31	99.96	99.96	99.96
	hRSV/A/England/RE19003138/2019	99.97	99.35	99.98	99.97	99.96	99.97	99.96	99.99	99.98	99.95	99.98	99.98	99.95	100	99.97	99.99	99.97	99.32	99.97	99.96	99.97
	hRSV/A/England/RE19003139/2019	99.55	98.94	99.57	99.55	99.55	99.56	99.55	99.58	99.57	99.53	99.57	99.57	99.54	99.56	99.59	99.58	99.56	98.97	99.56	99.55	99.56
	hRSV/A/England/RE19003140/2019	99.98	99.36	99.99	99.98	99.97	99.99	99.97	100	99.99	99.96	99.99	99.99	99.97	99.99	99.99	100	99.99	99.33	99.99	99.97	99.99
	hRSV/A/England/RE19003141/2019	99.97	99.35	99.98	99.97	99.96	99.97	99.96	99.99	99.98	99.95	99.98	99.98	99.95	99.97	99.97	99.99	100	99.32	99.97	99.96	99.97
	hRSV/A/England/RE19003147/2019	99.00	99.61	99.02	99.00	99.00	99.01	99.00	99.02	99.02	98.98	99.02	99.02	99.00	99.01	99.01	99.02	99.01	99.69	99.02	99.00	99.01
	hRSV/A/England/RE19003148/2019	99.97	99.35	99.99	99.97	99.97	99.98	99.97	99.99	99.99	99.95	99.99	99.99	99.96	99.98	99.98	99.99	99.98	99.33	99.99	99.97	99.98
	hRSV/A/England/RE19003149/2019	99.97	99.34	99.97	99.97	99.97	99.97	99.95	99.98	99.97	99.94	99.97	99.99	99.96	99.97	99.97	99.98	99.97	99.31	99.97	99.99	99.97
	hRSV/A/England/RE19003150/2019	99.97	99.35	99.98	99.97	99.96	99.97	99.96	99.99	99.98	99.95	99.98	99.98	99.95	99.97	99.97	99.99	99.97	99.32	99.97	99.96	100
Updated assay and current pipeline	hRSV/A/England/RE19003122/2019	99.98	99.36	99.99	99.98	99.97	99.99	99.97	100	99.99	99.96	99.99	99.99	99.97	99.99	99.99	100	99.99	99.33	99.99	99.97	99.99
	hRSV/A/England/RE19003123/2019	99.34	100	99.35	99.34	99.34	99.35	99.33	99.36	99.35	99.32	99.35	99.35	99.33	99.35	99.35	99.36	99.35	99.92	99.35	99.34	99.35
	hRSV/A/England/RE19003124/2019	99.97	99.35	100	99.97	99.97	99.98	99.97	99.99	99.99	99.95	99.99	99.99	99.96	99.98	99.98	99.99	99.98	99.33	99.98	99.97	99.98
	hRSV/A/England/RE19003125/2019	99.99	99.33	99.96	99.99	99.95	99.95	99.94	99.97	99.96	99.93	99.96	99.97	99.95	99.95	99.95	99.97	99.95	99.30	99.95	99.95	99.95
	hRSV/A/England/RE19003126/2019	99.97	99.34	99.97	99.97	99.99	99.97	99.95	99.98	99.97	99.94	99.97	99.99	99.96	99.97	99.97	99.98	99.97	99.31	99.97	99.97	99.97
	hRSV/A/England/RE19003127/2019	99.97	99.35	99.98	99.97	99.96	100	99.96	99.99	99.98	99.95	99.98	99.98	99.95	99.97	99.97	99.99	99.97	99.32	99.97	99.96	99.97
	hRSV/A/England/RE19003128/2019	99.95	99.33	99.97	99.95	99.95	99.96	100	99.97	99.97	99.93	99.97	99.97	99.94	99.96	99.96	99.97	99.96	99.31	99.96	99.95	99.96
	hRSV/A/England/RE19003130/2019	99.98	99.36	99.99	99.98	99.97	99.99	99.97	100	99.99	99.96	99.99	99.99	99.97	99.99	99.99	100	99.99	99.33	99.99	99.97	99.99
	hRSV/A/England/RE19003131/2019	99.97	99.35	99.99	99.97	99.97	99.98	99.97	99.99	100	99.95	99.99	99.99	99.96	99.98	99.98	99.99	99.98	99.33	99.98	99.97	99.98
	hRSV/A/England/RE19003132/2019	99.94	99.32	99.95	99.94	99.93	99.95	99.93	99.96	99.95	100	99.95	99.95	99.93	99.95	99.95	99.96	99.95	99.29	99.95	99.93	99.95
	hRSV/A/England/RE19003133/2019	99.97	99.35	99.99	99.97	99.97	99.98	99.97	99.99	99.99	99.95	100	99.99	99.96	99.98	99.98	99.99	99.98	99.33	99.98	99.97	99.98
	hRSV/A/England/RE19003136/2019	99.98	99.35	99.98	99.98	99.97	99.97	99.96	99.99	99.98	99.95	99.98	99.99	99.97	99.97	99.97	99.99	99.97	99.32	99.97	99.97	99.97
	hRSV/A/England/RE19003137/2019	99.97	99.33	99.97	99.97	99.96	99.96	99.95	99.97	99.97	99.93	99.97	99.98	99.99	99.96	99.96	99.97	99.96	99.31	99.96	99.96	99.96
	hRSV/A/England/RE19003138/2019	99.97	99.35	99.98	99.97	99.96	99.97	99.96	99.99	99.98	99.95	99.98	99.98	99.95	100	99.97	99.99	99.97	99.32	99.97	99.96	99.97
	hRSV/A/England/RE19003139/2019	99.97	99.35	99.98	99.97	99.96	99.97	99.96	99.99	99.98	99.95	99.98	99.98	99.95	99.97	100	99.99	99.97	99.32	99.97	99.96	99.97
	hRSV/A/England/RE19003140/2019	99.97	99.35	99.99	99.97	99.97	99.98	99.97	99.99	99.99	99.96	99.99	99.99	99.96	99.98	99.98	99.99	99.98	99.33	99.98	99.97	99.98
	hRSV/A/England/RE19003141/2019	99.97	99.35	99.98	99.97	99.96	99.97	99.96	99.99	99.98	99.95	99.98	99.98	99.95	99.97	99.97	99.99	100	99.32	99.97	99.96	99.97
	hRSV/A/England/RE19003147/2019	99.34	99.95	99.35	99.34	99.34	99.35	99.33	99.36	99.35	99.32	99.35	99.35	99.33	99.35	99.35	99.36	99.35	99.97	99.35	99.34	99.35
	hRSV/A/England/RE19003148/2019	99.97	99.35	99.99	99.97	99.97	99.98	99.97	99.99	99.99	99.95	99.99	99.99	99.96	99.98	99.98	99.99	99.98	99.33	99.99	99.97	99.98
	hRSV/A/England/RE19003149/2019	99.97	99.33	99.97	99.97	99.96	99.96	99.95	99.97	99.97	99.93	99.97	99.98	99.95	99.96	99.96	99.97	99.96	99.31	99.96	99.99	99.96
	hRSV/A/England/RE19003150/2019	99.97	99.35	99.98	99.97	99.96	99.97	99.96	99.99	99.98	99.95	99.98	99.98	99.95	99.97	99.97	99.99	99.97	99.32	99.97	99.96	100

Columns: Comparator assay and current pipeline

Comparator assay and comparator pipeline rows:

Mean Percent Identity: 99.960

Max Percent Identity: 100

Min Percent Identity: 99.590

SD Percent Identity: 0.108

95% Confidence Interval 0.948 to 0.999

	Table_S4: Clinical Sensitivity/ Specificity of 21 previously sequenced RSV-A clinical samples	hRSV/A/England/RE19003122/2019	hRSV/A/England/RE19003123/2019	hRSV/A/England/RE19003124/2019	hRSV/A/England/RE19003125/2019	hRSV/A/England/RE19003126/2019	hRSV/A/England/RE19003127/2019	hRSV/A/England/RE19003128/2019	hRSV/A/England/RE19003130/2019	hRSV/A/England/RE19003131/2019	hRSV/A/England/RE19003132/2019	hRSV/A/England/RE19003133/2019	hRSV/A/England/RE19003136/2019	hRSV/A/England/RE19003137/2019	hRSV/A/England/RE19003138/2019	hRSV/A/England/RE19003139/2019	hRSV/A/England/RE19003140/2019	hRSV/A/England/RE19003141/2019	hRSV/A/England/RE19003147/2019	hRSV/A/England/RE19003148/2019	hRSV/A/England/RE19003149/2019	hRSV/A/England/RE19003150/2019
Comparator assay and current pipeline	hRSV/A/England/RE19003122/2019	100																				
	hRSV/A/England/RE19003123/2019	99.33	100																			
	hRSV/A/England/RE19003124/2019	99.97	99.35	100																		
	hRSV/A/England/RE19003125/2019	100	99.33	99.97	100																	
	hRSV/A/England/RE19003126/2019	99.97	99.34	99.97	99.97	100																
	hRSV/A/England/RE19003127/2019	99.96	99.35	99.98	99.96	99.97	100															
	hRSV/A/England/RE19003128/2019	99.87	99.26	99.89	99.87	99.88	99.89	100														
	hRSV/A/England/RE19003130/2019	99.97	99.36	99.99	99.97	99.98	99.99	99.90	100													
	hRSV/A/England/RE19003131/2019	99.97	99.35	99.99	99.97	99.97	99.98	99.89	99.99	100												
	hRSV/A/England/RE19003132/2019	99.93	99.32	99.95	99.93	99.94	99.95	99.86	99.96	99.95	100											
	hRSV/A/England/RE19003133/2019	99.97	99.35	99.99	99.97	99.97	99.98	99.89	99.99	99.99	99.95	100										
	hRSV/A/England/RE19003136/2019	99.98	99.35	99.99	99.98	99.99	99.98	99.89	99.99	99.99	99.95	99.99	100									
	hRSV/A/England/RE19003137/2019	99.96	99.33	99.97	99.96	99.97	99.96	99.87	99.97	99.97	99.93	99.97	99.98	100								
	hRSV/A/England/RE19003138/2019	99.96	99.35	99.98	99.96	99.97	99.97	99.89	99.99	99.98	99.95	99.98	99.98	99.96	100							
	hRSV/A/England/RE19003139/2019	99.55	98.94	99.57	99.55	99.55	99.56	99.62	99.58	99.57	99.53	99.57	99.57	99.55	99.56	100						
	hRSV/A/England/RE19003140/2019	99.97	99.36	99.99	99.97	99.98	99.99	99.90	100	99.99	99.96	99.99	99.99	99.97	99.99	99.58	100					
	hRSV/A/England/RE19003141/2019	99.96	99.35	99.98	99.96	99.97	99.97	99.89	99.99	99.98	99.95	99.98	99.98	99.96	99.97	99.56	99.99	100				
	hRSV/A/England/RE19003147/2019	99.00	99.61	99.02	99.00	99.00	99.01	99.07	99.02	99.02	98.98	99.02	99.02	99.00	99.01	99.10	99.02	99.01	100			
	hRSV/A/England/RE19003148/2019	99.97	99.35	99.99	99.97	99.97	99.98	99.89	99.99	99.99	99.95	99.99	99.99	99.97	99.98	99.57	99.99	99.98	99.02	100		
	hRSV/A/England/RE19003149/2019	99.97	99.34	99.97	99.97	99.97	99.97	99.88	99.98	99.97	99.94	99.97	99.99	99.97	99.97	99.55	99.98	99.97	99.00	99.97	100	
	hRSV/A/England/RE19003150/2019	99.96	99.35	99.98	99.96	99.97	99.97	99.89	99.99	99.98	99.95	99.98	99.98	99.96	99.97	99.56	99.99	99.97	99.01	99.98	99.97	100
Updated assay and current pipeline	hRSV/A/England/RE19003122/2019	99.97	99.36	99.99	99.97	99.98	99.99	99.90	100	99.99	99.96	99.99	99.99	99.97	99.99	99.58	100	99.99	99.02	99.99	99.98	99.99
	hRSV/A/England/RE19003123/2019	99.33	100	99.35	99.33	99.34	99.35	99.26	99.36	99.35	99.32	99.35	99.35	99.33	99.35	98.94	99.36	99.35	99.61	99.35	99.34	99.35
	hRSV/A/England/RE19003124/2019	99.97	99.35	100	99.97	99.97	99.98	99.89	99.99	99.99	99.95	99.99	99.99	99.97	99.98	99.57	99.99	99.98	99.02	99.99	99.97	99.98
	hRSV/A/England/RE19003125/2019	99.98	99.33	99.96	99.98	99.96	99.95	99.87	99.97	99.96	99.93	99.96	99.97	99.95	99.95	99.54	99.97	99.95	98.99	99.96	99.96	99.95
	hRSV/A/England/RE19003126/2019	99.97	99.34	99.97	99.97	100	99.97	99.88	99.98	99.97	99.94	99.97	99.99	99.97	99.97	99.55	99.98	99.97	99.00	99.97	99.97	99.97
	hRSV/A/England/RE19003127/2019	99.96	99.35	99.98	99.96	99.97	100	99.89	99.99	99.98	99.95	99.98	99.98	99.96	99.97	99.56	99.99	99.97	99.01	99.98	99.97	99.97
	hRSV/A/England/RE19003128/2019	99.95	99.33	99.97	99.95	99.95	99.96	99.93	99.97	99.97	99.93	99.97	99.97	99.95	99.96	99.55	99.97	99.96	99.00	99.97	99.95	99.96
	hRSV/A/England/RE19003130/2019	99.97	99.36	99.99	99.97	99.98	99.99	99.90	100	99.99	99.96	99.99	99.99	99.97	99.99	99.58	100	99.99	99.02	99.99	99.98	99.99
	hRSV/A/England/RE19003131/2019	99.97	99.35	99.99	99.97	99.97	99.98	99.89	99.99	100	99.95	99.99	99.99	99.97	99.98	99.57	99.99	99.98	99.02	99.99	99.97	99.98
	hRSV/A/England/RE19003132/2019	99.93	99.32	99.95	99.93	99.94	99.95	99.86	99.96	99.95	100	99.95	99.95	99.93	99.95	99.53	99.96	99.95	98.98	99.95	99.94	99.95
	hRSV/A/England/RE19003133/2019	99.97	99.35	99.99	99.97	99.97	99.98	99.89	99.99	99.99	99.95	100	99.99	99.97	99.98	99.57	99.99	99.98	99.02	99.99	99.97	99.98
	hRSV/A/England/RE19003136/2019	99.97	99.35	99.98	99.97	99.98	99.97	99.89	99.99	99.98	99.95	99.98	99.99	99.97	99.97	99.56	99.99	99.97	99.02	99.98	99.98	99.97
	hRSV/A/England/RE19003137/2019	99.96	99.33	99.97	99.96	99.97	99.96	99.87	99.97	99.97	99.93	99.97	99.98	100	99.96	99.55	99.97	99.96	99.00	99.97	99.97	99.96
	hRSV/A/England/RE19003138/2019	99.96	99.35	99.98	99.96	99.97	99.97	99.89	99.99	99.98	99.95	99.98	99.98	99.96	100	99.56	99.99	99.97	99.01	99.98	99.97	99.97
	hRSV/A/England/RE19003139/2019	99.96	99.35	99.98	99.96	99.97	99.97	99.89	99.99	99.98	99.95	99.98	99.98	99.96	99.97	99.59	99.99	99.97	99.01	99.98	99.97	99.97
	hRSV/A/England/RE19003140/2019	99.97	99.35	99.99	99.97	99.97	99.98	99.89	99.99	99.99	99.96	99.99	99.99	99.97	99.98	99.57	99.99	99.98	99.02	99.99	99.97	99.98
	hRSV/A/England/RE19003141/2019	99.96	99.35	99.98	99.96	99.97	99.97	99.89	99.99	99.98	99.95	99.98	99.98	99.96	99.97	99.56	99.99	100	99.01	99.98	99.97	99.97
	hRSV/A/England/RE19003147/2019	99.33	99.95	99.35	99.33	99.34	99.35	99.26	99.36	99.35	99.32	99.35	99.35	99.33	99.35	98.94	99.36	99.35	99.66	99.35	99.34	99.35
	hRSV/A/England/RE19003148/2019	99.97	99.35	99.99	99.97	99.97	99.98	99.89	99.99	99.99	99.95	99.99	99.99	99.97	99.98	99.57	99.99	99.98	99.02	100	99.97	99.98
	hRSV/A/England/RE19003149/2019	99.96	99.33	99.97	99.96	99.97	99.96	99.87	99.97	99.97	99.93	99.97	99.98	99.96	99.96	99.55	99.97	99.96	99.00	99.97	99.99	99.96
	hRSV/A/England/RE19003150/2019	99.96	99.35	99.98	99.96	99.97	99.97	99.89	99.99	99.98	99.95	99.98	99.98	99.96	99.97	99.56	99.99	99.97	99.01	99.98	99.97	100

Columns: Updated assay and current pipeline

Comparator assay and comparator pipeline rows:

Mean Percent Identity: 99.994

Max Percent Identity: 100

Min Percent Identity: 99.970

SD Percent Identity: 0.008

95% Confidence Interval 0.996 to 1.000

Comparator assay and current pipeline rows:

Mean Percent Identity: 99.957

Max Percent Identity: 100

Min Percent Identity: 99.590

SD Percent Identity: 0.112

95% Confidence Interval 0.946 to 0.999

	Table_S4: Clinical Sensitivity/ Specificity of 21 previously sequenced RSV-A clinical samples	hRSV/A/England/RE19003122/2019	hRSV/A/England/RE19003123/2019	hRSV/A/England/RE19003124/2019	hRSV/A/England/RE19003125/2019	hRSV/A/England/RE19003126/2019	hRSV/A/England/RE19003127/2019	hRSV/A/England/RE19003128/2019	hRSV/A/England/RE19003130/2019	hRSV/A/England/RE19003131/2019	hRSV/A/England/RE19003132/2019	hRSV/A/England/RE19003133/2019	hRSV/A/England/RE19003136/2019	hRSV/A/England/RE19003137/2019	hRSV/A/England/RE19003138/2019	hRSV/A/England/RE19003139/2019	hRSV/A/England/RE19003140/2019	hRSV/A/England/RE19003141/2019	hRSV/A/England/RE19003147/2019	hRSV/A/England/RE19003148/2019	hRSV/A/England/RE19003149/2019	hRSV/A/England/RE19003150/2019
Updated assay and current pipeline	hRSV/A/England/RE19003122/2019	100																				
	hRSV/A/England/RE19003123/2019	99.36	100																			
	hRSV/A/England/RE19003124/2019	99.99	99.35	100																		
	hRSV/A/England/RE19003125/2019	99.97	99.33	99.96	100																	
	hRSV/A/England/RE19003126/2019	99.98	99.34	99.97	99.96	100																
	hRSV/A/England/RE19003127/2019	99.99	99.35	99.98	99.95	99.97	100															
	hRSV/A/England/RE19003128/2019	99.97	99.33	99.97	99.94	99.95	99.96	100														
	hRSV/A/England/RE19003130/2019	100	99.36	99.99	99.97	99.98	99.99	99.97	100													
	hRSV/A/England/RE19003131/2019	99.99	99.35	99.99	99.96	99.97	99.98	99.97	99.99	100												
	hRSV/A/England/RE19003132/2019	99.96	99.32	99.95	99.93	99.94	99.95	99.93	99.96	99.95	100											
	hRSV/A/England/RE19003133/2019	99.99	99.35	99.99	99.96	99.97	99.98	99.97	99.99	99.99	99.95	100										
	hRSV/A/England/RE19003136/2019	99.99	99.35	99.98	99.97	99.98	99.97	99.96	99.99	99.98	99.95	99.98	100									
	hRSV/A/England/RE19003137/2019	99.97	99.33	99.97	99.95	99.97	99.96	99.95	99.97	99.97	99.93	99.97	99.97	100								
	hRSV/A/England/RE19003138/2019	99.99	99.35	99.98	99.95	99.97	99.97	99.96	99.99	99.98	99.95	99.98	99.97	99.96	100							
	hRSV/A/England/RE19003139/2019	99.99	99.35	99.98	99.95	99.97	99.97	99.96	99.99	99.98	99.95	99.98	99.97	99.96	99.97	100						
	hRSV/A/England/RE19003140/2019	99.99	99.35	99.99	99.96	99.97	99.98	99.97	99.99	99.99	99.96	99.99	99.98	99.97	99.98	99.98	100					
	hRSV/A/England/RE19003141/2019	99.99	99.35	99.98	99.95	99.97	99.97	99.96	99.99	99.98	99.95	99.98	99.97	99.96	99.97	99.97	99.98	100				
	hRSV/A/England/RE19003147/2019	99.36	99.95	99.35	99.33	99.34	99.35	99.33	99.36	99.35	99.32	99.35	99.35	99.33	99.35	99.35	99.35	99.35	100			
	hRSV/A/England/RE19003148/2019	99.99	99.35	99.99	99.96	99.97	99.98	99.97	99.99	99.99	99.95	99.99	99.98	99.97	99.98	99.98	99.99	99.98	99.35	100		
	hRSV/A/England/RE19003149/2019	99.97	99.33	99.97	99.95	99.97	99.97	99.96	99.97	99.97	99.93	99.97	99.97	99.96	99.96	99.96	99.97	99.96	99.33	99.97	100	
	hRSV/A/England/RE19003150/2019	99.99	99.35	99.98	99.95	99.97	99.97	99.96	99.99	99.98	99.95	99.98	99.97	99.96	99.97	99.97	99.98	99.97	99.35	99.98	99.96	100
